# TriTower-m6Am: A triple-tower heterogeneous deep learning architecture integrating semantic, sequential, and structural information for mRNA N6,2′-O-dimethyladenosine site prediction

**DOI:** 10.1371/journal.pcbi.1014939

**Published:** 2026-11-05

**Authors:** Kaifeng Xiong, Jianhua Jia

**Affiliations:** School of Information Engineering, Jingdezhen Ceramic University, Jingdezhen, China; Indiana University, UNITED STATES OF AMERICA

## Abstract

**Background:**

N^6^,2′-O-dimethyladenosine (m6Am) is a cap-proximal mRNA modification deposited by PCIF1 (phosphorylated C-terminal domain (CTD)-interacting factor 1) at the first transcribed nucleotide of eukaryotic mRNAs. Knowing where m6Am sites sit would help explain how cells tune mRNA stability and translation, but current predictors typically depend on a single representation and do not jointly model multiple views of the same RNA sequence.

**Results:**

We present TriTower-m6Am, combining semantic (RNA-FM, a pretrained RNA foundation model, with BellPooling), sequential (One-Hot BiLSTM, bidirectional long short-term memory), and structural (RGCN, a relational graph convolutional network, over the 41-nt window) representations of each RNA window. On an independent test set of 640 sequences, TriTower-m6Am reaches an area under the ROC curve (AUC) of 0.776 and a Matthews correlation coefficient (MCC) of 0.425, against DTC-m6Am’s AUC = 0.765 and MCC = 0.411, using 4.4 times fewer trainable parameters; at every matched specificity between 0.40 and 0.70 it recovers 1–7 more true sites out of 320 than DTC-m6Am. The AUC-weighted voting keeps every tower’s contribution readable for any individual prediction. Analyses of the graph tower delimit its benefit to the relational aggregation of local backbone adjacency over sequence-derived node features: the edge-type ablation, the node-feature ablation, and a structure-permutation control agree that the specific RNAfold-predicted fold carries no attributable signal, and a probing analysis shows that per-base base-pairing status is largely recoverable from the sequence-derived RNA-FM embeddings (AUC = 0.72 vs. 0.65 from the raw sequence), so the fold annotation is substantially redundant with the semantic representation the ensemble already receives.

**Conclusions:**

Combining semantic, sequential, and structural views of the same RNA sequence improves m6Am prediction beyond any single representation, and the ensemble’s voting weights make each prediction auditable without a post-hoc explainer. Whether the triple-tower pattern transfers to other RNA modification prediction tasks remains to be tested in future work.

## Author summary

Predicting where m6Am modifications occur on messenger RNA is important for understanding how cells regulate transcript stability and translation. Existing computational methods typically encode the RNA sequence in a single way, such as k-mer counts or a one-hot code, and treat the model as a black box that emits a prediction without explaining which features drove it. We built TriTower-m6Am to address both limitations. Our model combines three independent encoders—a pretrained RNA language model for semantic patterns, a bidirectional LSTM for local nucleotide order, and a relational graph convolutional network for the structural fold—and fuses their outputs by AUC-weighted voting, so the contribution of each tower to a given prediction is a readable number rather than a hidden parameter. On an independent benchmark the ensemble achieves ranking quality comparable to the prior best method with 4.4 times fewer parameters, and its voting weights make each tower’s contribution auditable for any individual prediction. A controlled structural ablation shows that linear backbone adjacency, rather than long-range base-pairing, carries whatever signal the graph tower uses, and that randomizing the predicted secondary structures does not degrade performance. This controlled analysis delineates what the graph tower contributes: its benefit lies in the relational encoding of local adjacency over sequence-derived features, which the control retains, rather than in the specific predicted fold. Whether the same triple-tower pattern transfers to other RNA modification site prediction tasks is a question for future work.

## Introduction

Of the chemical modifications on eukaryotic mRNA, m6Am (N^6^,2′-O-dimethyladenosine) is unusual in that it is found only at the first transcribed nucleotide, and only when that nucleotide is an adenosine [1,2]. The modification is written by PCIF1 (phosphorylated C-terminal domain (CTD)-interacting factor 1, also called CAPAM for cap-specific adenosine methyltransferase) and erased by FTO (fat mass and obesity-associated protein) [3,4], which places m6Am among the reversible epitranscriptomic marks. Its cap-proximal position gives it a disproportionate functional role: m6Am reduces decapping, lengthens transcript half-life, and modulates translation, splicing, and disease-associated phenotypes [5–8]. This is distinct from internal marks such as N^6^-methyladenosine (m6A) or 5-methylcytosine (m5C), which decorate the transcript body without the same positional constraint.

Mapping m6Am at single-nucleotide resolution relies on antibody-based assays, chiefly miCLIP (methylation individual-nucleotide-resolution cross-linking and immunoprecipitation) and m6Am-seq [3,9]. These experiments are expensive and low-throughput, which has motivated a series of computational predictors. DTC-m6Am frames the task as an imbalanced-classification problem and pairs DenseNet with an attention module [10]. Deep-m6Am feeds pseudo nucleotide composition into a deep network and uses SHAP (SHapley Additive exPlanations) to prune features [11]. im6Am-DC swaps in coordinate attention and focal loss to cope with the skewed class ratio [12]. Each of these tools works, but each also represents the input sequence in only one way: k-mer statistics, hand-crafted physicochemical features, or a one-hot code. Three information channels that an RNA sequence carries—the semantic patterns learned by large-scale pre-training, the local sequential order of nucleotides, and the structural fold encoded by base-pairing and neighborhood relationships—are not used together in any of them. Secondary structure (SS), in particular, is largely set aside, on the assumption that the local fold governs how PCIF1 reaches its substrate. Whether that assumption holds has, to our knowledge, not been tested for m6Am directly. The third shortcoming is opacity: a prediction is emitted, but the feature basis for that prediction is not.

Pre-trained RNA language models such as RNA-FM (RNA foundation model) [13] and RNAErnie [14] offer one way to recover the semantic channel: trained on millions of sequences, they encode co-occurrence patterns that k-mer counts miss. They have not, however, been combined with an explicit sequential encoder and a structural graph in a single m6Am predictor, and the question of whether the three channels are complementary or redundant at this task has therefore not been answered.

**TriTower-m6Am** is built around that question. The model has three independent towers. The RNA-FM tower reads a 41-nt window with a pre-trained language model and pools the per-position embeddings with a learnable bell-shaped attention (BellPooling) centered on the candidate site. The One-Hot BiLSTM (bidirectional long short-term memory) tower consumes the same window as a 4-dimensional one-hot code and processes it bidirectionally, keeping the hidden state at the central position as its sequential summary. The RGCN (relational graph convolutional network) tower builds a graph over the 41 nucleotides with three edge types—backbone, base-pairing, and neighborhood—and runs a relational graph convolution that assigns each edge type its own weight matrix. The three tower outputs are fused by AUC-weighted voting, so the contribution of each tower is a readable number rather than a hidden parameter. On the DTC-m6Am benchmark (640 balanced independent test sequences), the ensemble reaches ranking quality comparable to the prior best method while using 4.4 times fewer parameters, and at matched specificity it consistently recovers 1–7 additional true sites per 320. Because the RGCN distinguishes edge types, the same ablation that validates the design also exposes which structural relationships the model relies on, so interpretation is a by-product of the architecture rather than an additional stage. In summary, TriTower-m6Am addresses the three shortcomings identified above with three explicit strategies: (1) a pretrained RNA language model (RNA-FM) to capture the semantic patterns that k-mer counts miss; (2) a BiLSTM to encode the local nucleotide order that one-hot convolutions treat only implicitly; and (3) a relational graph convolutional network with typed edges over the 41-nucleotide window, whose structural input we then probe directly, while the AUC-weighted voting weights make each tower’s contribution to a prediction directly auditable rather than opaque.

## Materials and methods

### Ethics statement

This study is a secondary analysis of publicly available, previously published sequencing datasets. No new human participants or human samples were recruited, and no animal experiments were performed. Ethical approval and informed consent were therefore not required.

### Dataset

All experiments use the benchmark released with DTC-m6Am [10], which we inherited unchanged. The benchmark was constructed by Liu et al. [15], who applied CD-HIT redundancy removal at 0.8 sequence identity to the dataset of Jiang et al. [16]. Its positive samples are miCLIP-confirmed m6Am sites from HEK293/HEK293T cells (GEO accessions GSE63753 [9] and GSE78040 [17]), and its negative samples are *unmodified BCA-motif adenosines* (B = C/G/U) defined by the same protocol: a non-A base at position −2, cytosine at −1, and the candidate adenosine at position 0.

A sample is a 41-nt window centred on the candidate adenosine (position 20, 0-indexed), with 20 nt of upstream and downstream context. The 41-nt extent is inherited rather than chosen by us: the entire method lineage against which we benchmark (m6AmPred, DLm6Am, m6Aminer, DTC-m6Am) uses the same window centred on the modification site, and ±20 nt is the conventional context length in RNA-modification prediction since SRAMP. We deliberately did not re-tune the window length, so that the comparison remains like-for-like; window-length ablation is left as future work.

Because the reference transcript set contains multiple 5^′^ isoforms per gene, the same adenosine can be the first transcribed nucleotide of a short isoform and an internal position of a longer one. For most windows, the 20 positions on the 5^′^ side of the candidate site are therefore the transcribed 5^′^ leader of a longer isoform rather than genomic promoter sequence. Consistent with this, the position immediately 5^′^ of the candidate is cytosine in 90.1% of positive windows, matching the mammalian initiator (Inr) consensus BBCAB in which PCIF1 acts.

Two constraints shape the dataset. First, the central nucleotide is adenosine in every sample, positive or negative, so the model cannot classify by reading the candidate base itself; it must extract signal from the flanking context. Second, the negative label is experimentally anchored: negatives are BCA-motif adenosines that were not called as modified in the same miCLIP experiments, rather than arbitrary adenosine positions.

The training set holds 40,700 windows (3,700 positives, 37,000 negatives; ≈10:1 ratio), and the held-out test set contains 640 windows balanced at 320 each (Table 1). The skewed ratio is biological in origin: among cap-proximal adenosines, only a minority carry the modification. Secondary structure for each window was predicted with RNAfold from the ViennaRNA package [18] and stored as dot-bracket strings, which the RGCN tower later parses into pairing and loop annotations.

**Table 1:** Dataset split and class distribution. “Pos.” = positive samples; “Neg.” = negative samples. The test set is balanced to give an unbiased estimate of per-class metrics.

Split	Pos.	Neg.	Total	Pos:Neg
Training	3,700	37,000	40,700	≈1:10
Test	320	320	640	1:1

### Overall architecture

The model is organized as three independent towers whose predictions are fused at the end (Fig 1). As sketched in Fig 1a, the pipeline runs from raw 41-nt window through representation extraction, 5-fold cross-validated training of each tower, and finally AUC-weighted voting.

**Fig 1:**
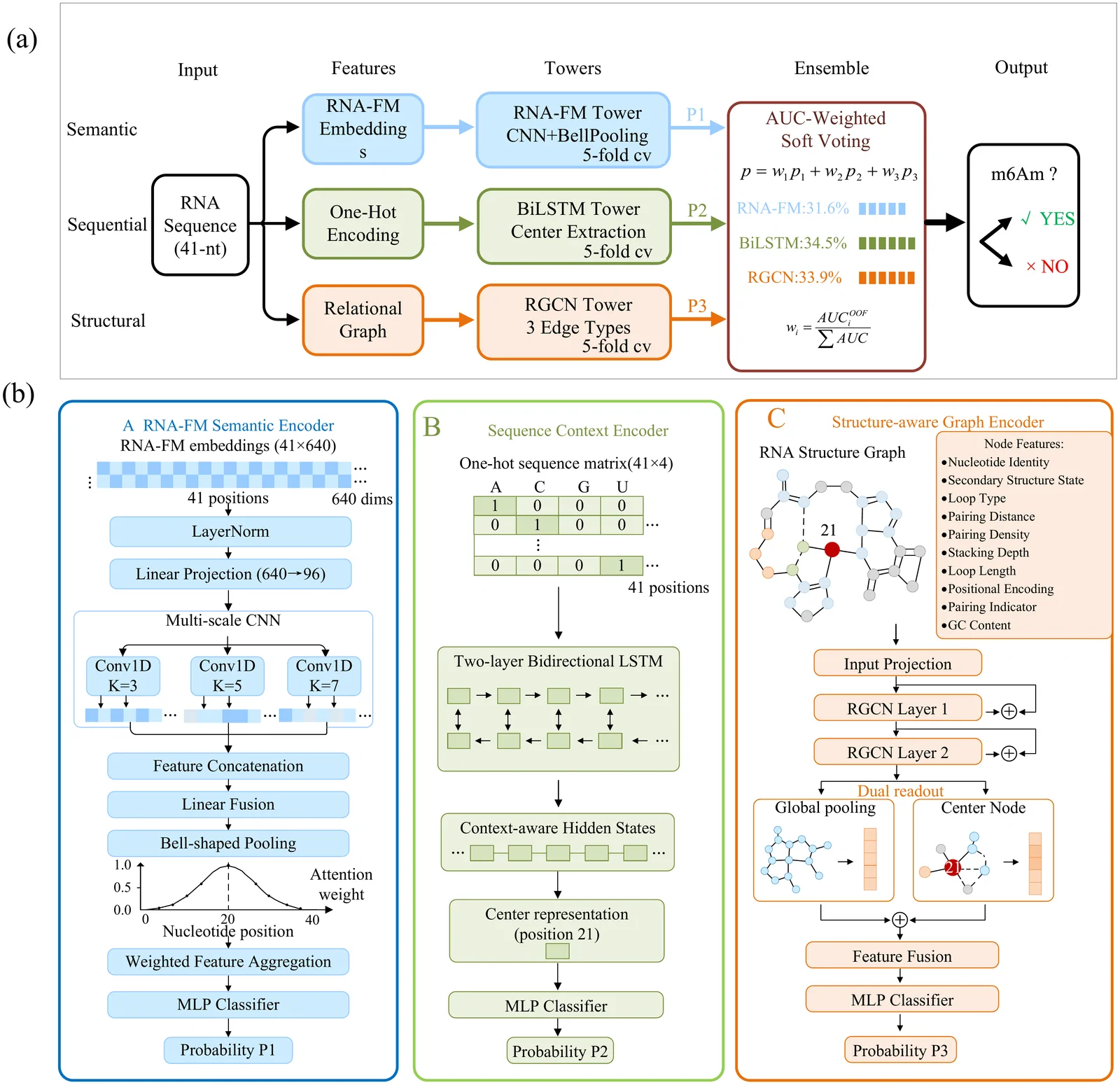
Overview of the TriTower-m6Am framework. **(a)** End-to-end workflow: data preparation, three-branch representation extraction, independent tower training with 5-fold CV, and AUC-weighted ensemble prediction. **(b)** Per-tower architectures, each operating on a distinct representation of the 41-nt input: **(A)** RNA-FM tower with BellPooling; **(B)** One-Hot BiLSTM tower with center extraction at position 20; **(C)** RGCN tower with three relation-specific edge types (backbone, base-pairing, neighborhood).

Fig 1b shows the per-tower encoders. Each tower consumes the same input window but through a different representation, so the three towers answer different questions about the same sequence: the RNA-FM tower asks what semantic patterns the window carries, the One-Hot BiLSTM tower asks how the nucleotide order reads, and the RGCN tower asks how the nucleotides are arranged in 2D space.

Keeping the towers separate during training has three practical benefits: the optimization landscape of each tower stays small, each tower can be ablated cleanly, and each tower is free to specialize in its own representation space without being pulled toward a shared objective.

### RNA-FM semantic tower

The first tower is built on RNA-FM [13], a Transformer-based [19] RNA language model pre-trained on 23.7 million non-coding RNA (ncRNA) sequences. For every 41-nt window, we pre-compute the per-nucleotide embedding from the frozen RNA-FM backbone, which produces a 41×640 matrix, where 640 is the hidden dimension of the RNA-FM backbone; The downstream encoder then squeezes this into a 48-dimensional sequence representation: layer normalization, a linear projection to 96 dimensions, three parallel 1-D convolutions with kernels 3, 5 and 7 whose outputs are concatenated and projected back to 48, BellPooling to reduce the 41 positions to a single vector, and a two-layer classifier. Exact layer sizes and normalisation details are listed in the Supporting Information.

BellPooling is what makes this tower position-aware. The 41 per-position features are summed with learned weights $w_{i}$ produced by


$$\begin{array}{c} w_{i}=softmax({logits}_{i}),\;i=0,1,...,L-1, \end{array}$$ (1)

with *L* = 41. Before training, the logits are filled from a bell-shaped Cauchy-type prior


$$\begin{array}{c} {logits}_{i}=\ln\;\left(\frac{1}{1+{\left|\frac{i-c}{b}\right|}^{\gamma }}\right), \end{array}$$ (2)

centered at *c* = 20 with width *b* = 5 and exponent γ=2. Under softmax, this gives $w_{i}\propto 1/(1+|(i-20)/5{|}^{2})$, i.e., a symmetric bell that peaks at the candidate site and is free to shift during training. The prior encodes a simple expectation (positions next to the cap-proximal adenosine are more informative) which training can then refine.

### One-Hot BiLSTM sequential tower

The second tower drops every external prior and reads the window as a raw 4-dimensional one-hot code (A = [1,0,0,0], U = [0,1,0,0], G = [0,0,1,0], C = [0,0,0,1]), giving a 41×4 input. The encoder is a 2-layer bidirectional LSTM [20] with 64 units per direction (128 total), so each position’s hidden state is informed by nucleotides on both sides. As the representative vector we keep only the hidden state at position 20: the candidate site sits in the middle of the window, and the bidirectional state at that position already aggregates upstream and downstream context. A two-layer classifier Linear(128 → 64) → GELU (Gaussian error linear unit) → Linear(64 → 1) then maps this 128-dim summary to a logit. The design is deliberately low-information on the input side—no pre-training, no structure—so whatever signal this tower recovers must come from nucleotide order itself, which is what we want to measure against the other two towers.

### RGCN graph tower

The third tower turns the secondary structure into a graph and processes it with a Relational Graph Convolutional Network [21]. We chose RGCN over the more common Graph Attention Network (GAT) [22] for one practical reason: GAT shares one attention mechanism across every edge type, so it cannot tell us whether the model is reading the backbone, the base pairs, or the local neighborhood. RGCN, by giving each edge type its own weight matrix, keeps these contributions separate, which is exactly what the later edge-type ablation needs.

### Graph construction

Each RNA sequence is represented as a graph with 41 nodes (one per nucleotide). Three types of directed edges are defined: *backbone* edges connecting adjacent nucleotides (*i*,*i* + 1) (80 per sequence); *base-pairing* edges connecting nucleotides paired in the predicted secondary structure under the standard rules (Watson–Crick A–U and G–C, plus the wobble pair G–U); and *neighborhood* edges connecting nucleotides at distance 2, (*i*,*i* + 2) (78 per sequence).

### Node features

Each node is represented by a 21-dimensional structural feature vector (Table 2).

**Table 2:** Structural node features (21 dimensions).

Feature group	Dim	Description
Nucleotide one-hot	4	A, U, G, C
Secondary structure	3	Paired (, paired), unpaired.
Loop type	6	Hairpin, interior, bulge, multi, external, stem
Pair distance	1	Distance to paired partner (0 if unpaired)
Pair density	1	Number of paired neighbors within window
Stacking depth	1	Normalized consecutive base-pair stacking depth
Loop length	1	Normalized length of containing unpaired region
Position (sin, cos)	2	Sinusoidal positional encoding
Is paired	1	Binary indicator
GC content	1	Local GC content
**Total**	**21**	

### RGCN architecture

The tower consists of four stages: (1) input projection: Linear(21 → 64), followed by LayerNorm, GELU activation, and Dropout(0.3); (2) two consecutive RGCNConv(64, 64, num_relations = 3, num_bases = 3) layers, each augmented by residual connections and LayerNorm; (3) readout: concatenation of global mean pooling and the center node embedding, projected to 64 dimensions via a linear layer; and (4) classification: Linear(64 → 32), GELU activation, and a final Linear(32 → 1). The RGCN uses 3 basis decomposition matrices (num_bases = 3) to control parameter complexity. Edge dropout (rate = 0.1) is applied during training for regularization.

### Implementation

TriTower-m6Am is implemented in Python 3.9 with PyTorch 2.0 [23] and PyTorch Geometric (PyG) [24] for RGCN operations. RNA secondary structure is predicted using RNAfold from the ViennaRNA package 2.0 [18]. RNA-FM embeddings are extracted using the pre-trained model from Chen et al. [13]. All experiments run on a single NVIDIA GPU with 16 GB of memory. The inference-time and memory measurements reported in S1 Table were taken separately, on an NVIDIA RTX 3060 with 12 GB, as noted in that table. The source code is available at https://github.com/xiong-0212/TriTower-m6Am.

### Training and ensemble strategy

#### Loss function and class imbalance.

We use BCEWithLogitsLoss with a positive class weight $\min(n_{neg}/n_{pos},3.0)$ to address the ~10:1 class imbalance. The cap at 3.0 was chosen empirically to prevent positive samples from dominating the loss; higher caps caused training instability and specificity degradation, while this cap maintained a stable sensitivity–specificity balance across all towers. Label smoothing ($y_{smooth}=y\times 0.92+0.04$) softens the target distribution and improves calibration under class imbalance.

#### Optimization.

We use AdamW [25,26] with learning rate $5\times 10^{-4}$ and weight decay $5\times 10^{-3}$. The learning rate follows a linear warmup over the first 10 epochs, then decays via cosine annealing [27]. We clip gradients at a maximum norm of 1.0 and train with automatic mixed precision (AMP) using PyTorch’s GradScaler.

Early stopping uses balanced validation MCC computed on a 1:1 downsampled subset of the validation fold, with patience of 50 epochs. Optimal hyperparameters were identified through a two-phase grid search. Phase 1 screened 144 configurations on a coarse grid. Phase 2 validated the top-5 configurations with full 5-fold cross-validation. The final values are listed in Table 3. The independent test set was never used during hyperparameter search; it was reserved exclusively for final evaluation.

**Table 3:** Optimal hyperparameters identified by two-phase optimization.

Hyperparameter	Search Space	Optimal
Learning rate	{1e-4, 3e-4, 5e-4, 1e-3}	5e-4
Weight decay	{1e-3, 5e-3, 1e-2}	5e-3
Dropout	{0.2, 0.3, 0.4, 0.5}	0.4
EMA (exponential moving average) decay	{0.99, 0.995, 0.999}	0.990
*Fixed training parameters*		
Label smoothing	—	y×0.92+0.04
Positive class weight cap	—	3.0

We apply an exponential moving average (EMA, decay 0.99) of model parameters during training; the smoothed shadow parameters are used for validation and inference.

#### Cross-validation.

Each tower is trained with 5-fold stratified cross-validation (random seed = 123). The split is stratified by class label, which preserves the ~10:1 negative-to-positive ratio within each fold; implementation details are given in the Supporting Information. For each fold, the model is trained on 4/5 of the training set (32,560 samples) and validated on the held-out 1/5 (8,140 samples). The out-of-fold (OOF) predictions are collected for all training samples, and the test set predictions from all 5 folds are averaged. The independent test set (640 samples) is held out from this procedure and used only for final evaluation.

#### AUC-weighted ensemble.

The three towers are ensembled via AUC-weighted voting. The ensemble weight for tower *i* is


$$\begin{array}{c} w_{i}=\frac{{AUC}_{i}^{OOF}}{\sum_{j=1}^{3}{AUC}_{j}^{OOF}}, \end{array}$$ (3)

where ${AUC}_{i}^{OOF}$ is the OOF AUC of tower *i* on the training set. The final ensemble probability is


$$\begin{array}{c} p_{ensemble}=\sum_{i=1}^{3}w_{i}\cdot p_{i}, \end{array}$$ (4)

a standard weighted averaging scheme for classifier ensembles [28,29].

### Evaluation metrics

Performance is evaluated using AUC (Area Under the ROC Curve), MCC (Matthews Correlation Coefficient), SN (Sensitivity/Recall), SP (Specificity), and F1 Score, all computed on the independent test set. The MCC is the standard Matthews correlation coefficient, computed from the confusion matrix at the operating threshold described below. Threshold-dependent metrics (MCC, SN, SP, F1, and accuracy, ACC) are reported at the optimal threshold determined by maximizing balanced validation MCC on OOF training predictions. Concretely, the out-of-fold predictions of the 40,700 training samples are first down-sampled to a class-balanced subset (3,700 positives and 3,700 randomly selected negatives) so that the validation distribution matches the 1:1 class ratio of the independent test set; the threshold maximizing MCC on this balanced subset is then selected once and applied unchanged to the test set. Thresholds are therefore never tuned on the test data. The default threshold of 0.5 is used only when comparing against published methods whose threshold-dependent metrics are reported at 0.5.

## Results

### Overall performance: ROC and precision–recall analysis

Table 4 reports the performance of TriTower-m6Am against DTC-m6Am, three published m6Am predictors, and three deep learning baselines. TriTower-m6Am achieves the highest AUC (0.776), MCC (0.425), and F1 (0.743) on the independent test set.

**Table 4:** Performance comparison on the independent test set (640 samples). Bold marks the best value in each column among the rows evaluated on this test set; the three rows marked with an asterisk are literature values obtained under the original studies’ own evaluation protocols and are not directly comparable, so they are excluded from the bolding. ^*^Results from the DTC-m6Am study [10], evaluated on the same independent test set. Threshold-dependent metrics are reported as described in the Evaluation metrics section: our models use the balanced-OOF threshold, while DTC-m6Am is evaluated at the default threshold of 0.5, with its sensitivity and specificity re-measured from the official checkpoint (SN 0.878, SP 0.503).

Method	AUC	MCC	SN	SP	F1
**TriTower-m6Am (ours)**	**0.776**	**0.425**	0.869	0.531	**0.743**
DTC-m6Am [10]	0.765	0.411	**0.878**	0.503	0.740
m6AmPred^*^ [16]	0.735	0.289	0.887	0.358	0.702
DLm6Am^*^ [30]	0.730	0.322	0.875	0.409	0.710
m6Aminer^*^ [15]	0.754	0.331	0.874	0.420	0.713
CNN baseline	0.765	0.355	0.484	**0.847**	0.592
BiLSTM baseline	0.746	0.342	0.869	0.441	0.716
Transformer baseline	0.724	0.322	0.822	0.481	0.702

Because the two methods differ in their operating point, we compare them at matched specificity rather than at a single arbitrary threshold (Table 10). At every matched specificity in the range SP = 0.40–0.70, TriTower-m6Am recovers slightly more true sites than DTC-m6Am: the difference corresponds to 1–7 additional true positives out of 320 (mean 3.5), which is small and whose bootstrap 95% confidence interval includes zero at every operating point. We therefore report the sensitivity difference as a small, threshold-independent trend. TriTower-m6Am achieved a slightly higher AUC (0.776 vs. 0.765; not statistically significant) and comparable sensitivity (0.869 vs. 0.878), using 4.4 times fewer parameters than DTC-m6Am, with every prediction decomposable into its three tower contributions. Its advantage is therefore competitive performance at substantially lower model size.

We re-evaluated DTC-m6Am from its official checkpoint under identical preprocessing and reproduced its reported AUC = 0.765 and MCC = 0.411, so the comparison is against the actual model rather than literature numbers. At matched specificity, sensitivity is comparable between the two methods (Table 10): both operate at a threshold chosen to favour sensitivity (SN 0.87), and the associated specificity (0.53) reflects this operating point rather than a property of either model—a user can move along the ROC curve to trade sensitivity against specificity as the application requires.

Three standard deep learning baselines — a convolutional neural network (CNN), a BiLSTM, and a Transformer — trained on the same one-hot input with the same hyperparameters all trail the ensemble. The CNN is the strongest of the three (MCC = 0.355), followed by the BiLSTM (0.342) and the Transformer (0.322). We read this as evidence that the lift does not come from picking the right architecture in isolation—it comes from combining heterogeneous representations: none of the three one-hot baselines reaches MCC = 0.40 on its own, whereas the full ensemble reaches 0.43. Even against our strongest single tower (the RGCN graph tower, which also receives structural and relational input and is not one of these baselines) the ensemble’s margin is narrow—AUC 0.776 vs. 0.768; MCC 0.425 vs. 0.405. What the fusion contributes is auditability: the voting weights make each prediction decomposable into its three tower contributions, a property no single-tower model provides.

Fig 2 visualizes the multi-metric comparison. Among the compared methods, TriTower-m6Am achieved the highest AUC, MCC, and F1. Its sensitivity was comparable to, not higher than, that of DTC-m6Am (0.869 vs. 0.878), and it achieved this with roughly one-quarter of the parameters. The radar profile reflects this pattern: top ranking performance with sensitivity on par with the strongest baseline. The margin over the single-representation baselines is what points to complementary rather than redundant towers. The ensemble processes one sample in 12.41 ms with 330K trainable parameters, against 3.6–8.5 ms and 1.45M parameters for DTC-m6Am on the same hardware (S1 Table). Fewer parameters therefore do not translate into lower latency or smaller peak memory here, and the reasons are architectural rather than incidental: the three towers run in series, the RGCN alone accounts for 8.61 ms of the total because typed message passing at batch size 1 leaves the GPU largely idle, and the ensemble’s peak is set by the cuDNN recurrent workspace of its LSTM tower rather than by its weights. The TriTower rows also exclude the two per-window preprocessing steps that deployment requires and DTC-m6Am does not; measured separately, RNAfold adds 2.81 ms per window and a single RNA-FM forward pass at batch size 1 adds 18.33 ms, so the true cost of a new prediction is roughly 34 ms, several times the figure in the table. S1 Table gives the per-component breakdown and the architectural reasons for the difference.

**Fig 2:**
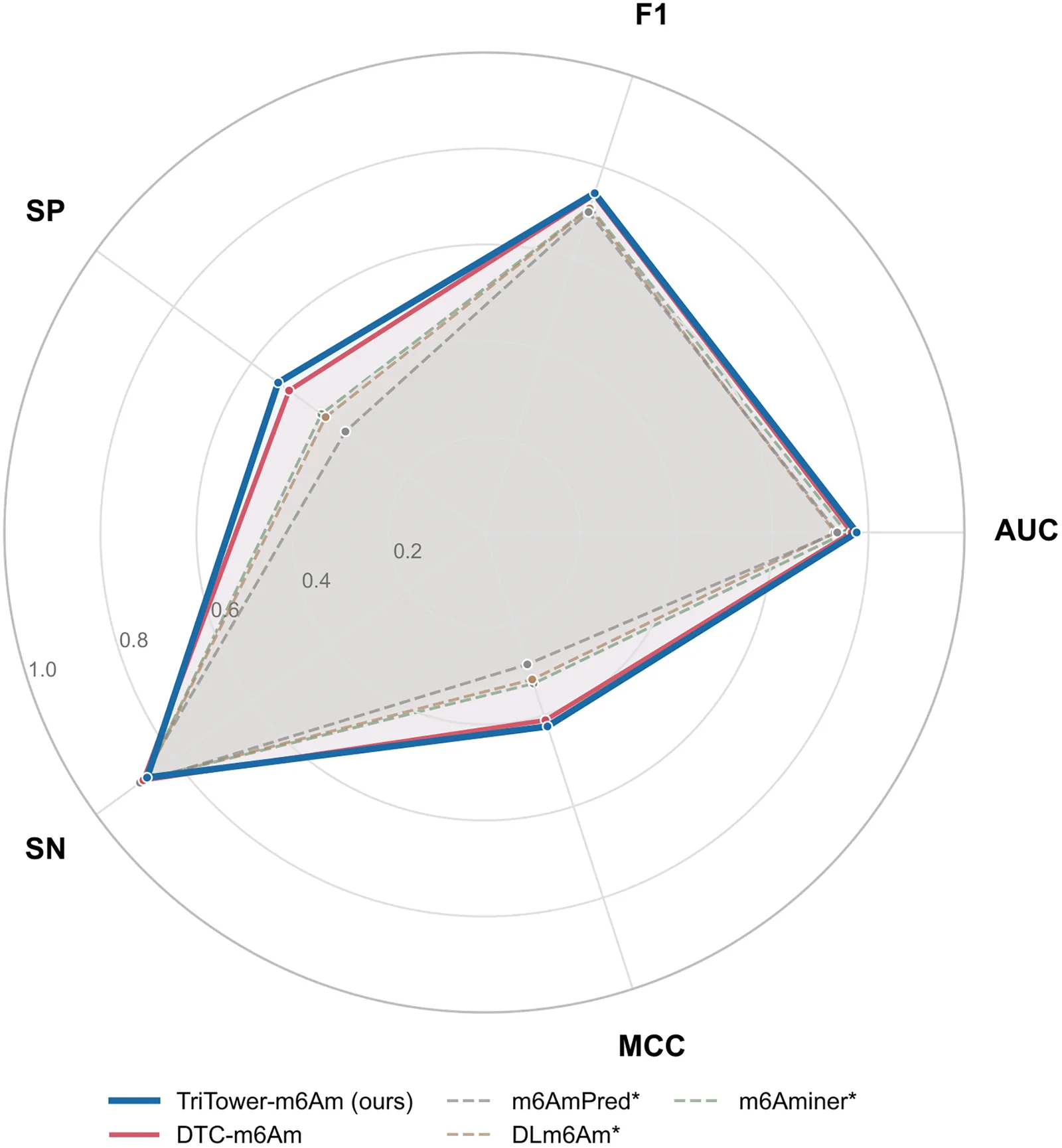
Radar chart comparison of model performance across five metrics. Values closer to the outer circle indicate better performance. The accuracy axis present in the original submission has been removed, so that the radar reports exactly the metrics tabulated in Table 4. Spoke labels have also been removed for legibility; each axis is labelled at the rim. ^*^Results from the DTC-m6Am study [10].

The ROC and precision–recall (PR) curves (Fig 3) show that the ensemble achieves the highest overall AUC (0.776) and AP (0.758; Average Precision, the area under the PR curve). Among the three towers (Table 5), RGCN gives the strongest standalone AUC (0.768), with RNA-FM close behind (0.762) and BiLSTM last (0.756). What matters for ensembling, though, is not the AUC ranking but the shape of each tower’s error. RNA-FM operated conservatively (SN = 0.516, SP = 0.838), One-Hot BiLSTM was recall-oriented (SN = 0.878, SP = 0.419), and RGCN combined high sensitivity with moderate specificity (SN = 0.884, SP = 0.488). This is the configuration ensemble methods are designed for: the towers make mistakes in different directions, so a disagreement between two towers carries information that the third tower can break, and an agreement between any two is usually a correct call. The ensemble converts this into SN = 0.869 with SP = 0.531—retaining the sensitivity of the two recall-oriented towers while raising specificity above either of them.

**Table 5:** Tower ablation study. Checkmarks indicate which towers are included. Left columns ablate one tower from the full ensemble; right columns evaluate each tower in isolation. “w/o” denotes removal of the corresponding tower, and “pp” denotes percentage points.

Configuration	Full ensemble	w/o RNA-FM	w/o One-Hot	w/o RGCN	RNA-FM only	One-Hot only	RGCN only
RNA-FM	✓		✓	✓	✓		
One-Hot BiLSTM	✓	✓		✓		✓	
RGCN	✓	✓	✓				✓
AUC	**0.776**	0.770	0.773	0.769	0.762	0.756	0.768
ΔAUC (pp)	—	-0.51	-0.27	-0.68	-1.33	-1.98	-0.78
MCC	**0.425**	0.414	0.423	0.380	0.373	0.334	0.405
SN	0.869	0.906	0.863	0.863	0.516	0.878	0.884
SP	0.531	0.466	0.538	0.491	0.838	0.419	0.488

**Fig 3:**
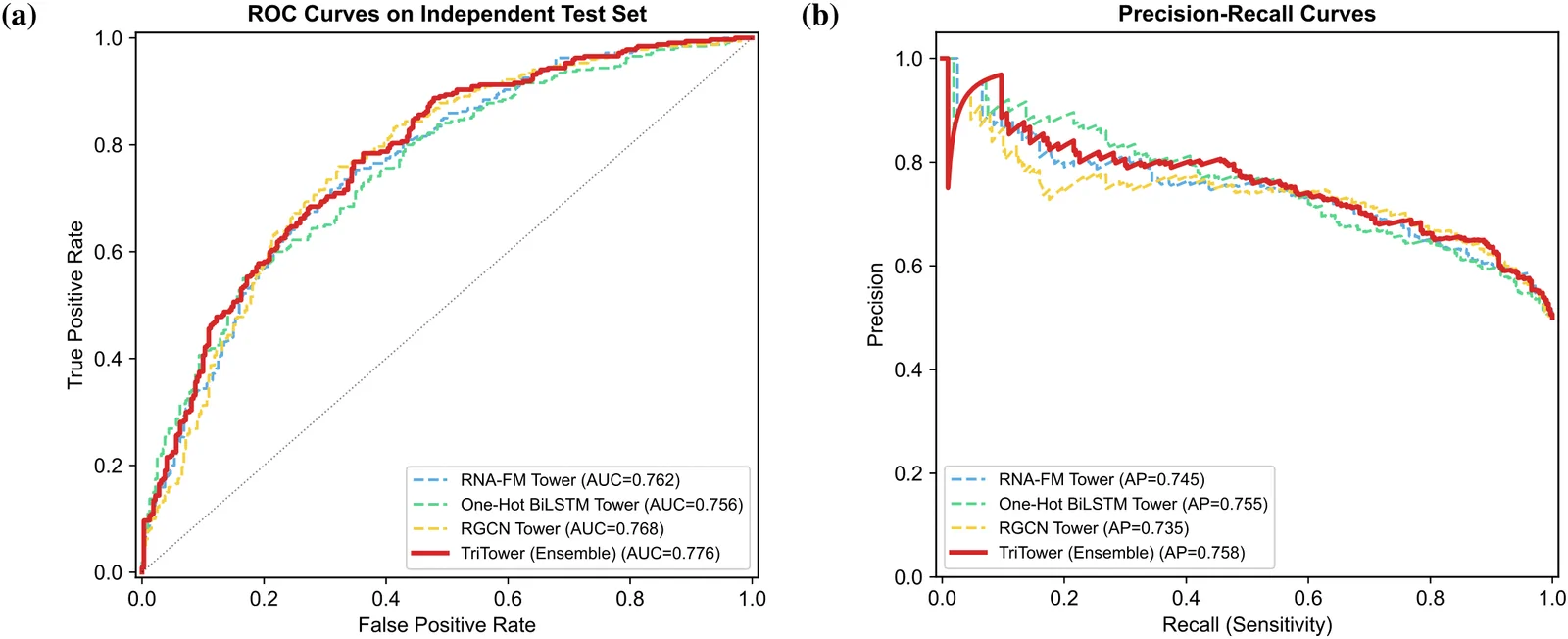
ROC and Precision-Recall curves on the independent test set. **(a)** ROC curves. **(b)** Precision-Recall curves. Solid lines represent the ensemble (red) and individual towers. AUC and AP values are listed in the legends.

### Representation complementarity: ablation and parameter-matched baselines

Overall performance tells us that the ensemble works; it does not tell us why. The ablations in this section pull the model apart at two levels: first at the tower level (which representation matters?) and then at the component level (which design choices inside each tower matter?).

Fig 4 shows the same tower ablation per metric, Table 5 reports the tower ablation. RGCN is the strongest standalone tower (AUC = 0.768, MCC = 0.405) and also the most balanced in its sensitivity–specificity trade-off, a strength that the graph analysis below traces to the relational encoding of local context over sequence-derived node features. RNA-FM matches RGCN in AUC (0.762) but has the highest specificity and lowest sensitivity of the three towers (SN = 0.516, SP = 0.838), indicating conservative decisions. The One-Hot BiLSTM has the lowest AUC (0.756) but a strongly recall-oriented operating point (SN = 0.878, SP = 0.419), concentrating its errors as false positives. These complementary error profiles provide the different decision directions that the ensemble combines.

**Fig 4:**
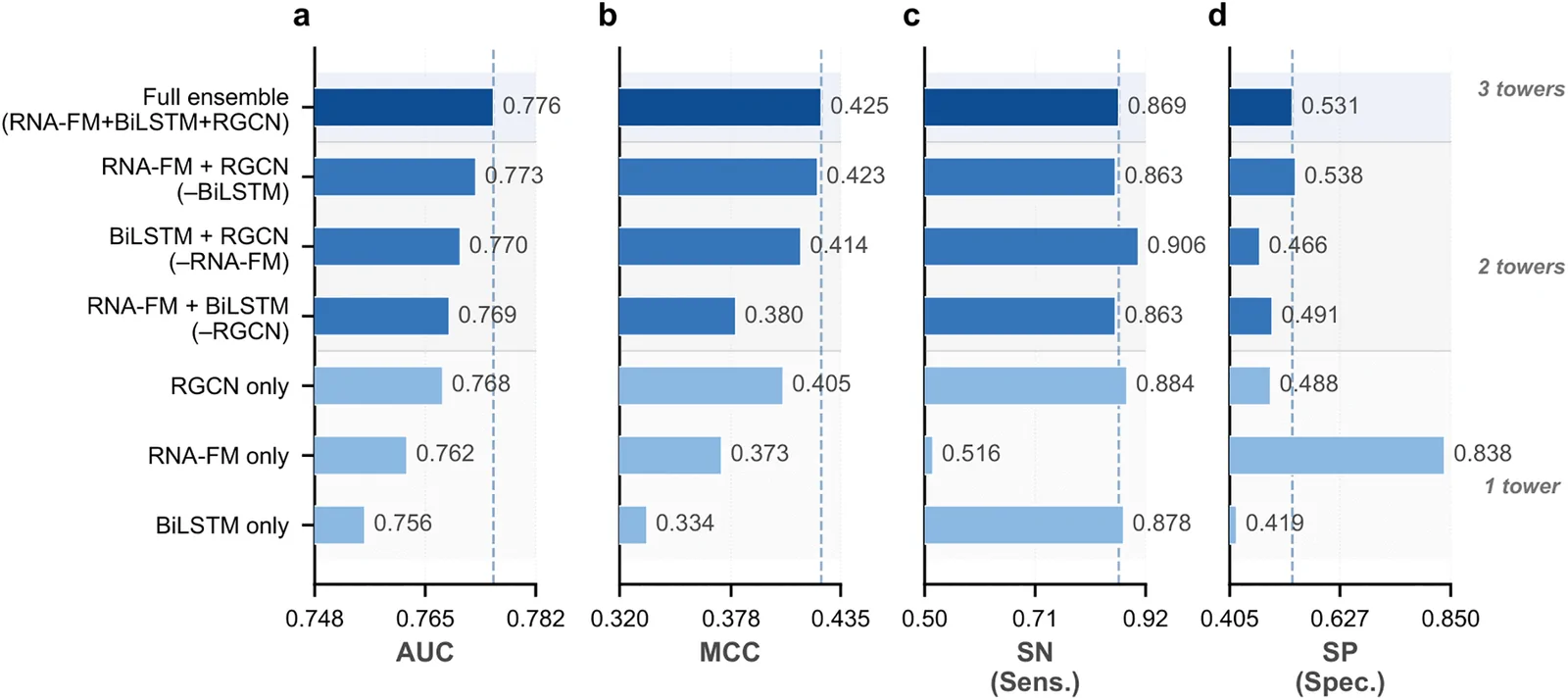
Tower ablation: per-metric comparison. Horizontal bars show AUC, MCC, SN, and SP for each tower configuration. The dashed vertical line marks the full-ensemble value. Configurations are grouped by number of towers (3, 2, 1).

Tower-removal confirms that none of the three is redundant. Pulling the RGCN out of the ensemble costs the most AUC (-0.68 percentage points), in line with its being the strongest single tower; pulling the BiLSTM costs the least (-0.27 percentage points), since its recall-oriented contribution is partly covered by RGCN, which is also sensitivity-oriented. The range is narrow (0.27–0.68 percentage points), which on its own could be read either as “all towers are useful” or as “the remaining two towers compensate.” The CKA (centered kernel alignment) scores in the Discussion settle the question: the three representations are correlated but far from identical (CKA 0.55–0.67), so each tower carries information the other two do not, and the narrow AUC range is a sign of genuine complementarity rather than redundancy.

Fig 5 reports component-level ablations. Removing label smoothing produces the largest AUC decline (-1.06%), confirming its importance for calibrating predictions under severe class imbalance. Replacing BellPooling with mean pooling yields a minimal decline (-0.27%), indicating that the bell-shaped initialization captures most of the benefit with little learned deviation from the prior (Fig 9). Removing EMA slightly improves AUC (+0.15%), which is within noise range, indicating negligible impact on generalization under the current training regime. The corresponding effect on specificity is modest and consistent with the AUC ordering: removing label smoothing leaves specificity essentially unchanged (0.531 vs. 0.528), replacing BellPooling with mean pooling costs sensitivity (SN -0.06) rather than specificity (SP + 0.07), and removing EMA shifts specificity by 0.03, within noise, so no component trades a specificity loss for its AUC gain.

**Fig 5:**
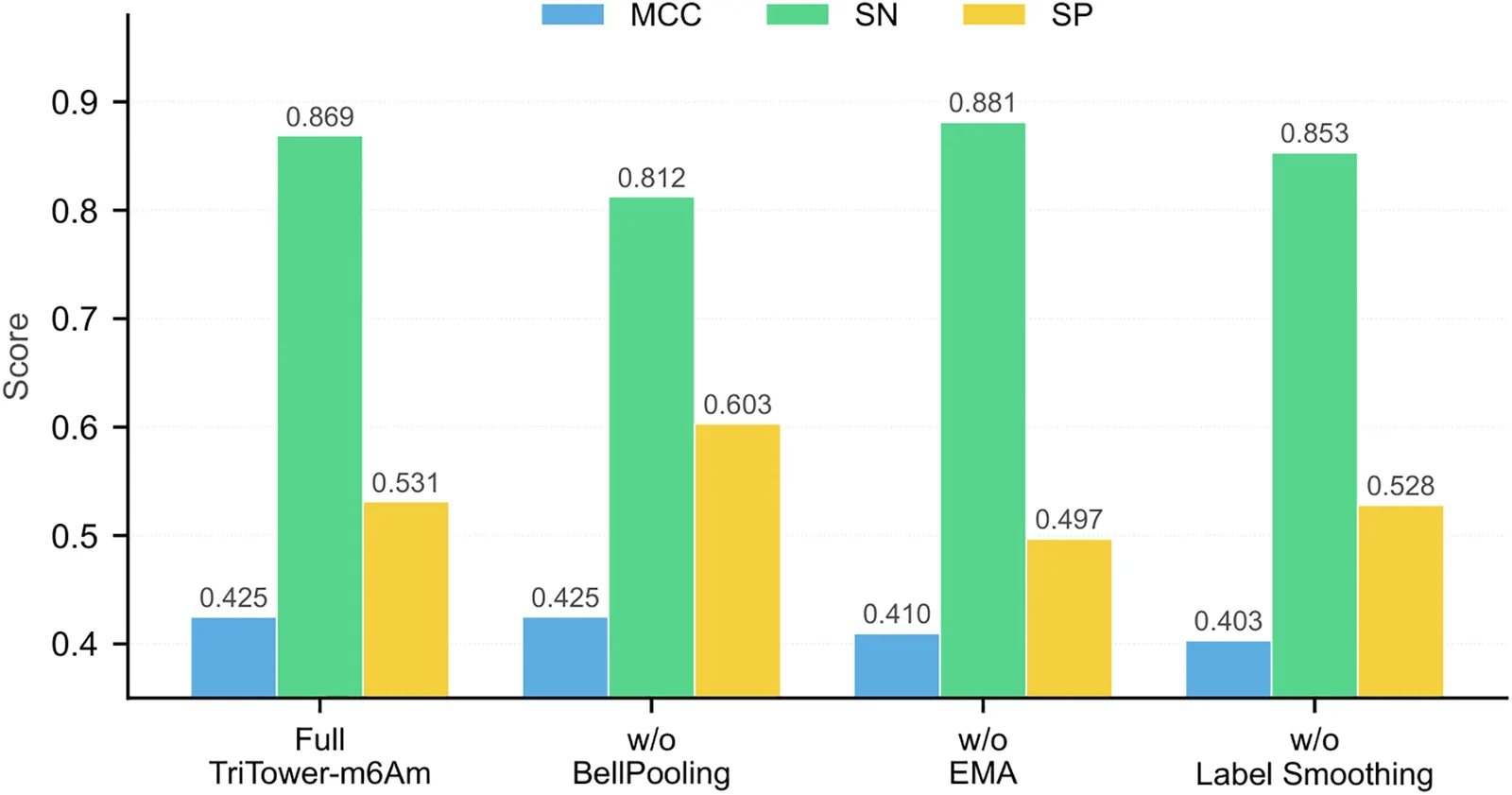
Component ablation study. “w/o” indicates the corresponding module is removed from the full model. AUC is omitted as it varies negligibly across configurations; MCC, SN, and SP reveal the distinct behavioral changes induced by each component.

A natural concern is whether the ensemble’s higher scores simply reflect increased model capacity rather than genuine complementarity among the three representations. To rule out this confound, we trained parameter-matched “wide” single-tower baselines in which each tower’s hidden dimension was enlarged to approximate the total parameter count of the full three-tower ensemble (~330K). Specifically, the wide RNA-FM tower doubles the projection dimension from 96 to 160 and the multi-scale CNN channels from 48 to 80 per branch (yielding a merged width of 240 instead of 144), totaling 318K parameters; the wide BiLSTM tower doubles the hidden dimension from 64 to 128 per direction (256 total), totaling 549K parameters; and the wide RGCN tower triples the hidden dimension from 64 to 192, totaling 381K parameters. Each wide tower matches or exceeds the full ensemble in capacity (Fig 6).

**Fig 6:**
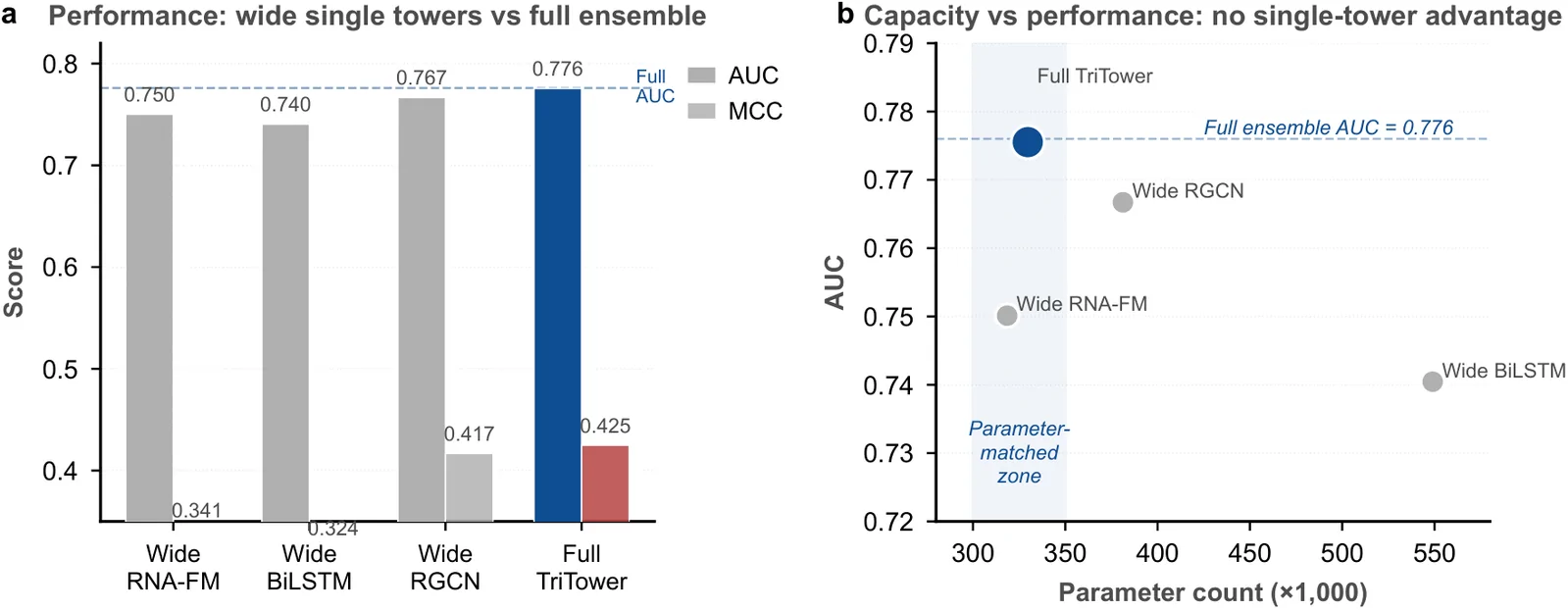
Parameter-matched single-tower baselines vs. full ensemble. **(a)** AUC and MCC on the independent test set. All three wide single towers fall below the full ensemble despite matching or exceeding its parameter count. **(b)** Capacity–performance scatter: increasing parameters alone does not recover ensemble performance. The shaded region marks the parameter-matched zone (~300–350K).

On the independent test set, all three wide single towers underperform the full ensemble (AUC = 0.776, MCC = 0.425): wide RNA-FM reaches AUC = 0.750/MCC = 0.341, wide BiLSTM reaches AUC = 0.740/MCC = 0.324, and wide RGCN reaches AUC = 0.767/MCC = 0.417. The pattern is the same in every case: the wide tower matches or exceeds the full ensemble in parameter count, yet falls short on the test set. Two of the three (wide BiLSTM and wide RGCN) reach out-of-fold AUC above 0.90 on the training data and then drop to 0.74–0.77 on held-out data, which is the classical overfitting signature—extra capacity memorizes the training folds instead of learning a more general function. We read this as the clearest evidence in the paper that the ensemble’s gain is not a capacity effect: widening a single tower is strictly worse than splitting the same parameter budget across three heterogeneous representations, even though the latter option is shallower per representation.

### Graph mechanisms: edge type and node feature ablation

A key advantage of the RGCN’s relation-specific design is that it enables direct assessment of which graph relationships matter most. Fig 7 and Table 6 report two complementary sets of experiments: removing one edge type at a time from the full graph (columns 2–4), and using a single edge type in isolation (columns 5–7).

**Table 6:** Edge type ablation for the RGCN tower. The first column lists the three edge types (backbone, pairing, neighbor); a checkmark indicates that the edge type is included in the corresponding model configuration.

Edge type	Full	w/o neighbor	w/o pairing	w/o backbone	Backbone only	Pairing only	Neighbor only
Backbone	✓	✓	✓		✓		
Pairing	✓	✓		✓		✓	
Neighbor	✓		✓	✓			✓
AUC	0.768	0.757	0.766	0.739	0.757	0.701	0.738
MCC	0.405	0.414	0.418	0.374	0.399	0.326	0.366
SN	0.884	0.828	0.866	0.806	0.813	0.834	0.822
SP	0.488	0.572	0.528	0.556	0.575	0.469	0.528

**Fig 7:**
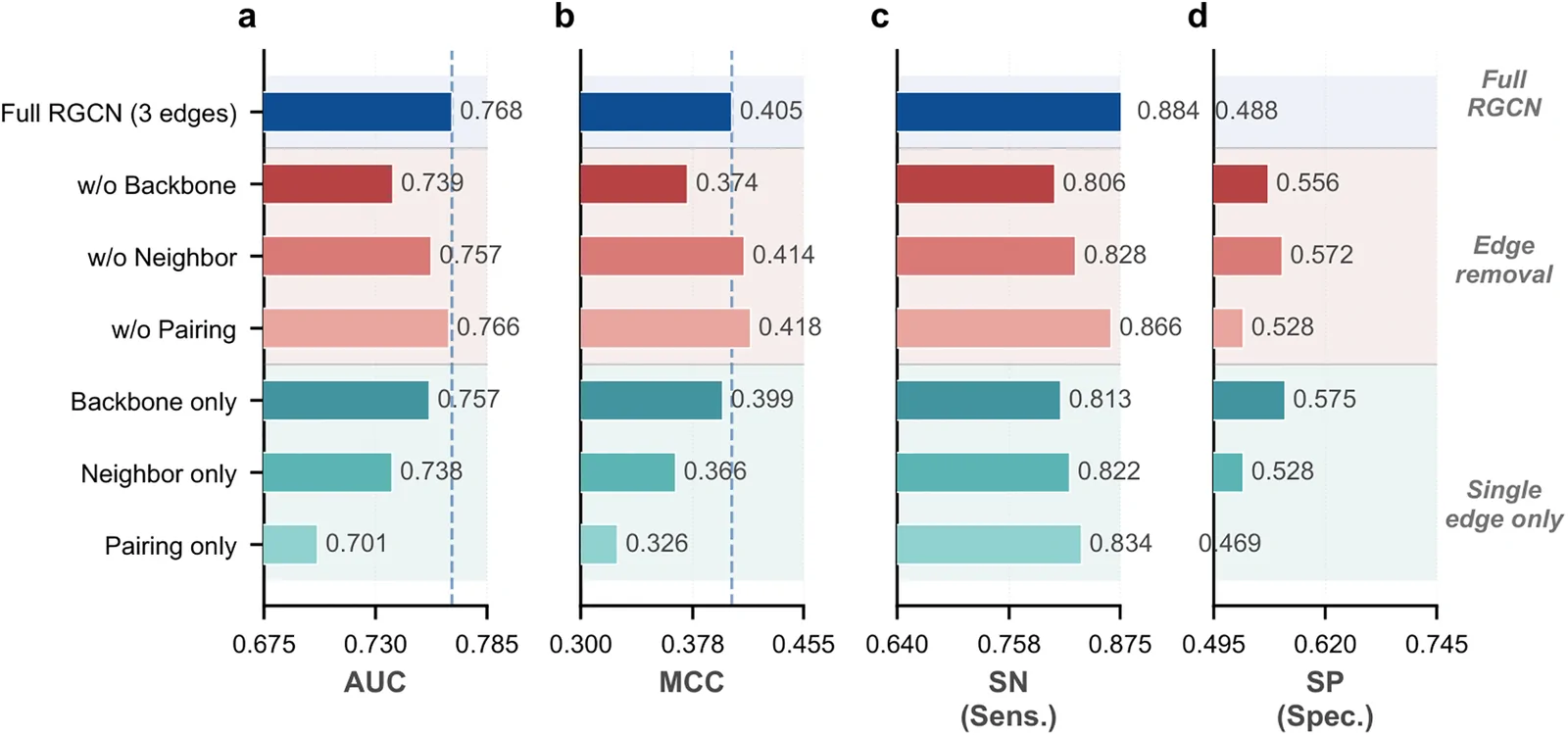
Edge-type ablation: per-metric comparison. Top group: full RGCN and removal of each edge type (red). Bottom group: single-edge-type-only models (teal). The dashed vertical line marks the full three-edge RGCN performance.

The graph signal is concentrated in the backbone. Pulling backbone edges out of the full graph costs 2.91 percentage points of AUC (0.768 → 0.739) and 0.031 MCC, the largest single-edge drop in the table. Running the RGCN on backbone edges alone, with both pairing and neighbor edges deleted, still reaches AUC = 0.757 and MCC = 0.399—within 0.011 AUC and 0.006 MCC of the full three-edge model. In other words, almost everything the RGCN learns about m6Am can be learned from linear adjacency alone.

Pairing edges contribute the least. Dropping them from the full graph shifts AUC by only 0.002 (0.768 → 0.766), and a model that uses pairing edges in isolation is the weakest single-edge configuration (AUC = 0.701, MCC = 0.326). Neighbor edges sit in between: removing them costs 0.010 AUC, and neighbor-only reaches AUC = 0.738 with a sensitivity-leaning profile (SN = 0.822, SP = 0.528), i.e., it is willing to call positives but cannot reliably reject negatives.

Taken together, the three edge types form a clear hierarchy (backbone ≫ neighbor > pairing). This ordering is only visible because RGCN keeps edge types separate; a GAT with shared attention would average the three contributions and report a single combined effect, which is precisely the information loss that motivated the RGCN choice.

The edge-type ablation showed that pairing edges contributed negligibly to performance, which suggests that whatever graph signal the tower uses might reside in the node features rather than in the edges. To test this, we performed a leave-one-group-out (LOGO) ablation on the 21-dimensional RGCN node feature vector, which comprises eight biologically motivated feature groups (Table 7). Each group was removed in turn while retaining the remaining seven groups, and the RGCN tower was retrained under identical settings (5-fold cross-validation, seed 123).

**Table 7:** Composition of the eight LOGO ablation groups for the 21-dimensional RGCN node feature vector. Each group was removed in turn while retaining the remaining seven groups. Feature indices refer to columns in the 21-dimensional node feature vector defined in Table 2.

Group	Dim	Feature group	Features (indices)	Description
1	4	Nucleotide one-hot	A, U, G, C (0–3)	Nucleotide identity at each position
2	3	SS state	Open (, Close), Unpaired. (4–6)	Dot-bracket state from RNAfold
3	6	Loop type	Paired, Hairpin, Internal, Bulge, Multi, Exterior (7–12)	Local loop classification
4	3	Pairing geometry	Pair dist., Pair density, Is paired (13, 14, 19)	Base-pair geometry summary
5	1	Stacking depth	Stacking depth (15)	Normalized consecutive base-pair stacking
6	1	Loop length	Norm. loop length (16)	Length of containing unpaired region
7	2	Positional encoding	sin, cos (17–18)	Sinusoidal positional encoding
8	1	GC content	GC content (20)	Local GC ratio in sequence window
	**21**	**Total**	**21 features (indices 0–20)**	

Fig 8 reports the results. The full 21-feature model achieved AUC = 0.764 and MCC = 0.420, consistent with the standalone RGCN tower performance reported in Table 6. Two feature groups dominated performance: removing nucleotide one-hot caused the largest drop (AUC: 0.764 → 0.698, ΔAUC = -0.067; MCC: 0.420 → 0.301, ΔMCC = -0.118), confirming that sequence identity remains the primary signal for m6Am recognition. Positional encoding was the second most important group (AUC: 0.764 → 0.724, ΔAUC = -0.040), reflecting the RGCN’s reliance on position information to locate the central modification site (position 20).

**Fig 8:**
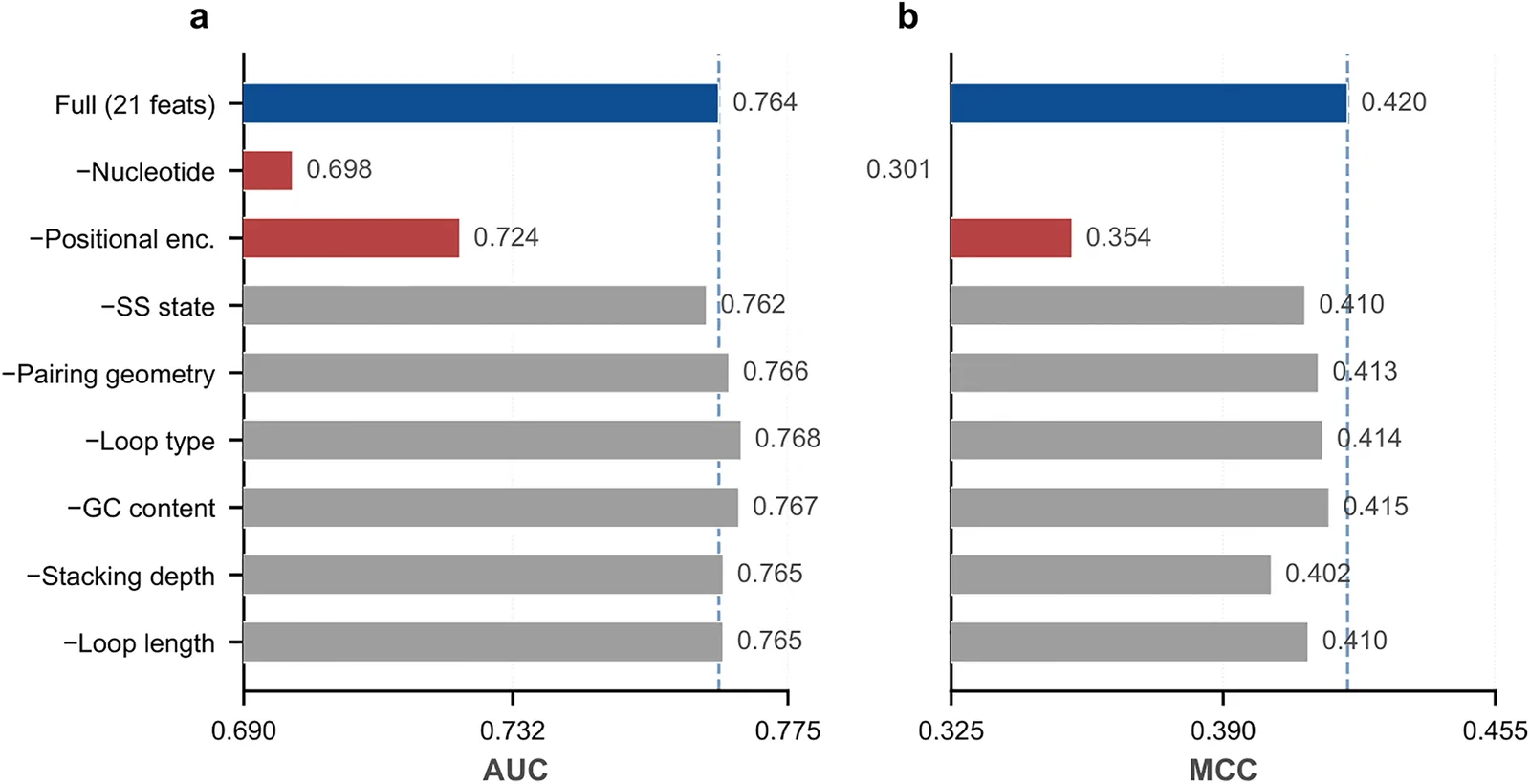
Node feature ablation for the RGCN tower. Leave-one-group-out ablation on the 21-dimensional node feature vector. The dashed line marks the full-model performance. Nucleotide one-hot and positional encoding are the two critical groups (red), while SS state and pairing geometry (whose information is in large part recoverable from the sequence-derived embeddings) show little individual impact (grey).

Removing the SS state or pairing geometry features changed performance by less than 0.002 in AUC (SS state: ΔAUC = -0.002; pairing geometry: ΔAUC = +0.002), and the remaining feature groups (loop type, GC content, stacking depth, loop length) moved AUC by less than 0.004. Read together with the edge-type ablation, these results indicate that the structure-annotation content of the node features is in large part already carried elsewhere in the input: the probing analysis in the Discussion shows that per-base base-pairing status is recoverable from the pre-trained RNA-FM embeddings, so the RGCN receives the fold annotation substantially redundantly with the sequence-derived representation. We therefore tested the correspondence between each window and its predicted structure directly rather than continuing to ablate it.

### Interpretability: BellPooling, integrated gradients, and ensemble weights

The RNA-FM tower employs BellPooling to aggregate RNA-FM semantic features across the 41 positions into a single representation. Fig 9 compares the initialized and learned BellPooling weights. The initialized weights follow a symmetric bell curve centered at position 20 (the m6Am site), encoding the prior that positions near the modification site carry the most relevant semantic information. After training, the weights remain peaked near the center but develop a slight asymmetry, with positions 18–19 receiving marginally increased weight relative to the symmetric prior.

**Fig 9:**
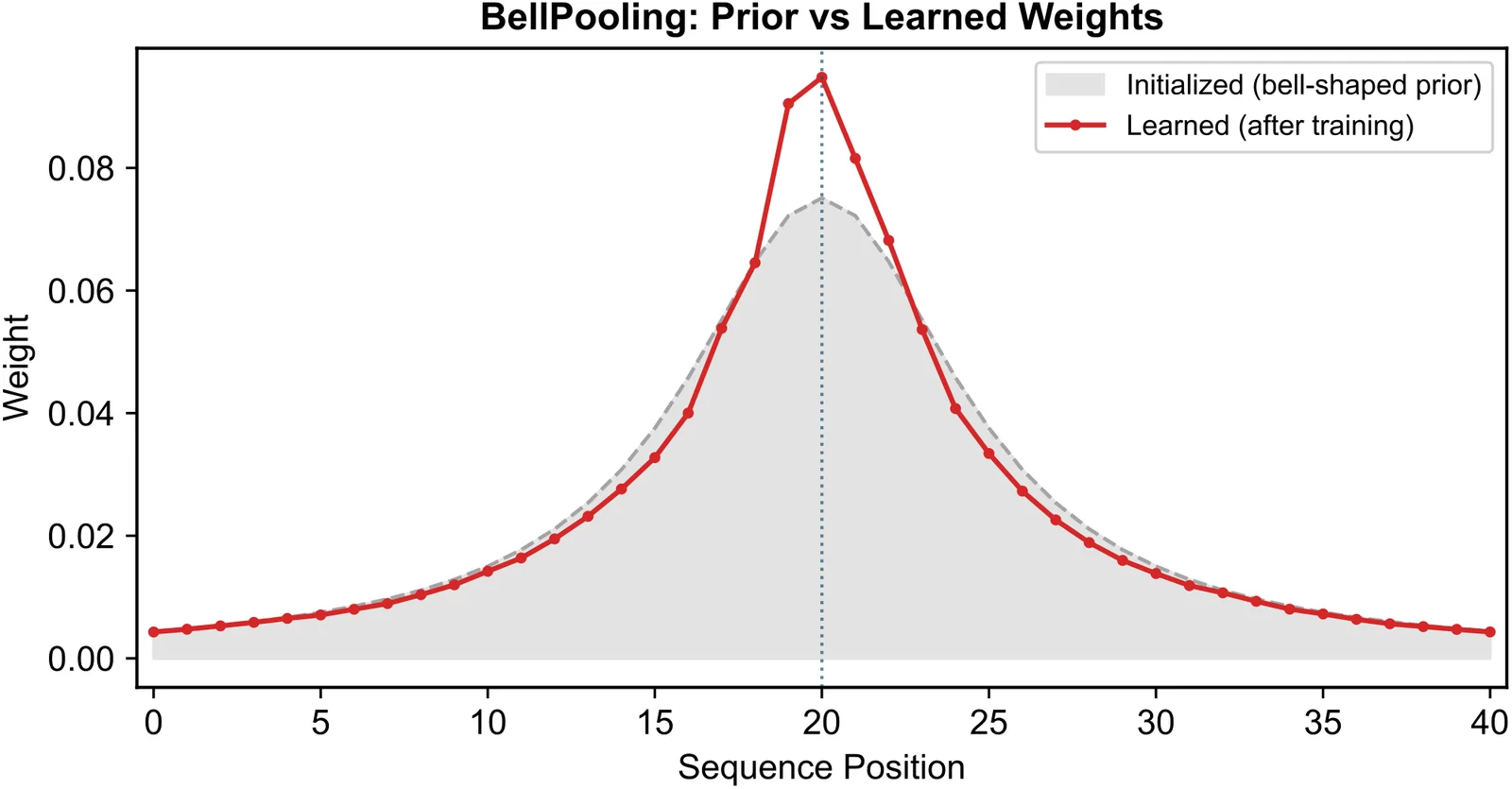
BellPooling weights: initialized (gray) vs. learned after training (red). The vertical dotted line marks position 20.

An important caveat: BellPooling operates on the 48-dimensional CNN-compressed RNA-FM features, not on the raw nucleotide sequence. The learned weights therefore reflect the model’s aggregation preference and should not be interpreted as direct sequence-level attribution. The stability of the center-peaked pattern after training indicates that the bell-shaped prior biases aggregation toward cap-proximal positions while permitting limited adaptation. In the ablation study (Fig 5), replacing BellPooling with uniform average pooling yielded a negligible AUC change (-0.27%), consistent with the observation that the learned weights deviated only slightly from initialization.

While BellPooling reveals the RNA-FM tower’s aggregation preference, it does not provide nucleotide-level attribution. To examine which positions the One-Hot BiLSTM tower relies on for its decisions, we applied Captum’s Integrated Gradients (IG) [31] (Captum v0.8.0) to the one-hot input of the BiLSTM tower. IG assigns each position an attribution score proportional to its contribution to the model’s output relative to a zero baseline (baselines = torch.zeros_like(input), 50 integration steps). The sequence logos in Fig 10 were rendered with logomaker v0.8.7. Dropout layers in the BiLSTM were disabled during attribution to ensure deterministic gradients, while the model was kept in training mode to satisfy cuDNN’s LSTM backward-pass requirement.

**Fig 10:**
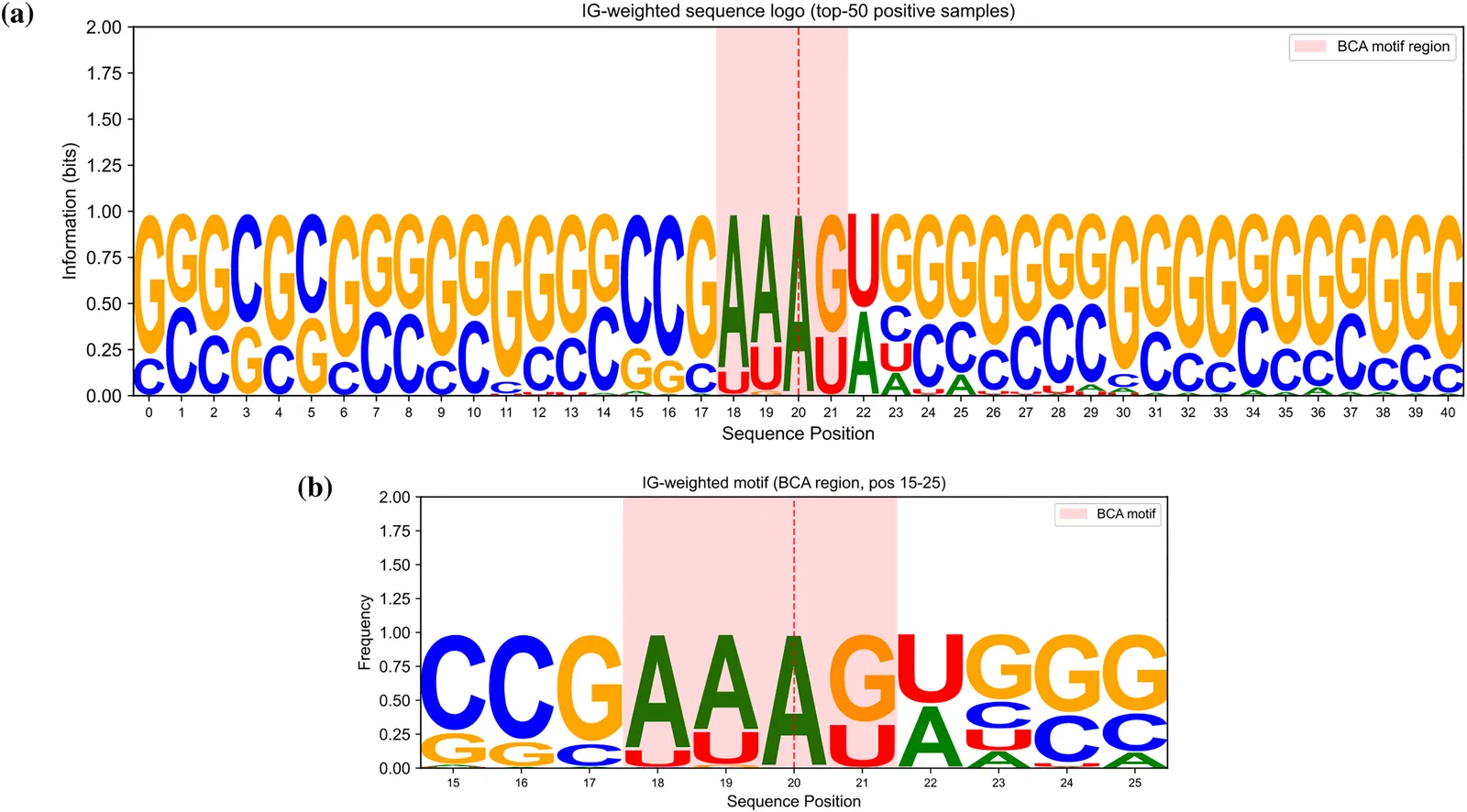
IG-weighted sequence logo from the One-Hot BiLSTM tower. **(a)** IG-weighted sequence logo (top-50 confident positives, 41-nt full length); the shaded region marks the BCA motif (positions 18–21) and the dashed line marks the modification site (position 20). **(b)** Zoomed BCA region (positions 15–25). Letter heights reflect IG-attribution-weighted nucleotide frequencies across the top-50 highest-confidence positive samples, not raw sequence frequencies. The peak attribution at position 22 indicates that the model’s discriminative signal lies downstream of the fixed BCA motif, consistent with PCIF1’s context-dependent methylation efficiency.

To extract a model-attribution-driven motif rather than a simple frequency logo, we selected the top-50 highest-confidence positive samples (ensemble probability |0.5| largest) and built a position weight matrix in which each position’s nucleotide contribution is weighted by its positive-part IG attribution. The resulting 41-nt logo (Fig 10) reveals two findings. First, the total IG weight concentrates downstream of the BCA motif: the peak attribution falls at position 22 rather than at the modification site itself (position 20), and the BCA region (positions 18–21) accounts for 21.3% of the total weight. Second, position 20 is fixed to adenosine by dataset construction in all samples, so the logo at this position is non-discriminative by design; the discriminative signal instead emerges in the flanking context, particularly the + 2 downstream position. This pattern is biologically consistent with PCIF1’s substrate recognition: the enzyme binds the BCA motif [3] but its methylation efficiency is modulated by the local sequence context immediately downstream of the cap-proximal adenosine.

If the three towers encode genuinely complementary information, they should each contribute meaningfully: no single tower should dominate. AUC-weighted ensemble voting makes this directly testable, since each weight quantitatively measures a tower’s discriminative power on the training distribution.

The learned weights are RNA-FM (31.6%), One-Hot BiLSTM (34.5%), and RGCN (33.9%) (Fig 11). This near-uniform distribution indicates that all three towers carry independent predictive value; no single tower dominates the ensemble.

**Fig 11:**
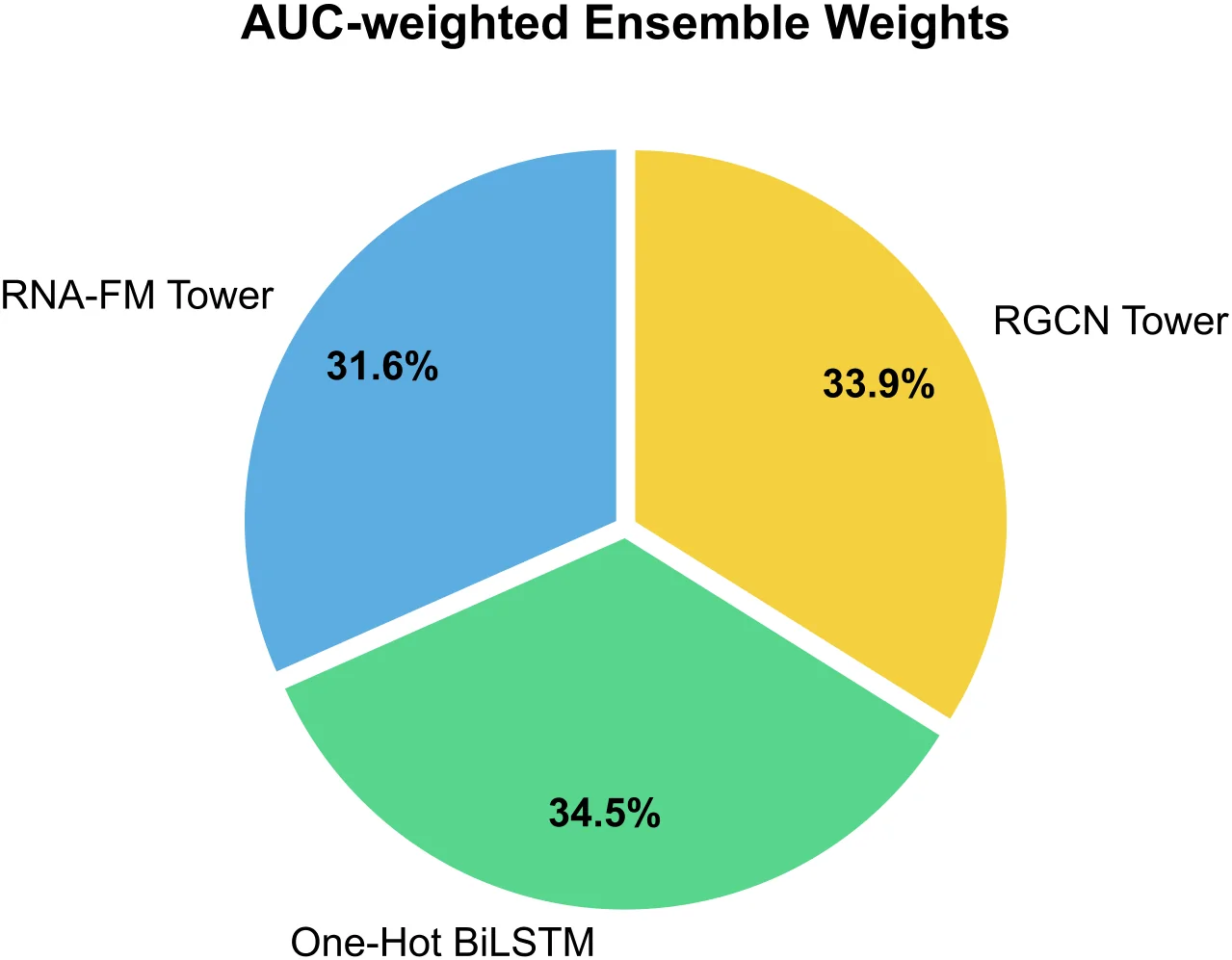
AUC-weighted ensemble weights for RNA-FM (31.6%), One-Hot BiLSTM (34.5%), and RGCN (33.9%).

Unlike black-box strategies such as stacking with a meta-learner, these weights provide an auditable decision trace. For any individual prediction, one can inspect how much each tower contributed to the final probability. This transparency is rare in deep learning ensembles and makes TriTower-m6Am more interpretable in practice.

### Robustness and generalization

To verify that TriTower-m6Am’s performance is not an artifact of a particular random seed, we retrained the full pipeline with three independent seeds (123, 42, 52) using identical hyperparameters and 5-fold cross-validation. The results (Table 8) demonstrate strong stability in ranking quality: AUC varies by only ±0.003 (coefficient of variation = 0.42%) and MCC by ±0.013 (CV = 3.00%). All three seeds exceed DTC-m6Am in AUC (0.765), and in MCC two of the three exceed DTC-m6Am’s 0.411 while the third (0.402) comes within 0.009 of it; with a cross-seed MCC spread of only 0.023, the ranking metrics are stable across initializations and the operating-point metrics vary only mildly (Table 8). The sensitivity and specificity also remain stable across seeds (SN: 0.841 ± 0.036, SP: 0.558 ± 0.032), confirming that the model’s decision behavior is robust to initialization randomness. All threshold-dependent metrics in this table are computed at a threshold selected on balanced out-of-fold predictions for each seed separately, using the same procedure as the main results.

**Table 8:** Multi-seed stability analysis. Results from three independent seeds (123, 42, 52), each with 5-fold cross-validation. All threshold-dependent metrics are computed at a threshold selected on balanced out-of-fold predictions for each seed separately, using the same procedure as the main results.

Seed	AUC	MCC	ACC	F1	SN	SP
123	0.776	0.425	0.700	0.743	0.869	0.531
42	0.776	0.423	0.702	0.741	0.853	0.550
52	0.770	0.402	0.697	0.725	0.800	0.594
**Mean ± Std**	**0.774 ± 0.003**	**0.417 ± 0.012**	**0.699 ± 0.002**	**0.736 ± 0.010**	**0.841 ± 0.036**	**0.558 ± 0.032**
CV (%)	0.42	3.00	0.29	1.36	4.28	5.73

We first evaluated how TriTower-m6Am responds to varying training data availability by sub-sampling negatives at five ratios (1:1, 3:1, 5:1, 7:1, 10:1), keeping all 3,700 positives fixed. The independent test set (640 samples, balanced) remained unchanged. Results are reported in Table 9. Because sub-sampling negatives changes both the class balance and the total dataset size, this experiment measures sensitivity to *data availability* rather than class balance alone.

**Table 9:** Performance under varying training data availability. Higher ratios include more negative samples and larger total datasets. Each ratio was trained independently from a fresh 80/20 stratified split; threshold-dependent metrics are computed at a threshold selected on a class-balanced subset of that split’s validation predictions, never on the test set. The experiment was re-run for this revision so that the same threshold protocol applies throughout.

Ratio	*N*_train_	AUC	MCC	SN	SP	F1	ACC
1:1	7,400	0.741	0.337	0.863	0.444	0.713	0.653
3:1	14,800	0.741	0.345	0.841	0.481	0.713	0.661
5:1	22,200	0.763	0.351	0.916	0.381	0.723	0.648
7:1	29,600	0.759	0.382	0.875	0.475	0.729	0.675
10:1	40,700	0.774	0.404	0.866	0.512	0.736	0.689

Performance trends upward with dataset size, and the 10:1 ratio (the full training set) yields the best results (AUC = 0.774, MCC = 0.404). MCC increases monotonically with the size of the negative pool, from 0.337 at 1:1 to 0.404 at 10:1. AUC shows a shallow non-monotonicity, dipping at 7:1 (0.759) relative to 5:1 (0.763), which may reflect subsampling variance. The 1:1 ratio performs similarly to 3:1 despite better class balance, indicating that the smaller negative pool (3,700 vs. 11,100 negatives) limits performance more than the balance advantage helps.

To isolate the effect of class reweighting from dataset size, we performed a controlled experiment in which all 40,700 training samples were used for every condition and only the positive class weight (pos_weight) in BCEWithLogitsLoss was varied (Fig 12).

**Fig 12:**
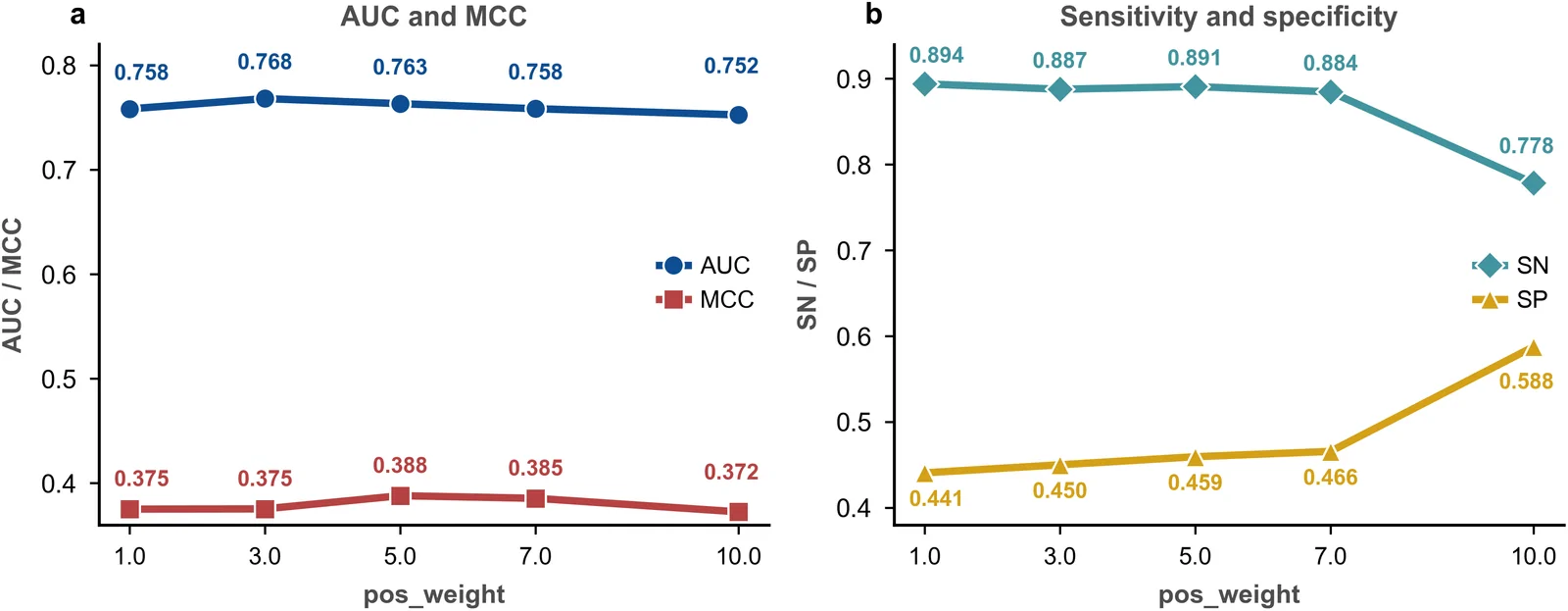
pos_weight sensitivity with fixed dataset size. All 40,700 training samples used; only pos_weight varies. (a) AUC and MCC. (b) Sensitivity and specificity. Each condition was trained independently and its threshold selected on a class-balanced subset of that condition’s validation predictions, under the same protocol used elsewhere in this study; the experiment was re-run for this revision so that the operating points are comparable across conditions.

Under fixed dataset size (all 40,700 training samples), AUC was largely insensitive to pos_weight. Across the five tested values (1.0, 3.0, 5.0, 7.0, 10.0), AUC ranged from 0.752 to 0.768 (Δ = 0.016) and peaked at pos_weight = 3.0, which is the default cap used in the main experiments. MCC peaked at pos_weight = 5.0 (0.388), 0.013 above its value at the default. Sensitivity and specificity shifted only mildly across the sweep: sensitivity remained near 0.89 for pos_weight up to 7.0 and fell to 0.778 at 10.0, while specificity rose from 0.441 to 0.588. The narrow AUC range across a 10 × change in pos_weight contrast with the shifts in sensitivity and specificity indicates that the reweighting strategy primarily tunes the sensitivity–specificity balance rather than overall discriminative power.

Taken together, the two experiments show that (i) TriTower-m6Am benefits primarily from larger and more diverse training sets rather than from a specific class-balance configuration, and (ii) the default pos_weight cap of 3.0 is itself the AUC-optimal setting in this sweep, with MCC maximised at 5.0 but differing from the default by only 0.013. The model is robust to dataset-specific variation in negative-to-positive ratios within the range we tested, requiring no dataset-specific threshold tuning.

The balanced 1:1 test set (320 positive, 320 negative) does not reflect the extreme rarity of m6Am sites in genome-wide screening, where the ratio of true m6Am sites to candidate adenosine positions can reach 1:100 or higher. To evaluate model performance under realistic deployment conditions, we constructed an independent set of negative samples from the GENCODE v44 human reference transcriptome.

**External negative construction.** We scanned all GENCODE v44 transcripts for the BCA motif (B = C/G/U), the canonical cap-proximal sequence context recognized by PCIF1 [3]. After removing known m6Am positive sites and deduplication, 8.6 million candidate BCA positions were identified; 50,000 were randomly sampled for evaluation. Each position was extracted as a 41-nt window (±20 nt flanking), and RNAfold was used to predict secondary structure. These BCA-derived negatives are entirely independent of both the training and test sets used in the main experiments. Note that the sampling is not stratified by transcript expression level or transcript length: because BCA motifs are scanned across the full transcript body, longer transcripts contribute proportionally more candidates, and no expression-weighting is applied. This sampling design matches the deployment scenario of a transcriptome-wide screen, where every candidate adenosine must be evaluated regardless of the source transcript’s abundance.

**Evaluation protocol.** We combined the 320 original test positives with varying numbers of BCA negatives to construct three test sets at imbalance ratios of 1:1 (320 pos + 320 neg), 1:10 (320 pos + 3,200 neg), and 1:100 (320 pos + 32,000 neg). The trained 5-fold ensemble was applied without any retraining and without any threshold adjustment: all model weights were held fixed, and the decision threshold was held at the balanced out-of-fold value of 0.485 used throughout this study. No external data were used for parameter updates or for threshold selection. Because the model predictions were regenerated for this experiment and the negatives at 1:1 are GENCODE-derived rather than the benchmark’s original negatives, the exact SN/SP at 1:1 (0.878/0.528) differ at the second decimal from the balanced test-set evaluation (0.869/0.531; Table 4) even though the AUC coincides (0.776); we report the measured values throughout. We report AUC, average precision (AP), MCC, F1, sensitivity (SN), and specificity (SP) (Fig 13).

**Fig 13:**
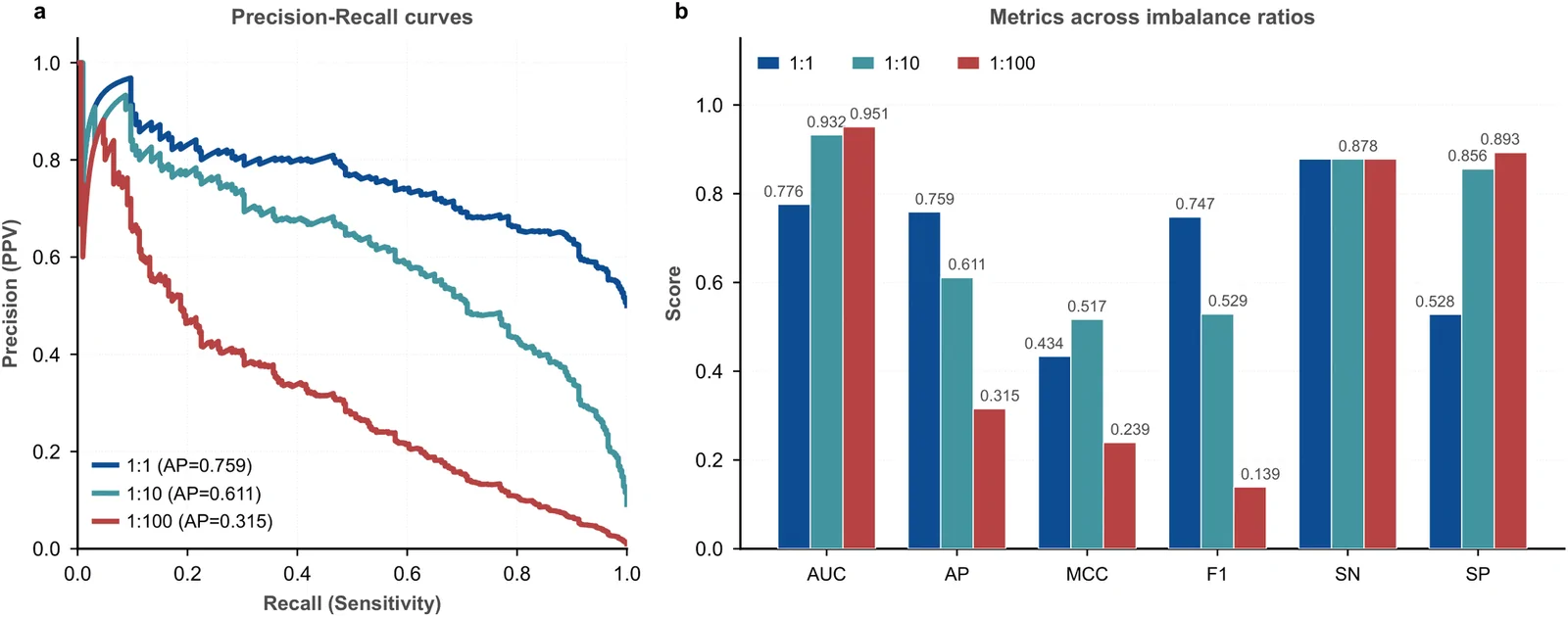
Performance under varying class imbalance ratios. **(a)** Precision-recall curves at 1:1, 1:10, and 1:100 ratios using external BCA-derived negatives from the GENCODE transcriptome. **(b)** Metrics comparison across the three ratios. AUC and SP increase with imbalance due to the inclusion of easier BCA negatives, while AP decreases as the positive class becomes rarer; SN is unchanged across the three ratios, because the decision threshold is held fixed.

**Results.** AUC increased from 0.776 (1:1) to 0.932 (1:10) to 0.951 (1:100), reflecting the inclusion of BCA-derived negatives that the model can distinguish more easily than the original test negatives. In contrast, average precision decreased sharply from 0.759 (1:1) to 0.611 (1:10) to 0.315 (1:100), indicating that in genome-wide screening scenarios, the model produces a substantial number of false positives relative to true positives. Because the threshold is held fixed, sensitivity is constant at 0.878 across the three ratios, while specificity rises from 0.528 to 0.856 to 0.893 as the negative pool becomes progressively easier to reject. MCC peaks at the 1:10 ratio (0.517) and falls to 0.239 at 1:100. The 1:1 entry re-uses the same 320 positives at the same fixed threshold and reaches essentially the same AUC (0.776); its SN/SP (0.878/0.528) are the values measured in this experiment and differ at the second decimal from the balanced evaluation in Table 4 (0.869/0.531), as noted in the evaluation protocol above. We note that if the operating point were instead re-tuned for each ratio, the MCC-optimal threshold would shift upward (0.48, 0.67 and 0.81 at 1:1, 1:10 and 1:100 respectively); we report the fixed-threshold values here so that the operating point is not tuned on the evaluation data, and we return to the practical implication below.

**Interpretation.** The divergence between AUC and AP under extreme imbalance is expected and informative. AUC, which aggregates over all thresholds, benefits from the model’s ability to rank the easier BCA negatives below true positives. AP, which focuses on the precision of the top-ranked predictions, reveals the practical challenge: at 1:100, even a specificity of 0.893 leaves 3,435 false positives among the 32,000 negatives, far exceeding the 320 true positives. This confirms that genome-wide m6Am screening requires complementary filtering (e.g., cap-proximal annotation, transcript start site databases) to reduce the candidate space before model-based prediction. The trained model generalizes to external data without retraining, and the MCC-optimal threshold adapts to the operating ratio, but users should adjust the decision threshold based on the target prevalence in their application. Practical mitigations for the AP drop under extreme imbalance include hard-negative mining to retrain on the highest-scoring false positives, cost-sensitive learning with explicit false-positive penalties, and posterior threshold calibration against an estimate of the operating-ratio prevalence. These directions are complementary to the cap-proximal pre-filtering noted above and are left for future work.

**Composition offset.** Positive and negative windows differ systematically in base composition: mean GC content is 0.625 for positives versus 0.470 for negatives in the training set (0.638 versus 0.552 on the test set), and a position-independent dinucleotide-composition model already reaches AUC = 0.689 on the test set. To bound how much of the benchmark’s discrimination is compositional rather than position-specific, we recomputed the ensemble’s performance within strata of equal GC content: the ensemble’s AUC falls from 0.776 to 0.719 (DTC-m6Am from 0.765 to 0.708), so most of the signal is position-specific, although a measurable part of the apparent discriminative power is attributable to the composition offset. The offset is a property of the published benchmark—m6Am-bearing transcripts initiate predominantly at GC- and CpG-rich promoters—and is shared identically by every method trained on it; the external evaluation against BCA-derived negatives described above was designed as the control for exactly this concern.

### Case studies: high-confidence predictions

To illustrate the ensemble’s behavior in practice, we examined the 10 most confident positive predictions (highest ensemble probability) and the 10 most confident negative predictions (lowest ensemble probability) on the independent test set (Figs 14 and 15). Fig 14 shows the ensemble-probability distribution for the two groups, with the median and mean of each class marked and the 0.5 decision threshold indicated.

**Fig 14:**
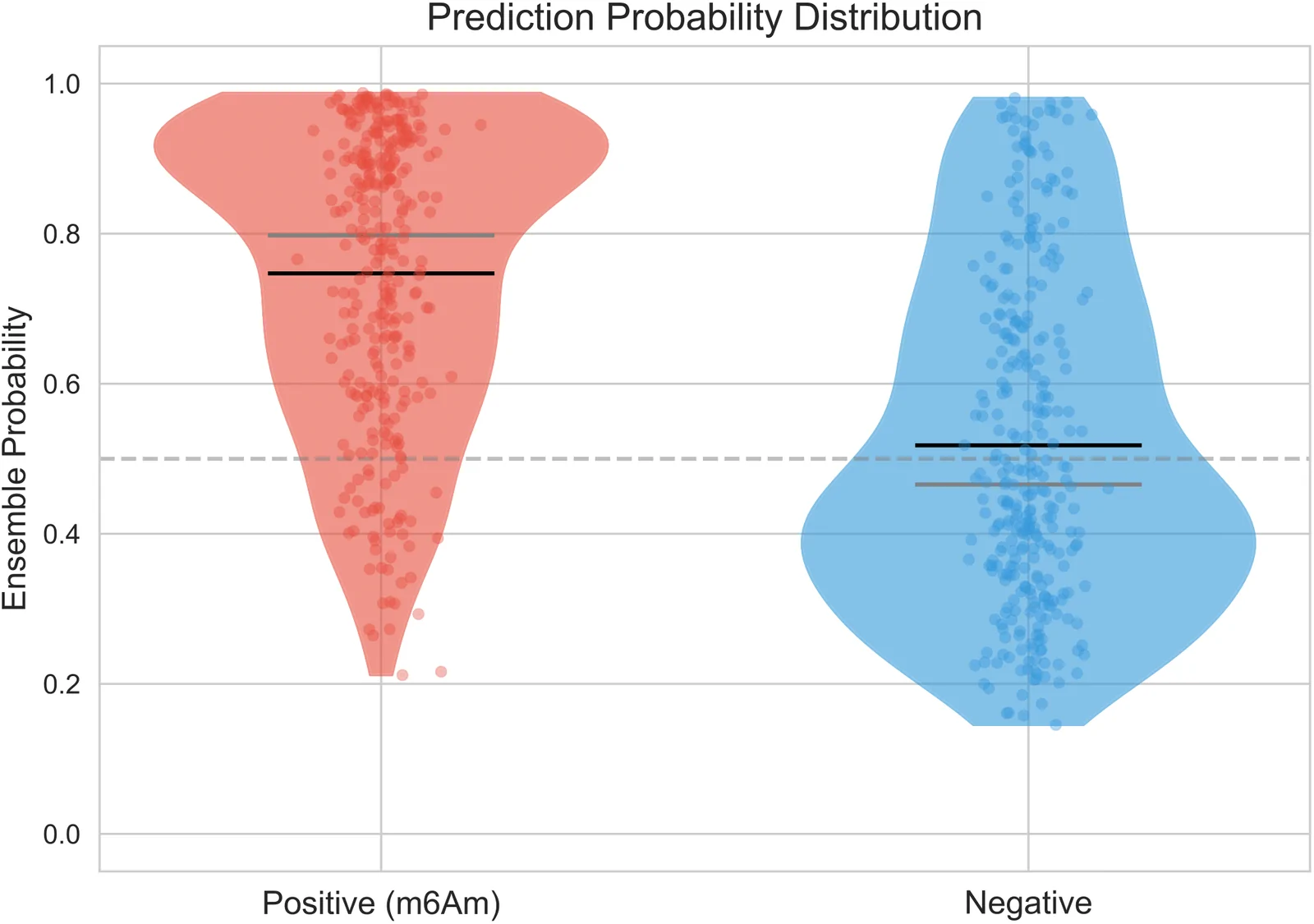
Distribution of ensemble probabilities on the independent test set (320 positive and 320 negative windows). Each violin shows the kernel density of the ensemble probability for one prediction class, overlaid with the individual predictions as dots (red: positive windows, blue: negative windows). Within each violin, the thick black horizontal bar marks the median and the thinner grey bar the mean of that class. The grey dashed horizontal line marks the decision threshold of 0.5. The top-10 positive and top-10 negative predictions discussed in the text are detailed in Fig 15.

**Fig 15:**
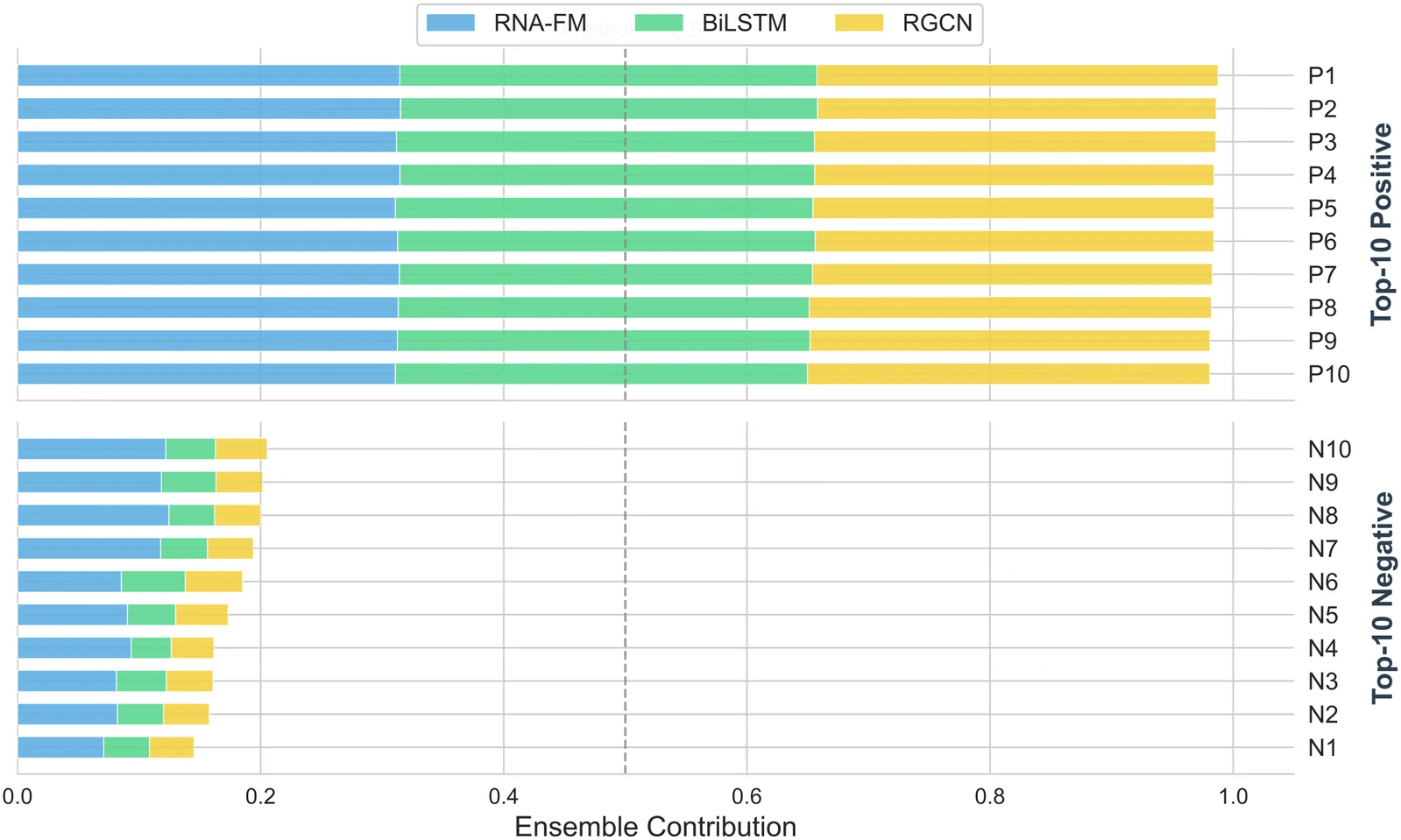
Tower contributions for top-10 positive and top-10 negative predictions.

High-confidence predictions require consensus across towers, since each tower captures a distinct signal. The top-10 positive predictions illustrate this: all three towers agree with high individual probabilities (RNA-FM: 0.98–0.99, One-Hot BiLSTM: 0.98–0.99, RGCN: 0.97–0.98), yielding a precision of 90% (9 true positives, 1 false positive). No single tower dominates these predictions.

Among the top-10 negative predictions, all 10 are true negatives (ensemble probabilities 0.15–0.21). Here the towers exhibit an asymmetric confidence pattern: the One-Hot BiLSTM and RGCN towers are highly confident (probabilities 0.09–0.15), while the RNA-FM tower is markedly less certain (probabilities 0.22–0.39). Despite this asymmetry, the AUC-weighted ensemble correctly classifies all 10 samples as negative, indicating that the voting scheme is robust to individual tower uncertainty: the two confident towers override the RNA-FM tower’s relatively higher probabilities, preventing false positives in the high-confidence negative regime.

### Matched-specificity comparison and uncertainty analysis

Because single-threshold comparisons depend on the operating point, we additionally compared the methods at matched specificity (Table 10 and Fig 16), which is independent of any threshold-selection procedure.

**Table 10:** Sensitivity at matched specificity on the independent test set (640 samples; 320 positives, 320 negatives). This comparison is independent of any threshold-selection procedure: for each target specificity the sensitivity is read at the closest attainable operating point. The final row gives the number of additional true sites recovered by TriTower-m6Am relative to DTC-m6Am; the difference is small and its bootstrap 95% confidence interval includes zero at every operating point (Fig 16).

Method	AUC	SN@SP=0.40	SN@SP=0.45	SN@SP=0.50	SN@SP=0.55	SN@SP=0.60	SN@SP=0.70
**TriTower-m6Am (ours)**	**0.7755**	0.912	0.909	0.891	0.847	0.787	0.694
DTC-m6Am	0.7650	0.909	0.897	0.884	0.828	0.784	0.672
RGCN tower (single)	0.7677	0.919	0.900	0.878	0.844	0.803	0.716
CNN baseline	0.7654	0.922	0.900	0.819	0.800	0.781	0.697
ΔTP (ours - DTC)	—	+1	+4	+2	+6	+1	+7

**Fig 16:**
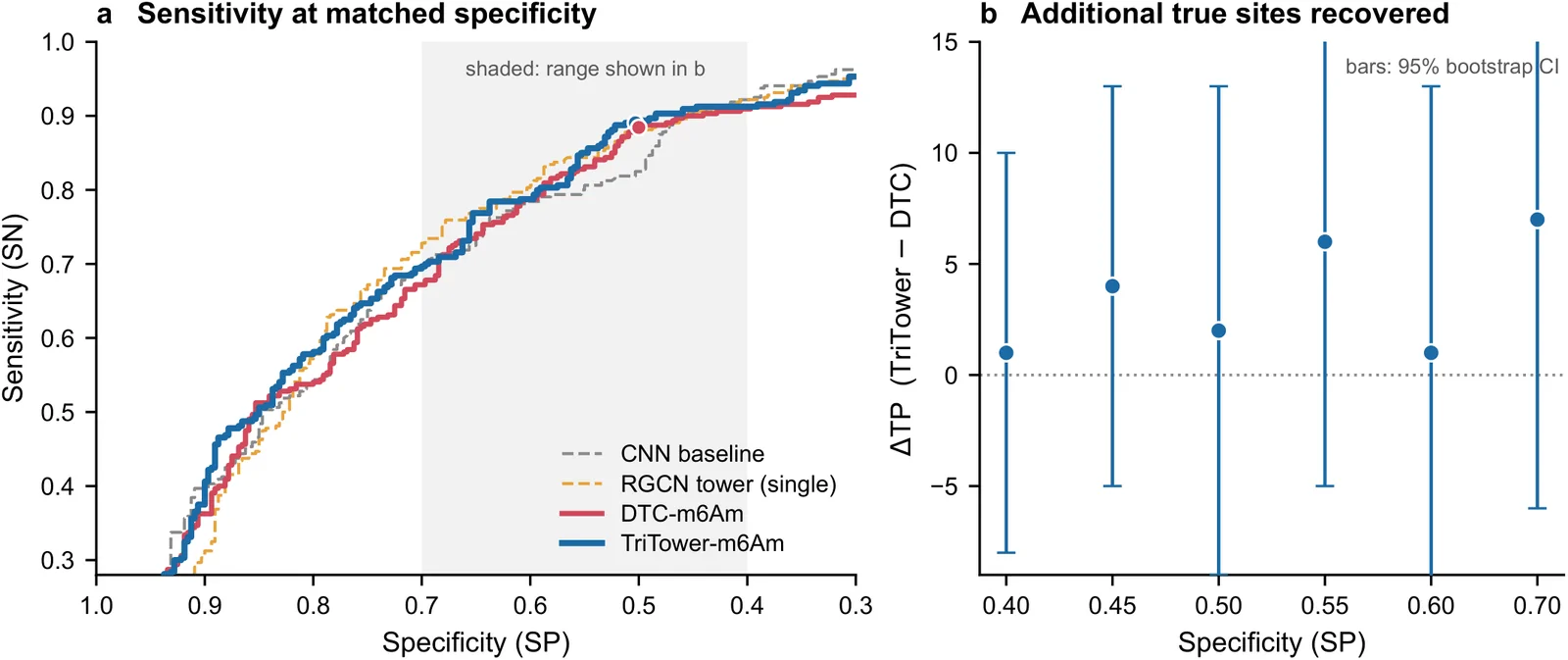
Sensitivity at matched specificity, and additional true sites recovered. **(a)** Sensitivity as a function of specificity for the ensemble, its strongest single tower, DTC-m6Am, and the CNN baseline. Circles mark SP = 0.5; the shaded band is the range expanded in **(b)**. **(b)** Number of additional true positives recovered by TriTower-m6Am relative to DTC-m6Am at each target specificity, with 95% bootstrap confidence intervals. The intervals include zero throughout, so the sensitivity difference is a consistent, threshold-independent trend.

## Discussion

### From single representation to representation complementarity

The complementarity among semantic, sequential, and structural representations explains the performance gain over single-representation predictors. We did not assume these channels were complementary; we designed the architecture so the question could be tested. Pairwise CKA analysis gives a partial answer: RNA-FM vs. One-Hot BiLSTM = 0.547, RNA-FM vs. RGCN = 0.655, and One-Hot BiLSTM vs. RGCN = 0.668 (Fig 17). Read in isolation, a CKA near 0.5–0.7 might look like moderate overlap, but in the context of the tower-removal ablation the number is informative rather than reassuring: pulling any single tower out of the ensemble costs AUC, and the CKA scores are high enough that the remaining towers compensate partially but low enough that they cannot compensate fully. The ensemble gain is therefore genuine complementarity, not a capacity artifact—a reading that the wide single-tower baselines confirm independently (see the wide-baseline analysis above). The BellPooling mechanism, which applies a center-focused positional prior to the RNA-FM tower output, performs essentially the same as uniform pooling (-0.27% AUC; Fig 5) while keeping the aggregation explicitly interpretable: the learned weights deviate only slightly from the bell prior, which we read as the model confirming rather than overriding the biological expectation that cap-proximal positions matter most. We retain BellPooling for this interpretability rather than for a performance margin.

**Fig 17:**
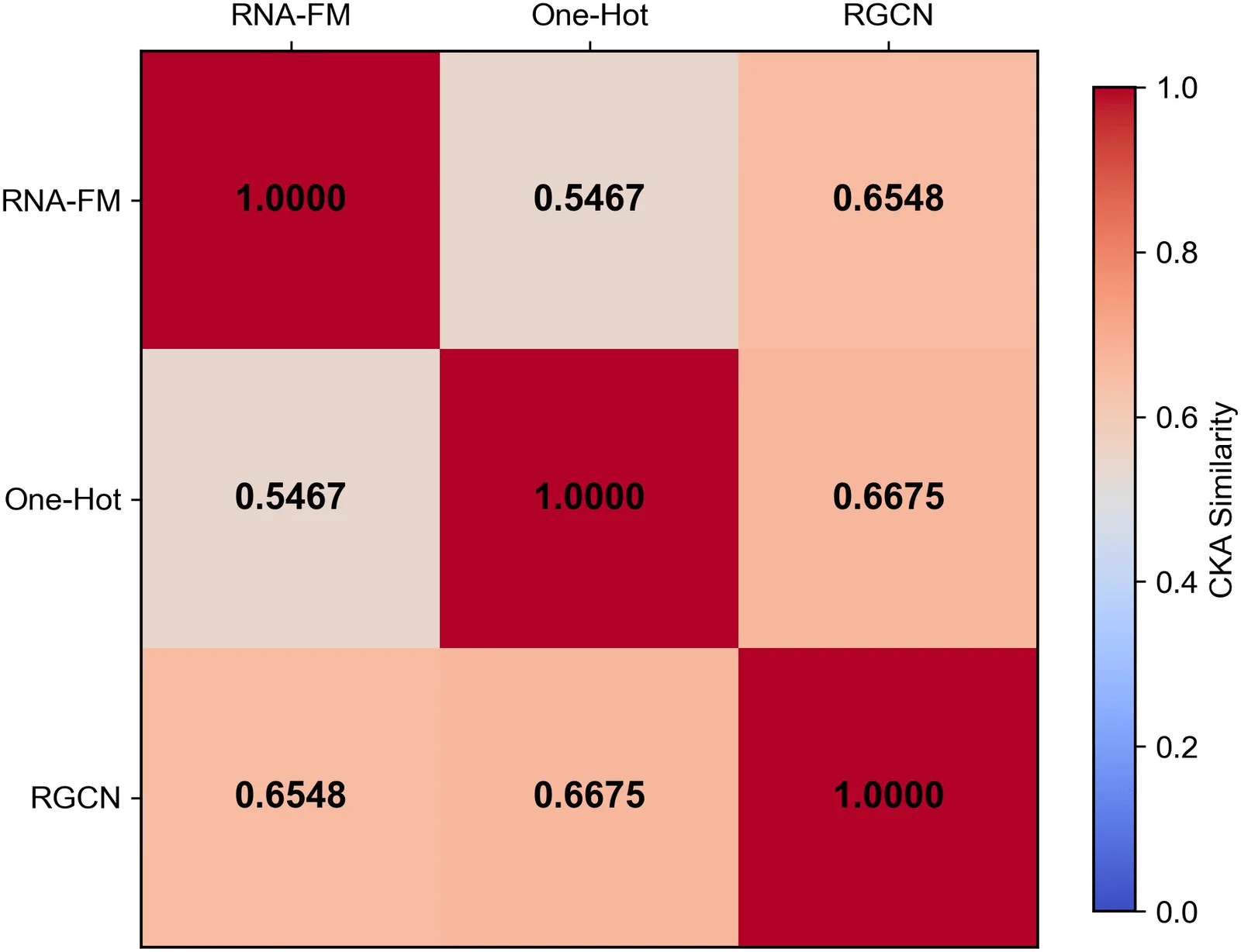
Pairwise CKA similarity between tower representations.

Positive and negative windows differ systematically in base composition (mean GC 0.625 vs. 0.470 in training), and a single global GC value discriminates the classes at AUC = 0.676. Adjusting for GC content within strata reduces the ensemble’s AUC from 0.776 to 0.719, so a substantial part of the signal is position-specific, but part of the discrimination available in this benchmark is compositional rather than local. This bounds the biological reading of what the model rewards: part of the task is distinguishing GC-rich from GC-poor cap-proximal context, which is correlated with, but not identical to, distinguishing m6Am sites from non-sites.

### Graph signal is dominated by backbone adjacency

The RGCN edge-type ablation reveals that backbone adjacency, rather than long-range pairing, dominates the graph signal in m6Am prediction. We replaced the Graph Attention Network (GAT) used in preliminary experiments with a Relational Graph Convolutional Network (RGCN). GAT assigns type-agnostic attention shared across all edge types. RGCN, in contrast, uses relation-specific weight matrices, enabling differentiated message passing for backbone, base-pairing, and neighbor edges. The resulting edge-type hierarchy (backbone ≫ neighbor > pairing) would be invisible to GAT, which is the concrete reason the replacement matters: without separate edge types, the small but real contribution of pairing and neighbor edges would be averaged into a single “graph” number, and the design lesson—that linear adjacency already carries most of the graph signal—would be lost.

The edge ablation turns up a result we did not anticipate. Backbone edges alone reach AUC = 0.757 and MCC = 0.399, within 0.011 AUC of the full three-edge model (AUC = 0.768, MCC = 0.405), and adding pairing edges on top of backbone recovers the remainder of that gap; pairing edges alone reach AUC = 0.701. We had, with the broader field, anticipated that pairing edges would carry the structural signal, on the reasoning that PCIF1 recognizes a structured substrate; the data instead assign the graph signal to linear backbone adjacency. Two biological readings make this pattern coherent. First, PCIF1 recognizes the cap-proximal adenosine in its local linear context [2,3], so the backbone carries the information that the enzyme itself uses. Second, m6Am is installed co-transcriptionally, before the nascent RNA has folded into a stable 2D topology, so a linear-context representation is precisely the information available at the moment of recognition.

Because the two ablations described above remove structural information only partially—the edge-type ablation retains all structure-derived node features, and the node-feature ablation retains the pairing edges—neither isolates the contribution of secondary structure on its own. We therefore ran a direct control: the RNAfold dot-bracket strings were permuted across sequences (fixed seed), which destroys the correspondence between each window and its predicted structure while preserving the marginal distribution of structures, the 21-dimensional node-feature layout, and the graph edge statistics. Training and evaluation then proceeded through exactly the same code path, hyperparameters, fold splits and random seed as the unpermuted model. Permuting the structures left performance unchanged: mean test AUC across five folds was 0.7613 (true structures) versus 0.7679 (permuted), a paired difference of −0.0066 (*p* = 0.23, paired *t*-test over folds), with a correla*t*ion of *r* = 0.98 between the two sets of predictions. On the graph tower’s five-fold-averaged test predictions the AUCs were 0.7642 and 0.7704 respectively. If base-pairing information carried usable signal, destroying the window-to-structure correspondence should have degraded performance; it did not.

The three analyses therefore converge on a scoped conclusion: the graph tower’s contribution comes from the sequence-derived node features integrated through the relational message passing over local adjacency, not from the identity of each window’s predicted fold. The scope deserves emphasis. The control did not remove the structure-derived inputs altogether—both arms retained the same 21-dimensional node-feature layout and the same typed edges, and the RGCN’s message passing over the graph is itself a structural operation—so the graph-relational encoding of the tower remained present and constant throughout. What varied, and what we found dispensable, was only the correspondence between each window and its specific predicted secondary structure. We report this controlled result explicitly because it sharpens what structural encoding contributes on this task. Structural encoders are frequently added to RNA modification predictors on the expectation that the particular local fold governs recognition; our control separates fold identity from structural modelling—given that the pre-trained semantic representation already recovers the base-pairing annotation (probing analysis below), the invariance of the permutation reflects redundancy between the fold and the sequence-derived representation rather than the absence of structural information. Two caveats bound the claim. The result is specific to 41-nt windows folded in isolation; longer-range interactions that a full-length transcript could form are outside the scope of this benchmark, and a model operating on full-length or cross-window structure could behave differently. And the finding is specific to m6Am, whose substrate is defined largely by its position at the transcription start rather than by a structural motif; the same control might well give a different answer for modifications whose recognition depends on a hairpin or a bulge.

This raises an apparent paradox: the RGCN is the strongest single tower, yet each structure-derived feature group contributes marginally, and even removing the window-to-fold correspondence entirely leaves performance unchanged. The two observations resolve together. The structure-derived groups—SS state, loop type, pairing geometry, and the pairing edges—are largely determined by the input sequence (RNAfold is deterministic given a window), so they are heavily redundant with the sequence-derived signal that the tower already receives through nucleotide identity and positional encoding; removing any one group lets the remaining groups compensate, and uncoupling all of them from their sequences adds nothing, because the predictions rest on the sequence-derived features and on the relational aggregation over the graph. The tower’s margin over the non-graph representations is narrow, and is therefore best attributed to the graph-relational design itself—typed-edge message passing over local adjacency—rather than to any particular fold derived from RNAfold; it is on that basis that we describe the tower as a graph-relational encoder throughout rather than as a structural encoder. A probing analysis supports this reading: per-base base-pairing status is largely recoverable from the pre-trained RNA-FM embeddings (AUC 0.72 on held-out windows, versus 0.65 from the raw sequence alone), so the fold annotation the tower receives is substantially redundant with the semantic representation the ensemble already gets through the RNA-FM channel. This redundancy is not a deficiency of the structural channel but the expected consequence of a powerful pre-trained semantic representation, and it clarifies where genuinely new structural signal could help: inputs that the sequence-derived embeddings cannot recover, such as experimentally measured or long-range structural information (discussed below).

### Built-in interpretability via AUC-weighted fusion

The AUC-weighted ensemble provides an inherently interpretable fusion, as each prediction can be audited by inspecting tower weights rather than treating the model as a black box. Interpretability is a property of the architecture, not a post-hoc addition. The typed RGCN edges let us ask which structural relationship a prediction rests on (the edge-type hierarchy in the ablation analysis above); the AUC-weighted ensemble weights let us ask how much each tower contributed to a given prediction (Fig 11). The two views answer different questions—one about graph structure, one about tower contribution—and neither requires a separate post-hoc explainer trained to approximate the model. In practice this means a researcher who disagrees with a prediction can look at the tower weights and the edge-type ablation and form a specific hypothesis about what the model is “seeing,” rather than re-running the entire pipeline with a different explainer. The cost is that the weights and edge types are coarse-grained: they do not reach nucleotide resolution, which is why we added the IG-weighted logo in the interpretability analysis above as a complementary, model-specific attribution.

### Limitations and future work

#### Several limitations of TriTower-m6Am warrant discussion.

First, the sensitivity (0.869) comes at the cost of moderate specificity (0.531). In applications where false positives are costly (such as experimental validation pipelines), threshold adjustment, cost-sensitive learning, or hard-negative mining may be preferable, and these options grow in importance under the strongly imbalanced operating settings of the external evaluation, where average precision declines.

Second, the RGCN tower currently consumes RNAfold-predicted secondary structures, whose per-base-pair accuracy is estimated at ~73%. Our structural analyses place this limitation in perspective and point toward its resolution: because the fold annotation is largely redundant with the sequence-derived embeddings, what the graph tower stands to gain is not more accurate versions of the same predicted fold, but structural information that sequence alone cannot supply—experimentally measured structures (SHAPE-MaP and DMS-seq, chemical-probing assays that measure RNA structure in vitro), or full-length folding that captures long-range contacts invisible to a 41-nt window. Replacing predicted with measured structures is a natural and promising direction, for this architecture and for RNA-modification predictors generally, and we expect it to increase the marginal value of the structural channel.

Third, the node feature ablation (Fig 8) revealed redundancy between the SS state/pairing geometry features and the pairing edges, suggesting that a more compact node representation could be designed in future work. We retained the full feature set to preserve the interpretability of the contribution analysis and avoid introducing changes that would require re-validating the entire pipeline.

Fourth, RNA-FM embeddings are extracted from a frozen model. End-to-end fine-tuning of the RNA-FM backbone was not explored. The frozen backbone already accounts for most of the pipeline’s per-window cost (18.33 ms and a 391 MB peak, S1 Table), and adapting it to this task is a natural next step; parameter-efficient methods such as LoRA (low-rank adaptation) could enable task-specific adaptation at modest additional cost.

Fifth, the model is trained and evaluated exclusively on human m6Am data derived from HEK293/HEK293T cells (GEO accessions GSE63753 [9] and GSE78040 [17]). Although m6Am is conserved across vertebrates and PCIF1 is highly conserved across mammals, cross-species evaluation was not performed in this study for two reasons. First, experimentally validated m6Am site catalogs with single-nucleotide resolution remain scarce for non-human organisms, limiting the availability of independent test sets. Second, cross-species performance differences are difficult to interpret: a drop in performance could reflect genuine species-specific biology, but it could equally reflect biases in the reference transcriptome, differences in sequencing protocol, or systematic differences in 5′ untranslated region (5′UTR) length and GC content across species. To avoid confounding these factors, we prioritized the external BCA-derived negative evaluation (see the external evaluation above), which tests generalization to independent sequences within the same species while controlling for cap-proximal context. Dedicated cross-species evaluation with matched experimental protocols is an important direction for future work.

Sixth, the benchmark carries a systematic base-composition offset between the classes: mean GC content is 0.625 for positives versus 0.470 for negatives in the training set (0.638 versus 0.552 on the test set), and a position-independent dinucleotide-composition model already reaches AUC = 0.689 on the test set. A within-GC-strata adjustment lowers the ensemble’s AUC from 0.776 to 0.719, so most of the signal is position-specific, but a measurable part of the apparent discriminative power is compositional (see the Composition offset paragraph in the Results). This offset is a property of the published benchmark rather than of our preprocessing, and it is shared identically by every method trained on it; it also has a biological reading—m6Am-bearing transcripts initiate predominantly at GC- and CpG-rich promoters—which is why the external evaluation against BCA-derived negatives from the GENCODE transcriptome (see the external evaluation above) was designed as the control for exactly this concern.

Seventh, the external generalization evaluation (see the external evaluation above) extends only the negative-sample distribution to the GENCODE transcriptome, while the positive sites still originate from the same batch of miCLIP experiments used in the main test set. Cross-platform and cross-laboratory generalization (covering independent experimental techniques, distinct cell lines, and alternative sequencing protocols) is therefore not yet verified. Conclusions about deployment-ready performance should be tempered accordingly: an independent positive-site catalog generated by a different miCLIP or m6Am-seq protocol is needed to confirm that the model generalizes beyond a single experimental source.

Future directions include incorporating additional modalities such as genomic context and evolutionary conservation scores. Exploring end-to-end fine-tuning of RNA-FM with parameter-efficient methods and developing multi-task learning frameworks for joint prediction of m6A, m6Am, and other RNA modifications could further enhance predictive power. Extending the triple-tower architecture to other RNA modification prediction tasks (e.g., m5C [32], Ψ [33], N^1^-methyladenosine (m1A) [34]) will test its generalizability [35], and conducting experimental validation of high-confidence novel predictions will assess real-world discovery utility.

## Conclusion

We presented TriTower-m6Am, a triple-tower architecture that combines three representations of an RNA window—semantic (RNA-FM), sequential (One-Hot BiLSTM), and structural (RGCN with typed edges)—for m6Am site prediction. On the independent test set the model reaches AUC = 0.776 and MCC = 0.425 with 329,666 trainable parameters, 4.4 times smaller than the 1.45M-parameter DTC-m6Am baseline, and at matched specificity it consistently recovers a few additional true sites (Table 10). We therefore present the architecture’s contribution as auditable multi-representation fusion at a fraction of the parameter cost, together with the controlled structural analysis below.

The complementarity of the three towers is supported on two independent grounds. The CKA scores (0.55–0.67) are high enough that no tower is redundant but low enough that none is dispensable, and the parameter-matched wide single-tower baselines fall below the ensemble despite matching or exceeding its capacity—so the gain is not a capacity effect but a representation effect. The AUC-weighted voting weights are readable from the architecture, which makes each tower’s contribution to a prediction auditable without a post-hoc explainer.

Two findings from the ablations changed how we think about the problem. The RGCN’s typed edges expose a hierarchy in which linear backbone adjacency carries most of the graph tower’s edge-related signal, with base-pairing edges providing a small additional contribution—a result that would be invisible to a model with shared, type-agnostic attention and that is consistent with the co-transcriptional timing of PCIF1-mediated methylation. The permutation control adds the precise scope: what is dispensable is the correspondence between each window and its specific predicted fold, because the fold annotation is already carried by the sequence-derived representation (probing analysis), while the tower’s benefit rests on the relational encoding of local context over sequence-derived node features. Structural encoding is therefore best judged by the graph-relational machinery it provides: on this benchmark the fold identity itself adds no new information, so the value of the structural channel lies in how it aggregates local context, and in how much more it could contribute once fed with structural information that sequence alone cannot provide. Extending the triple-tower pattern to other RNA modifications would test whether this scoped finding generalizes, or whether it is specific to cap-proximal recognition.

## Supporting information

S1 TableComputational efficiency of each tower, the ensemble, and DTC-m6Am.All entries were measured on the same NVIDIA RTX 3060 (12 GB) with batch size 1, using 20 warm-up passes followed by 200 timed passes (CUDA events), with each component benchmarked in its own process so that no other component’s allocations are resident when its peak is sampled. The whole set was measured in three independent sessions and the times reported are the medians across them; the annotation ± is the within-session standard deviation, and parameters are the trainable parameter count. DTC-m6Am is given as a range because its inference time was bimodal across repeated isolated runs, whereas the TriTower-m6Am components showed no comparable variation. The ensemble row is measured directly rather than summed from the towers. The TriTower-m6Am rows cover only the three downstream encoders and exclude the per-window RNAfold secondary-structure prediction and frozen RNA-FM embedding extraction that deployment also requires, neither of which DTC-m6Am needs; both steps are quantified in the table. Those rows therefore understate the true cost of TriTower-m6Am and are not a like-for-like comparison with DTC-m6Am.(PDF)

## Abbreviations

m6Am: N^6^,2′-O-dimethyladenosine
GAT: Graph attention network
m6A: N^6^-methyladenosine
IG: Integrated gradients
m5C: 5-methylcytosine
LOGO: Leave-one-group-out
m1A: N^1^-methyladenosine
Inr: Initiator element
mRNA: Messenger RNA
PyG: PyTorch Geometric
ncRNA: Non-coding RNA
AUC: Area under the ROC curve
PCIF1: Phosphorylated C-terminal domain (CTD)-interacting factor 1
ROC: Receiver operating characteristic
CTD: C-terminal domain
PR: Precision–recall
CAPAM: Cap-specific adenosine methyltransferase
AP: Average precision
FTO: Fat mass and obesity-associated protein
MCC: Matthews correlation coefficient
miCLIP: Methylation individual-nucleotide-resolution cross-linking and immunoprecipitation
SN: Sensitivity
m6Am-seq: M6Am-specific sequencing assay
SP: Specificity
SHAPE-MaP: In vitro RNA structure probing by mutational profiling
F1: Harmonic mean of precision and recall
DMS-seq: In vitro RNA structure probing by dimethyl sulfate
ACC: Accuracy
BCA: The cap-proximal BCA motif (B = C/G/U)
EMA: Exponential moving average
SS: Secondary structure
OOF: Out-of-fold
UTR: Untranslated region
AMP: Automatic mixed precision
SHAP: SHapley Additive exPlanations
CNN: Convolutional neural network
k-mer: A k-length nucleotide subsequence
CKA: Centered kernel alignment
RNA-FM: RNA foundation model
GELU: Gaussian error linear unit
BiLSTM: Bidirectional long short-term memory
LoRA: Low-rank adaptation
RGCN: Relational graph convolutional network
