# Supplementary material for: TriTower-m6Am: A triple-tower heterogeneous deep learning architecture integrating semantic, sequential, and structural information for mRNA N6,2′-O-dimethyladenosine site prediction
Source: PLoS Comput Biol. 2026 Nov 5;22(11):e1014939. doi: 10.1371/journal.pcbi.1014939 (PMC13766619; doi:10.1371/journal.pcbi.1014939)
Supplement: Supplementary file 1 [file pcbi.1014939.s001.pdf]

S1 Table

Computational efficiency of each tower, the ensemble, and DTC-m6Am. All entries were measured on the same NVIDIA RTX 3060 (12 GB) with batch size 1, using 20 warm-up passes followed by 200 timed passes (CUDA events). The whole set was measured in three independent sessions, and the times reported are the medians across them; the annotation ± is the within-session standard deviation. DTC-m6Am is given as a range because its inference time was bimodal across repeated isolated runs under this protocol — either about 3.6 ms or about 8.5 ms, each mode internally reproducible, and about 23 ms when deterministic cuDNN algorithms are forced — whereas the TriTower-m6Am components showed no comparable variation. The parameter and peak-memory comparisons are unaffected. Each component was measured in its own process, so that no other component's allocations are still resident when its peak is sampled. Parameters are the trainable parameter count of each tower's downstream encoder; the RNA-FM backbone is frozen and excluded.

Component	Parameters	Inference time (ms)	Peak GPU memory (MB)
RNA-FM tower	140,974	2.24 ± 0.41	9.89
One-Hot tower	143,489	0.94 ± 0.19	41.52
RGCN tower	45,203	8.61 ± 0.68	9.33
TriTower ensemble	329,666	12.41 ± 1.26	42.35
DTC-m6Am	1,446,256	3.6–8.5	15.63

Why fewer parameters do not buy lower latency or smaller peak memory. Three separate effects explain the direction of these numbers, and none of them is a consequence of parameter count.

Latency. The ensemble's time is the sum of three heterogeneous encoders executed in series, not the cost of one wider model. The RGCN tower dominates it at 8.61 of the 12.41 ms: relational message passing over three edge types on 41-node graphs at batch size 1 is a latency-bound scatter/gather workload that leaves the GPU largely idle, and its 45K parameters are the smallest of the three. By contrast DTC-m6Am is a single homogeneous stack of dense convolutions, which cuDNN executes with high utilisation.

Peak memory. At batch size 1, peak memory is a high-water mark of live allocations and is set by fixed library workspaces rather than by weight storage. The One-Hot tower — the fastest component — reports the largest peak of the three (41.52 MB, against 9.89 and 9.33 MB) because PyTorch's cuDNN LSTM path reserves a ∼32 MB recurrent workspace regardless of the model's size; disabling cuDNN drops the same measurement to 9.70 MB. The ensemble contains that same LSTM, so its high-water mark sits just above the One-Hot row (42.35 against 41.52 MB) even though the ensemble's own weight storage is under 1 MB. The DTC-m6Am figure is lower because a convolutional network of this size needs no comparable workspace. Peak memory at this batch size therefore cannot be read as a proxy for model size.

Scope of the measurement, quantified. The TriTower-m6Am rows above cover only the three downstream encoders. Deployment additionally requires two per-window preprocessing steps that DTC-m6Am does not incur. Rather than merely declaring them excluded, we measured both.

RNAfold secondary-structure prediction costs **2.81 ms** per window (median over 50,000 windows). This was measured with 15 parallel workers, so CPU contention inflates each individual call; the minimum single-call time observed was 1.62 ms, and 2.81 ms should therefore be read as an upper bound on the uncontended latency.

Extraction of the frozen RNA-FM embeddings is the larger of the two. A single forward pass at batch size 1 — the deployment case, one new window at a time — takes **18.33 ms** and peaks at **391.46 MB**, against 12.41 ms and 42.35 MB for the entire three-tower ensemble. The same step amortises to 2.52 ms per window when batched at size 8 (20.12 ms per batch of 8, peak 417.28 MB), which is the setting used for the offline extraction in this study; the two figures are reported separately because they answer different questions.

Adding the three stages gives a per-window cost of roughly **33.6 ms** for a single new prediction, against 3.6–8.5 ms for DTC-m6Am, with peak memory dominated by the RNA-FM forward pass at approximately **391 MB** against 15.63 MB. The model-size advantage of TriTower-m6Am is real; the runtime and memory disadvantages are real, several times larger than the encoder-only rows convey, and architectural in origin.
