# Supplementary material for: TriTower-m6Am: A triple-tower heterogeneous deep learning architecture integrating semantic, sequential, and structural information for mRNA N6,2′-O-dimethyladenosine site prediction
Source: PLoS Comput Biol. 2026 Nov 5;22(11):e1014939. doi: 10.1371/journal.pcbi.1014939 (PMC13766619; doi:10.1371/journal.pcbi.1014939)
Supplement: Supplementary file 2 [file pcbi.1014939.s003.pdf]

<u>Paper title:</u> TriTower-m6Am: a triple-tower heterogeneous deep learning architecture integrating semantic, sequential, and structural information for N6,2'-O-dimethyladenosine site prediction

<u>Reviewer decision:</u> Accept with minor revision

This paper presents TriTower-m6Am, a triple-tower architecture model that combines three independent encoders: a pretrained RNA language model for semantic patterns (RNA-FM with BellPooling), a bidirectional LSTM for local nucleotide order (One-Hot BiLSTM), and a relational graph convolutional network for the structural fold (RGCN with three typed edges) for predicting N6,2'-O-dimethyladenosine (m6Am) sites. The model fuses the outputs of the three encoders by AUC-weighted voting, so the contribution of each tower to a given prediction is a readable number rather than a hidden parameter or black box that emits a prediction without explaining which features drove it. The paper is well written with rich presented analysis and the results of the presented paper approach are promising. Therefore, I recommend publishing this paper in PLOS Computational Biology Journal after addressing the following question:

-I see the authors have compared the performance of TriTower-m6Am to the best previous model (DTC-m6Am) in terms of AUC, MCC, SN, SP, and F1 as reported in Table 4. However, I do not see a comparison between the processing time of TriTower-m6Am and the DTC-m6Am. I think providing such comparison would be also worthy.

<u>Minors:</u>

-The authors provide a list of abbreviations in the end of the paper which is nice. However, I prefer writing the full expression that the abbreviation refers to in the paper body instead of relying only on the list of abbreviations as this will save the time for the reader.
-Figure 14 is missing legend. What do dark orang and blue dots represent for positive and negative predictions respectively? Also, what black and blue lines refer to? More clarification should be provided by the authors.
- In page 23, the authors mentioned that the model is trained and evaluated exclusively on human m6Am data. Which human cell lines data?
