# Supplementary material for: TriTower-m6Am: A triple-tower heterogeneous deep learning architecture integrating semantic, sequential, and structural information for mRNA N6,2′-O-dimethyladenosine site prediction
Source: PLoS Comput Biol. 2026 Nov 5;22(11):e1014939. doi: 10.1371/journal.pcbi.1014939 (PMC13766619; doi:10.1371/journal.pcbi.1014939)
Supplement: Supplementary file 3 [file pcbi.1014939.s005.pdf]

<u>Paper title:</u> TriTower-m6Am: a triple-tower heterogeneous deep learning architecture integrating semantic, sequential, and structural information for N6,2'-O-dimethyladenosine site prediction

<u>Reviewer decision:</u> Accept with minor revision

The authors applied my comment that asked them to report about the processing time of TriTower-m6Am against the best previous model DTC-m6Am by providing the supplementary table S1 Table which is good. However, I do not understand why the comparison is favourable to TriTower-m6Am if it has lower number of parameters and higher inference time (ms) and Peak GPU memory (MB) in comparison to DTC-m6Am (The inference time and Peak GPU memory of TriTower-m6Am are **10.38 ms and 41.62 MB** respectively, while the inference time and Peak GPU memory of DTC-m6Am are **8.34 ms and 21.29 MB** respectively)? Therefore, I would prefer further explanation and clarification from authors for this point based on the TriTower-m6Am and DTC-m6Am architectures.

<u>Minors:</u>

I am still seeing some abbreviations used for the first time in the paper body without explanation such as Low-Rank Adaptation (LoRA), Centered Kernel Alignment (CKA), CAPAM, FTO, GELU, RGCN, PCIF1, RNA-FM.
