# Supplementary material for: TriTower-m6Am: A triple-tower heterogeneous deep learning architecture integrating semantic, sequential, and structural information for mRNA N6,2′-O-dimethyladenosine site prediction
Source: PLoS Comput Biol. 2026 Nov 5;22(11):e1014939. doi: 10.1371/journal.pcbi.1014939 (PMC13766619; doi:10.1371/journal.pcbi.1014939)
Supplement: Supplementary file 4 [file pcbi.1014939.s006.pdf]

Response to Reviewers — PCOMPBIOL-D-26-02002

Manuscript: TriTower-m6Am: a triple-tower heterogeneous deep learning architecture integrating semantic, sequential, and structural information for mRNA N6,2’-O-dimethyladenosine site prediction **Decision:** Major Revision

---

Response to the Editor

We thank the editor and the reviewers for a careful and constructive assessment. The comments prompted us to re-examine several of our own numbers, and that re-examination uncovered three errors that we have corrected. Because the corrections change the strength of our claims, we describe them at the outset rather than burying them in the point-by-point responses.

Three corrections.

1. **Threshold selection.** All threshold-dependent metrics (MCC, SN, SP, F1) were computed with a decision threshold that had been optimised on the test set, whereas the Methods section describes selection on balanced out-of-fold predictions. We corrected the implementation and recomputed every threshold-dependent value in the paper. The analyses affected, and now recomputed under a single consistent protocol, are: the main comparison (Table 4), the tower ablation (Table 5), the edge-type ablation (Table 6), the node-feature ablation, the multi-seed stability analysis (Table 8), the training-data-availability analysis (Table 9, re-run), the pos_weight sensitivity analysis (re-run), and the external imbalance evaluation. Most corrected values are lower than those originally reported; a few change in the other direction, and we report all values as recomputed.
2. **DTC-m6Am row of Table 4.** The sensitivity and specificity for DTC-m6Am (0.800 and 0.530) had been taken from the original publication rather than measured on our re-evaluated checkpoint. We have re-evaluated the official DTC-m6Am checkpoint on the identical 640-sequence test set to obtain the correct values: 0.878 and 0.503. We have updated Table 4 with these measurements and added a table note clarifying the threshold-selection protocol.
3. **Structural channel.** The ablation analyses reported in the original submission do not isolate the contribution of secondary structure. We ran the missing control, and it shows that the contribution attributable to the specific base-pairing assignment is negligible, while the graph-relational encoding of the tower (typed-edge message passing and structure-derived node features, both retained in every arm of the control) remains intrinsic to its contribution.

We have rewritten the Abstract, Results, Discussion and Conclusion so that every claim matches the corrected numbers, and we have reframed the contribution accordingly.

Editorial comment 1

> The structural tower relies on RNAfold predictions from isolated 41-nt sequence windows, and the current ablation analyses do not clearly establish the extent to which the RGCN’s performance is specifically attributable to secondary-structure information.

We agree on both counts, and we thank the editor for identifying the gap in the ablation design. Neither of our original ablations isolates structure: the edge-type ablation removes pairing

edges but retains all structure-derived node features, and the node-feature ablation removes structure-derived features but retains the pairing edges.

We added a direct control. The RNAfold dot-bracket strings were permuted across sequences (fixed seed), which destroys the correspondence between each window and its predicted structure while preserving the marginal distribution of structures, the 21-dimensional node-feature layout, and the graph edge statistics. Training and evaluation then ran through exactly the same code path, hyperparameters, fold splits and random seed.

Result. Permuting the structures left the graph tower's performance unchanged: mean test AUC across five folds was 0.7613 with the true structures versus 0.7679 with permuted structures, a paired difference of -0.0066 ($p = 0.23$, paired t-test over folds), with $r = 0.98$ between the two prediction sets. On the graph tower's five-fold-averaged test predictions the AUCs were 0.7642 and 0.7704. If base-pairing information carried usable signal, destroying the window-to-structure correspondence should have degraded performance; it did not.

Interpretation. The graph tower's contribution comes from the sequence-derived node features integrated through the relational message passing over local adjacency, not from the identity of each window's predicted secondary structure. The scope matters here: both arms of the control retained the same node-feature layout and the same typed edges, so the tower's graph-relational encoding—itself a structural form of modelling, in that it determines how information is aggregated over the graph—remained constant; what we found dispensable is only the window-to-fold correspondence. We now report this as a scoped negative result and have reframed the tower accordingly: it is a graph-relational encoder over the 41-nt window whose benefit does not depend on the specific predicted fold, rather than a structural encoder in the sense that the fold itself drives prediction. We have also replaced the section heading "RGCN structural tower" with "RGCN graph tower" and revised the Introduction's motivation, which previously asserted that the local fold governs PCIF1 recognition, to state that this is an assumption we test rather than a premise we rely on.

A supporting probe. A probing analysis gives the scoped result a constructive reading rather than a purely negative one: per-base base-pairing status is largely recoverable from the pre-trained RNA-FM embeddings (AUC 0.72 on held-out windows, versus 0.65 from the raw sequence alone, logistic-regression probe), so the fold annotation the tower receives is substantially redundant with the sequence-derived semantic representation the ensemble already gets through the RNA-FM channel. In other words, the information carried by the specific fold is not new to the model; what the tower adds is the relational aggregation over local adjacency. This is why permuting the structures changes nothing while removing or reweighting the tower still matters. We have added this analysis to the Discussion.

We have also added the rationale for the isolated-window design that the editor and Reviewer #1 both asked for, and we now state the limitation explicitly: the result is specific to 41-nt windows folded in isolation, and a model operating on full-length or cross-window structure could behave differently.

Revised manuscript text (Discussion):

> *Because the two ablations described above remove structural information only partially—the edge-type ablation retains all structure-derived node features, and the node-feature ablation retains the pairing edges—neither isolates the contribution of secondary structure on its own. We therefore ran a direct control: the RNAfold dot-bracket strings were permuted across sequences (fixed seed), which destroys the correspondence between each window and its predicted structure while preserving the marginal distribution of structures, the 21-dimensional node-feature layout, and the graph edge statistics. [...] If base-pairing information carried usable signal, destroying the window-to-structure correspondence should have degraded performance; it did not.*

Editorial comment 2

> The reported AUC improvement is relatively modest (0.776 vs. 0.765) and is not supported by confidence intervals or direct statistical significance testing.

We agree, and we have added a complete uncertainty and significance analysis.

Uncertainty. Stratified bootstrap confidence intervals (2,000 resamples, positives and negatives resampled separately) for the ensemble AUC on the independent test set: 0.7755 [0.7388, 0.8104]. Threshold-dependent metrics (SN, SP, F1, MCC) are single operating points at the fixed balanced-OOF threshold and are reported in Table 4 (SN 0.869, SP 0.531, F1 0.743, MCC 0.425); they are operating-point-specific, so the statistical comparison in this revision rests on the threshold-independent AUC and on McNemar's test over the classification decisions.

Significance. We re-derived per-sample predictions from the DTC-m6Am official checkpoint and verified that both models were evaluated on the identical 640 sequences in the same order. We then applied a paired bootstrap test to the AUC difference and McNemar's test to the classification decisions:

Test	Result
ΔAUC (paired bootstrap)	+0.0105, 95% CI [−0.0093, +0.0293], $p = 0.27$
McNemar, both models at threshold 0.5	$b = 42$, $c = 37$, $p = 0.65$
McNemar, each model at its own validation-selected threshold	$b = 88$, $c = 83$, $p = 0.76$

The AUC improvement is not statistically significant, and the two models are not distinguishable in their classification decisions. We no longer assert an advantage in discrimination.

A threshold-free comparison. Because a single-threshold comparison depends on the operating point, we added a comparison at matched specificity, which does not depend on any threshold-selection procedure (Table 10, Fig. 16). At matched specificity between 0.40 and 0.70, TriTower-m6Am recovers 1 to 7 additional true positives out of 320 (mean 3.5); the bootstrap 95% confidence interval includes zero at every operating point. We report this as a consistent but modest trend, not a demonstrated gain.

Editorial comment 3

> There is also an inconsistency between the manuscript's interpretation of the standalone models and the results presented in Table 5.

The editor is correct. The sentence in question read "no single encoder in the comparison clears MCC = 0.40 on its own while the ensemble reaches 0.44". Because the manuscript uses "encoder" to denote a tower, the sentence directly contradicted Table 5, where the standalone RGCN reaches MCC = 0.405.

We have rewritten it to refer unambiguously to the three standard deep-learning baselines trained on one-hot input (CNN 0.355, BiLSTM 0.342, Transformer 0.322, corrected protocol-B values), and we have added an explicit statement that the ensemble's advantage over its strongest single tower is small (AUC 0.776 vs. 0.768; MCC 0.425 vs. 0.405) and that we therefore do not claim that fusion dominates single-tower modelling in ranking quality. We now present interpretability and audited weights, not a large performance margin, as the principal benefit of the ensemble.

Revised manuscript text:

We read this as evidence that the lift does not come from picking the right architecture in isolation—it comes from combining heterogeneous representations: none of the three one-hot baselines reaches MCC = 0.40 on its own, whereas the full ensemble reaches 0.43. Even against our strongest single tower (the RGCN graph tower, which also receives structural and relational input and is not one of these baselines) the ensemble's advantage is small—AUC 0.776 vs. 0.768; MCC 0.425 vs. 0.405—and we therefore do not claim that fusion dominates single-tower modeling in ranking quality; the interpretability and audited weights, not a large performance margin, are the principal benefits of the ensemble.

Editorial comment 4

> (a) some Materials and Methods sections appear unnecessary, (b) other important methodological details are insufficiently described or omitted, (c) at least one figure is not adequately referenced or discussed in the main text, and (d) multiple typographical errors require correction.

(a) Methods structure. We agree that the Methods carried more textbook-definition material than the paper needs, and we have trimmed four passages, moving the detail to the Supporting Information rather than deleting it: the MCC formula (now a one-line statement); the enumeration of edge counts and pairing rules in the graph construction; the six-stage layer-by-layer description of the RNA-FM encoder; and the scikit-learn API specifics in the cross-validation subsection. Together these remove roughly 18 lines of standard definitions. Against that, we have added approximately 15 lines of content that the reviewers specifically asked for: the dataset provenance chain, the 41-nt window rationale, the cell-line source, the threshold-selection procedure, and the negative-sample composition caveat. The Methods is therefore about the same length as before but substantially more specific. We did not compress the three tower descriptions, the Dataset subsection, or the hyperparameter table.

(b) Missing methodological detail. We have added or clarified: the dataset provenance chain and the exact definition of the negative class; the construction of the 41-nt window and the meaning of the 20 upstream positions; the threshold-selection procedure (balanced out-of-fold predictions, selected once and fixed); the origin of the 640-dimensional RNA-FM embedding (the backbone's hidden dimension); the cell-line source; the acronym expansions requested by Reviewer #2; and the negative-sample composition caveat. All of these are now stated in the Methods and Limitations rather than left implicit.

(c) Figure referencing. Figure 4 was indeed never cited in the running text. We have added the citation and an accompanying sentence. While checking, we found that Figure 7 was in the same position, and we have added a citation for it as well.

(d) Typographical errors. We have corrected the misspelling of "Tower" in Fig. 1a and expanded several acronyms at first use (GAT, ACC).

Response to Reviewer #1

We thank the reviewer for a detailed and technically precise report. Acting on comments 2 and 4 led us to re-examine our own evaluation code, and we found three errors that we have corrected. Two of them change the strength of our claims, and we describe the corrections in full in the relevant responses below.

Comment 1

> The RGCN uses secondary structures predicted by RNAfold separately for each 41-nt window. Therefore, the predicted structure represents the isolated 41-nt frag-

> ment and excludes any interactions with nucleotides outside the ±20-nt window. While the authors acknowledge the general uncertainty of RNAfold predictions in the Limitations (p. 23), they do not address whether folding an isolated 41-nt fragment adequately represents the structural environment of the same region within the full-length mRNA. Please provide a rationale for using the 41-nt window for secondary-structure prediction and discuss how the exclusion of longer-range interactions may affect the structural features used by the RGCN.

We agree that the original submission asserted the framework without justifying the window size or discussing what the isolation excludes. We have added both.

Rationale. The 41-nt extent is inherited rather than chosen by us, and we now say so explicitly. Three considerations support keeping it. First, comparability: the entire method lineage against which we benchmark (m6AmPred, DLm6Am, m6Aminer, DTC-m6Am) uses 41-nt windows centred on the candidate site, so changing the window would invalidate every comparison in Table 4. Second, convention: $±$20 nt around a candidate site has been the standard context length in RNA-modification prediction since SRAMP, and the m6AmPred study reported that a 41-nt window with the modification site at the centre performed best among the lengths it tested. Third, deliberate non-tuning: we chose not to re-optimise window length, so that the comparison to prior methods stays like-for-like. Window-length ablation is now listed explicitly as future work.

What the isolation excludes. The predicted fold of an isolated 41-nt fragment cannot contain base pairs with nucleotides outside the window, so any long-range or tertiary interaction that the same region would form in the full-length transcript is absent from the features. We now say this in the Discussion rather than only noting RNAfold's per-base-pair accuracy.

How much this matters empirically. The control experiment reported under comment 2 bears directly on this point, and it is more informative than the rationale itself: permuting the predicted structures across sequences did not change performance (ΔAUC = −0.006, p = 0.23). Whether the structures are the true isolated-window folds or entirely mismatched folds makes no measurable difference to the model. This bounds how much the long-range-interaction limitation can matter here: if the model derives nothing from the correspondence between a window and its predicted structure, it is also not being harmed by long-range pairs that are absent from those structures. We now present the limitation and this bound together.

Revised manuscript text (Discussion):

> *The result is specific to 41-nt windows folded in isolation; longer-range interactions that a full-length transcript could form are outside the scope of this benchmark, and a model operating on full-length or cross-window structure could behave differently.*

Comment 2

> [...] Therefore, the current ablations do not clearly establish how much of the RGCN's predictive performance is specifically attributable to secondary-structure information rather than sequence and positional information. The authors should revise this interpretation or provide an analysis that isolates the contribution of secondary-structure information.

This is the most consequential comment in the report, and the reviewer's diagnosis of the ablation design is exactly right: the edge-type ablation varies only the retained edge type while leaving the full node-feature representation, including structure-derived features, available to the model, and the node-feature ablation varies only the feature groups while leaving the pairing edges in place. Neither isolates structure.

We ran the missing control. The RNAfold dot-bracket strings were permuted across sequences with a fixed seed. This destroys the correspondence between each window and its pre-

dicted structure while preserving the marginal distribution of structures, the 21-dimensional node-feature layout, the edge-type definitions, and the graph statistics. Training and evaluation then ran through the identical code path, hyperparameters, fold splits and random seed, so the only difference between the two arms is whether the structure belongs to the sequence it is paired with.

Arm	Mean test AUC (5 folds)	Five-fold-averaged graph-tower AUC
True structures	0.7613	0.7642
Permuted structures	0.7679	0.7704

Paired difference across folds: −0.0066 (95% CI includes zero; $p = 0.23$, paired t-test). Correlation between the two prediction sets: $r = 0.98$.

Interpretation. If base-pairing information carried usable signal, destroying the window-to-structure correspondence should have degraded performance. It did not, and if anything the permuted arm performed marginally better, which is within fold-to-fold variation. The graph tower's predictive power therefore comes from the sequence-derived node features (nucleotide identity, positional encoding) integrated through the relational message passing over the adjacency edges. A caveat on scope, which we also state in the manuscript: the control does not show that the graph-relational encoding is dispensable—both arms retained the same node-feature layout and the same typed edges, and the message passing over the graph is itself a structural operation—only that the correspondence between each window and its specific predicted fold is. We have revised the interpretation accordingly, and we no longer describe the tower as a structural encoder in the sense that the fold drives prediction: it is described throughout as a graph-relational encoder over the 41-nt window.

Supporting evidence from the existing ablations. Two results already in the manuscript point the same way and are now presented as such: the node-feature ablation shows that removing the SS state changes AUC by −0.002 and removing pairing geometry by +0.002, and the edge-type ablation shows that backbone-only reaches AUC 0.757 against 0.768 for the full three-edge model. Read together with the permutation control, these three analyses converge.

We report this as a scoped negative result rather than downplaying it, because it is informative: structural encoders are frequently added to RNA modification predictors on the assumption that the specific local fold matters, and on this benchmark we find no support for that specific assumption—even though the graph-relational machinery such an encoder provides continues to carry the tower's measured benefit.

Revised manuscript text (Discussion):

> *We report this as a scoped negative result because it is informative for the field. Structural encoders are frequently added to RNA modification predictors on the expectation that the particular local fold governs recognition, and on this benchmark that particular expectation is not supported. Two caveats bound the claim. The result is specific to 41-nt windows folded in isolation [...] And the finding is specific to m6Am, whose substrate is defined largely by its position at the transcription start rather than by a structural motif; the same control might well give a different answer for modifications whose recognition depends on a hairpin or a bulge.*

Comment 3

> Given that m6Am is described here as occurring at the first transcribed nucleotide, it is unclear what the 20 upstream nucleotides represent in these windows. The au-

> thors should clearly state how the upstream sequence was defined in the benchmark and explain its biological relevance to the prediction task.

We agree that the original description was insufficient, and we thank the reviewer for pressing on a point that bears on how the benchmark should be interpreted. We have now traced the benchmark's provenance and examined the windows directly.

Provenance. The dataset was assembled by Jiang et al. (m6AmPred), who defined the 41-nt window centred on the candidate adenosine; Liu et al. (m6Aminer) applied CD-HIT redundancy removal at 0.8 identity to produce the 3,700/37,000 training split and the 320/320 test split; DTC-m6Am released that benchmark unchanged, and we inherited it verbatim. We verified the inheritance: all parseable test negatives match m6Aminer's published negative set exactly.

What the upstream 20 nt are. Because a reference transcript set contains multiple 5′ isoforms per gene, the same adenosine can be the first transcribed nucleotide of a short isoform and an internal position of a longer one. Placing the 60 test positives against GENCODE v44, 53% of the full 41-mers fall inside annotated transcripts and 20% have their downstream 21-mer beginning at a transcript start. For most windows, the 20 positions on the 5′ side of the candidate are therefore the transcribed 5′ leader of a longer isoform rather than genomic promoter sequence. The strongest single piece of evidence for a cap-proximal context is the nucleotide immediately 5′ of the candidate: it is cytosine in 90.1% of positive windows, which matches the mammalian initiator (Inr) consensus BBCAB — precisely the context in which PCIF1 acts.

Where the negatives come from. The negatives are not arbitrary adenosines. They are BCA-motif adenosines — a non-A base at −2, cytosine at −1, the candidate adenosine at 0 — that were not called as modified in the same miCLIP experiments, so they share the motif that defines the positive class rather than being drawn from unrelated sequence. They differ from the positives in transcript position rather than in motif: the positives are cap-proximal, whereas the negatives sit at internal BCA sites, which is why the two classes differ in 5′ leader composition (reported under comment 6 below).

The second we can address now, and it is a substantive point in its own right. Because the candidate site is fixed to an adenosine in every sample, positive and negative, the model cannot classify by reading the candidate base. It must use the flanking context. We examined that context directly and found a systematic difference between the two classes: mean GC content is 0.625 for positive windows versus 0.470 for negative windows in the training set (0.638 vs. 0.552 on the test set). A single number, the overall GC content of the window, discriminates the two classes with AUC 0.676 on the test set, and a position-independent dinucleotide-composition model reaches AUC 0.689.

This does not invalidate the model, but it does mean that part of the discrimination available in this benchmark is compositional rather than position-specific. To quantify how much, we recomputed performance within strata of equal GC content: the ensemble's AUC falls from 0.776 to 0.719 after this adjustment, so a substantial part of the signal survives, but roughly a fifth of the apparent discriminative power (measured against the 0.5 reference baseline) is attributable to the composition difference. We now report this in the Results and discuss it in the Limitations.

Revised manuscript text (Discussion / Limitations):

> *Positive and negative windows differ systematically in base composition (mean GC 0.625 vs. 0.470 in training), and a single global GC value discriminates the classes at AUC 0.676. Adjusting for GC content within strata reduces the ensemble's AUC from 0.776 to 0.719, so a substantial part of the signal is position-specific, but part of the discrimination available in this benchmark is compositional rather than local.*

Comment 4

> [...] no confidence intervals or direct statistical comparison with DTC-m6Am are provided. Given the relatively small AUC difference on a 640-sample test set, the authors should provide appropriate uncertainty estimates and a statistical comparison to establish whether the reported improvement over DTC-m6Am is robust.

We thank the reviewer for this comment, which was the entry point for the corrections described here. The reviewer is right that neither uncertainty estimates nor a direct comparison were provided, and the specific figures quoted in the comment led us to re-examine the underlying numbers.

Uncertainty estimates. Stratified bootstrap confidence intervals for the ensemble AUC on the independent test set (2,000 resamples, positive and negative samples resampled separately, 320 each): AUC 0.7755 [0.7388, 0.8104]. Threshold-dependent metrics (SN, SP, F1, MCC) are single operating points at the fixed balanced-OOF threshold and are given in Table 4 (SN 0.869, SP 0.531, MCC 0.425); because they are operating-point-specific, the statistical claims in this report rest on the threshold-independent AUC and on McNemar's test over classifications.

Direct statistical comparison. We re-derived per-sample predictions from the DTC-m6Am official checkpoint and confirmed that both models had been evaluated on the same 640 sequences in the same order.

Test	Result
ΔAUC (paired bootstrap, 4,000 resamples)	+0.0105, 95% CI [−0.0093, +0.0293], $p = 0.27$
McNemar, both models at threshold 0.5	$b = 42$, $c = 37$, $p = 0.65$
McNemar, each model at its own validation-selected threshold	$b = 88$, $c = 83$, $p = 0.76$

The AUC difference is not statistically significant, and the two models are not distinguishable in their classification decisions.

A comparison that does not depend on the threshold. Because a single-threshold comparison is sensitive to the operating point, we added a comparison at matched specificity (Table 10, Fig. 16). At matched specificity in the range SP = 0.40–0.70, TriTower-m6Am recovers 1 to 7 more true positives out of 320 (mean 3.5). The bootstrap 95% confidence interval for this difference includes zero at every operating point, so we report it as a consistent but modest trend rather than as a demonstrated gain.

Two corrections found while preparing this analysis. First, the operating threshold had been selected by maximising MCC on the test set, whereas the Methods section describes selection on balanced out-of-fold predictions. All threshold-dependent metrics were consequently optimistic. We corrected the threshold-selection step and recomputed every such value. Second, the sensitivity and specificity reported for DTC-m6Am in Table 4 had been taken from the original publication rather than measured on our re-evaluated checkpoint; we have re-measured them at the default operating point of the official checkpoint (SN 0.878, SP 0.503) and updated Table 4 accordingly. The sensitivity advantage we originally reported does not survive this correction.

What we now claim. We have rewritten the Abstract, Results, Discussion and Conclusion. We no longer claim a sensitivity or AUC advantage over DTC-m6Am. The contributions we do claim are: auditable multi-representation fusion with 4.4 times fewer parameters than DTC-m6Am; a matched-specificity comparison showing a consistent but statistically unresolved

trend; and the scoped structural finding reported under comment 2 (the specific base-pairing assignment is dispensable, not the relational encoding).

Revised manuscript text:

> *At every matched specificity in the range SP = 0.40–0.70, TriTower-m6Am recovers slightly more true sites than DTC-m6Am: the difference corresponds to 1 to 7 additional true positives out of 320 (mean 3.5), which is small and whose bootstrap 95% confidence interval includes zero at every operating point. We therefore report the sensitivity difference as a consistent but modest trend rather than as a demonstrated gain.*

Comment 5

> The manuscript states on p. 10 that "no single encoder in the comparison clears MCC = 0.40" [...] However, Table 5 reports MCC = 0.439 for the standalone RGCN [...] The authors should correct this statement and reconsider the corresponding interpretation of the ensemble improvement.

The reviewer is correct, and we thank them for catching it. The sentence used "encoder", which the manuscript uses elsewhere to mean a tower, and therefore directly contradicted Table 5, where the standalone RGCN reaches MCC 0.405.

We have rewritten the sentence to refer unambiguously to the three standard deep-learning baselines trained on one-hot input (CNN 0.355, BiLSTM 0.342, Transformer 0.322). We accept the second half of the comment as well: the ensemble's advantage over its strongest single tower is genuinely small (AUC 0.776 vs. 0.768; MCC 0.425 vs. 0.405), and we no longer use that sentence to support a claim that fusion improves ranking quality. The ensemble's benefits, as we now present them, are interpretability and auditable per-prediction weights rather than a performance margin.

Revised manuscript text:

> *We read this as evidence that the lift does not come from picking the right architecture in isolation—it comes from combining heterogeneous representations: none of the three one-hot baselines reaches MCC = 0.40 on its own, whereas the full ensemble reaches 0.43. Even against our strongest single tower (the RGCN graph tower, which also receives structural and relational input and is not one of these baselines) the ensemble's advantage is small—AUC 0.776 vs. 0.768; MCC 0.425 vs. 0.405—and we therefore do not claim that fusion dominates single-tower modeling in ranking quality; the interpretability and audited weights, not a large performance margin, are the principal benefits of the ensemble.*

Comment 6

> **Minor comments:** On p. 11, the text states that removing BiLSTM produces the smallest AUC loss, whereas Table 5 shows the smallest decrease after removing RNA-FM (0.776 to 0.773). On p. 19, the external evaluation is described as being performed "without any retraining or threshold adjustment," but the same section reports MCC-optimal thresholds of 0.48, 0.67, and 0.81 [...] Figure 2 also includes ACC although the corresponding values are not reported in Table 4.

(a) Smallest AUC loss. The value 0.773 belongs to the column in which the BiLSTM is removed (RNA-FM retained, One-Hot blank, RGCN retained); the column with RNA-FM removed is 0.770. The apparent contradiction originates in the presentation of the table: the seven configurations were encoded only through checkmark rows, which made the column-to-value mapping hard to read, and we should not have relied on the reader to reconstruct

it. The column-wise deltas from the full ensemble are therefore −0.51 percentage points for RNA-FM, −0.27 for the BiLSTM and −0.68 for the RGCN (in AUC), so the original sentence ("pulling the BiLSTM costs the least") is consistent with the table.

That said, the reviewer's point is well taken: a table whose columns can only be identified by reading three checkmark rows across is badly presented. We have added explicit column headers (Full ensemble, w/o RNA-FM, w/o One-Hot, w/o RGCN, RNA-FM only, One-Hot only, RGCN only) and a ΔAUC row, so that the configuration-to-value mapping is stated rather than inferred. We have also made the units explicit throughout: the percentages in that paragraph are percentage points, not relative percentages.

(b) Threshold adjustment in the external evaluation. The reviewer is right that the original phrasing was inaccurate. No retraining occurred and all model weights were held fixed; the decision threshold was also held fixed, at the balanced out-of-fold value of 0.485 used throughout the study. What the original wording obscured is that the MCC-optimal operating point shifts markedly with the negative-to-positive ratio: if the threshold were re-optimised per ratio, it would move upward (0.48, 0.67 and 0.81 at 1:1, 1:10 and 1:100, respectively). We now state both facts explicitly and report the fixed-threshold values, so that the operating point is never tuned on the evaluation data.

Preparing this correction also surfaced a related and more serious problem: the same threshold-selection implementation had been optimising the threshold on the evaluation data rather than on held-out predictions. For the main experiments this has been corrected: the threshold is now selected on a balanced out-of-fold subset, fixed, and applied unchanged to the test set. Every threshold-dependent metric in the paper has been recomputed, and the corrected values are lower than those originally reported.

(c) ACC in Figure 2. The reviewer is right that there was a mismatch. We have removed the accuracy axis from Figure 2, so that the radar reports exactly the metrics tabulated in Table 4. We also removed the spoke labels from the figure, as requested in the corresponding comment below.

Revised manuscript text (external evaluation):

> *The trained 5-fold ensemble was applied without any retraining and without any threshold adjustment: all model weights were held fixed, and the decision threshold was held at the balanced out-of-fold value of 0.485 used throughout this study. We note that if the MCC-optimal operating point were re-tuned for each operating ratio, the threshold would shift upward (0.48, 0.67 and 0.81 at 1:1, 1:10 and 1:100, respectively); we report the fixed-threshold values here so that the operating point is not tuned on the evaluation data.*

Response to Reviewer #2

We thank the reviewer for a constructive report focused on clarity and on the biological interpretation of the benchmark. Acting on the point about the upstream context led us to a compositional analysis that we now report explicitly.

Comment 1

> If the modification only occurs on the first transcribed nucleotide, how do you center the 41-nt window at the candidate position? It would seem like you only have the 20-nt downstream to utilize. For clarity I think this needs to be explicitly expanded upon.

This is a fair challenge, and it is the same issue Reviewer #1 raises about the upstream context. We have now traced the benchmark's provenance and examined the windows directly, and

the short answer is that the window is centred on the candidate adenosine in sequence space rather than anchored on an annotated transcription start site.

Concretely: the benchmark was built by Jiang et al. (m6AmPred) and distributed unchanged through m6Aminer and DTC-m6Am. Placing the test positives against GENCODE v44, 53% of the full 41-mers fall inside annotated transcripts and 20% have their downstream 21-mer beginning at a transcript start — the remaining fraction reflects the fact that the reference set contains multiple 5′ isoforms per gene, so the same adenosine is cap-proximal for a short isoform and internal for a longer one. The 20 positions on the 5′ side are therefore mostly the transcribed 5′ leader of a longer isoform, not genomic promoter sequence. The immediate 5′ neighbour of the candidate is cytosine in 90.1% of positive windows, matching the mammalian initiator (Inr) consensus BBCAB.

We accept that the original Methods did not make this clear, and the revised Dataset subsection now states it.

What we can report now is the consequence, which we have examined directly. The candidate site is fixed to an adenosine in every sample, positive and negative, so the model cannot classify by reading that position. Everything it uses is flanking context. We decomposed the window and asked how much discriminative information each region carries, training a logistic regression on one-hot encodings of individual regions and evaluating on the held-out test set:

Region	AUC
Positions 0–19 (5′ flank)	0.645
Positions 21–40 (3′ flank)	0.718
Full window, excluding position 20	0.725

The 5′ flank carries real information, and notably it is not concentrated at the positions immediately adjacent to the candidate site: positions 16–19 give AUC 0.559, while the more distal positions 0–7 and 8–15 give 0.649 and 0.643. The signal is distributed across the window rather than localised at a motif.

We also found a systematic compositional difference between the classes: mean GC content is 0.625 for positive windows versus 0.470 for negative windows in the training set. A single global GC value discriminates the classes at AUC 0.676 on the test set. Adjusting for GC within strata reduces the ensemble's AUC from 0.776 to 0.719, so most of the signal is position-specific, but part of the discrimination available in this benchmark is compositional. We now report this and discuss what it implies for interpreting the model's performance.

Comment 2

> It's unclear to me where the 640 dimension of your matrix comes from. Please elaborate further.

The 640 is the hidden dimension of the RNA-FM backbone. For each 41-nt window the frozen backbone produces a per-nucleotide embedding, giving a 41 × 640 matrix, which the downstream encoder then compresses to 48 dimensions through layer normalization, a linear projection to 96, three parallel 1-D convolutions, and pooling. The original text stated the 41 × 640 shape but not what 640 was; we have added the clarification.

Revised manuscript text:

> *[...] we pre-compute the per-nucleotide embedding from the frozen RNA-FM backbone, which produces a 41 × 640 matrix, where 640 is the hidden dimension of the RNA-FM backbone [...].*

Comment 3

> I think a more thorough examination of the model predictions would greatly improve the clarity of your argument. The addition of a table or venn-diagram would enhance the voracity of your claims, by clearly illustrating the samples that your model is correctly identifying that the mother models are leaving out.

We agree, and we have added both the table and the figure that the reviewer suggests. Rather than a Venn diagram we used a 2×2 contingency table of classification outcomes, which carries the same information but also supports a significance test, together with a matched-specificity comparison that isolates the class of samples the reviewer is asking about.

On the independent test set at the fixed balanced-OOF operating points (TriTower-m6Am at threshold 0.485, DTC-m6Am at its default 0.5), the 2×2 contingency table of classification outcomes has off-diagonal counts b = 88 (TriTower-m6Am correct, DTC-m6Am incorrect) and c = 83 (DTC-m6Am correct, TriTower-m6Am incorrect), i.e. McNemar p = 0.76 (see the statistical table in our response to Reviewer 1, comment 4).

We also report the comparison at matched specificity, which is the more informative form of the same question: at every matched specificity between SP 0.40 and 0.70, TriTower-m6Am recovers between 1 and 7 additional true positives out of 320, with a bootstrap confidence interval that includes zero at every operating point. We state plainly in the text that this difference is small and not statistically resolved.

One point in the reviewer's framing deserves explicit address: on the positive class specifically, at the fixed operating points TriTower-m6Am recovers 278 of the 320 true sites, close to DTC-m6Am's 281. The larger difference between the two models lies in the negative class, where TriTower-m6Am rejects 170 of the 320 negatives versus 161 for DTC-m6Am. We therefore do not claim a sensitivity advantage, and have rewritten the corresponding passages.

Comment 4

> Missing reference and explanation of Figure 4 in running text.

The reviewer is correct, and we thank them for catching it. Figure 4 (the per-metric tower ablation) was never cited in the running text. We have added a citation and a sentence of explanation at the point where the tower ablation is discussed.

While checking the remaining figures we found that Figure 7 (the per-metric edge-type ablation) was in the same position, and we have added a citation for it as well.

Comment 5

> In the discussion of Figure 5, the affect that removing components has on specificity needs to be explicitly stated. I think the explanation is eluded to in the AUC results, but should be addressed in the prose.

The reviewer is right that the text discussed only AUC changes while the figure reports specificity as well. We have added the specificity comparison: removing label smoothing lowers specificity by 0.06, while replacing BellPooling with mean pooling and removing EMA change specificity by less than 0.03. No component trades a specificity loss for its AUC gain, so the AUC ordering is not concealing a competing trade-off in the other direction.

Revised manuscript text:

> *[...] The corresponding effect on specificity mirrors the AUC ordering: removing label smoothing lowers specificity by 0.06, whereas replacing BellPooling with mean pooling and removing EMA change specificity by less than 0.03, so no component trades a specificity loss for its AUC gain.*

Comment 6

> The results presented in Figure 10 increase my concern about the 20-nt upstream described in a previous comment. These are clearly not randomly determined but are heavily GC skewed. I think this needs to be more explitily discussed.

We thank the reviewer for this observation, which proved especially useful in this revision. We should first clarify what Figure 10 shows, and then report what we found when we followed the concern through.

What Figure 10 is. Figure 10 is the Integrated-Gradients-weighted sequence logo for the one-hot BiLSTM tower. Letter heights encode attribution-weighted nucleotide frequencies across the top-50 highest-confidence positive predictions, not raw sequence frequencies. It is therefore a picture of where the model puts weight, rather than of what the input sequences look like.

The underlying concern is nevertheless correct. Prompted by the comment, we examined the actual base composition of the input windows. Positive and negative windows differ systematically: mean GC content is 0.625 for positives versus 0.470 for negatives in the training set (0.638 vs. 0.552 on the test set). A single global GC value discriminates the classes at AUC 0.676 on the test set, and a position-independent dinucleotide-composition model reaches AUC 0.689. So the reviewer's reading of a GC skew is substantiated, even though the figure they were reading shows attributions rather than composition.

We now report this in three places. First, we state the composition difference explicitly in the Results. Second, we quantify how much of the model's performance it accounts for by recomputing within strata of equal GC content, which reduces the ensemble's AUC from 0.776 to 0.719; most of the signal is therefore position-specific, but a substantial minority of the apparent discriminative power is compositional. Third, we discuss the implication in the Discussion: part of what this benchmark rewards is the ability to distinguish GC-rich from GC-poor contexts, which is correlated with, but not identical to, distinguishing m6Am sites from non-sites.

On the origin of the composition difference. We traced this, because it determines whether the observation is a defect of our preprocessing or a property of the data. It is a property of the published benchmark, not of anything we did. Both classes show the same within-class asymmetry between their upstream and downstream halves, so the effect is not produced by the window extraction. Comparing the benchmark against reference windows drawn from transcript-internal BCA motifs in GENCODE v44, the negative windows match the reference almost exactly (CpG observed/expected 0.313 versus 0.315), while the positive windows are markedly GC- and CpG-enriched (0.810). In other words the negatives are ordinary transcript-like sequence, and the positives sit in GC- and CpG-rich 5′ leaders.

That pattern has a biological reading rather than a technical one: m6Am-bearing transcripts initiate predominantly at CpG-island promoters, so a GC-rich 5′ leader is a genuine property of the context in which PCIF1 acts, and the model can legitimately use it. The offset is nevertheless inherited by every method trained on this benchmark, ours included, which is why we report it explicitly and why the external evaluation against BCA-derived negatives from the GENCODE transcriptome was designed as the control for exactly this concern.

Comment 7

> Typo in Figure 1a: RNA-FM Twoer should be Tower

Corrected. We thank the reviewer for spotting it. The misspelling is in the text layer of the Fig. 1a graphic rather than in the manuscript source, which is why it survived our proofreading; the figure has been corrected and regenerated.

Comment 8

> Please explain acronyms before you use them for the first time or provide definition in parentheses, GAT, line 97.

Corrected. GAT is now expanded at its first occurrence ("Graph Attention Network (GAT)"). We audited the remaining abbreviations and found that ACC was also used in the text without expansion; it is now defined at first use as well. The list of abbreviations at the end of the paper is retained.

Comment 9

> I don't think Equation 5 is necessary. I think MCC is relatively well known and or can be easily looked up.

Agreed and removed. The MCC formula is replaced by a one-line statement that MCC is computed from the confusion matrix at the operating threshold. Equation numbering has been adjusted accordingly.

Comment 10

> The angle labels on Fig 2 clutter the figure, please remove these.

Removed. Figure 2 has been regenerated: the spoke angle labels are gone and each metric is now labelled at the rim of the axis instead. In the same regeneration we removed the accuracy axis, which also resolves the mismatch between Figure 2 and Table 4 that Reviewer #1 noted. The values plotted have been updated to the recomputed threshold-dependent metrics.

Response to Reviewer #3

We thank the reviewer for the positive assessment and for four specific, actionable points. All four have been addressed.

Comment 1

> I do not see a comparison between the processing time of TriTower-m6Am and the DTC-m6Am. I think providing such comparison would be also worthy.

Agreed. We have measured both models under identical conditions on the same GPU (NVIDIA RTX 3060, 12 GB), batch size 1, three warm-up passes followed by ten timed passes using CUDA events, and extended the supporting table accordingly.

Model	Parameters	Inference time (ms)	Peak GPU memory (MB)
RNA-FM tower	140,974	2.09	9.89
One-Hot tower	143,489	0.80	41.62
RGCN tower	45,203	7.49	9.44
**TriTower ensemble**	**329,666**	**10.38**	**41.62**
**DTC-m6Am**	**1,446,256**	**8.34**	**21.29**

Two points of interpretation, both stated in the table caption. First, TriTower-m6Am uses 4.4 times fewer parameters. Second, the comparison is favourable to TriTower-m6Am: its 10.38 ms covers only the three downstream encoders, and excludes both RNAfold secondary-structure prediction and extraction of the frozen RNA-FM embeddings, whereas DTC-m6Am

requires neither step. The reported ratio should therefore be read as a lower bound on TriTower-m6Am's runtime cost.

One feature of the breakdown is worth flagging: the RGCN tower accounts for 7.49 of the 10.38 ms, that is about 72% of the total. Given the scoped structural finding reported elsewhere in this revision—the specific base-pairing assignment contributes little, while the relational encoding remains intrinsic to the tower—we note that the most expensive component of the pipeline is the one whose structure-specific input has the least measurable benefit, and that a lighter replacement is a natural direction for future work.

Comment 2

> The authors provide a list of abbreviations in the end of the paper which is nice. However, I prefer writing the full expression that the abbreviation refers to in the paper body instead of relying only on the list of abbreviations as this will save the time for the reader.

Agreed. We audited every abbreviation against the list and expanded each at first use in the body, including several that had been used without definition (GAT, ACC, and BiLSTM). The list at the end of the paper is retained as a convenience.

Comment 3

> Figure 14 is missing legend. What do dark orang and blue dots represent for positive and negative predictions respectively? Also, what black and blue lines refer to? More clarification should be provided by the authors.

The reviewer is right that the legend was uninformative. The figure caption now states each element:

- **Red violin and dots** — the ten highest-confidence positive predictions (highest ensemble probability).
- **Blue violin and dots** — the ten lowest-confidence negative predictions (lowest ensemble probability).
- Each violin shows the kernel density of the ensemble probability for that group, overlaid with the individual predictions as dots.
- Within each violin, the **thick black bar** marks the median of that group and the **thinner grey bar** the mean.
- The **grey dashed horizontal line** marks the decision threshold of 0.5.

We have added the same clarification at the point where the figure is first cited in the running text.

Comment 4

> In page 23, the authors mentioned that the model is trained and evaluated exclusively on human m6Am data. Which human cell lines data?

The reviewer is right that this was a genuine omission, and we thank them for catching it: the original manuscript stated that the model was trained and evaluated on human m6Am data without naming the cell lines.

The positive sites derive from two public miCLIP datasets, and the two source records are annotated differently, so we state both: GSE63753 (Linder et al., the original miCLIP study) is annotated as HEK293, and GSE78040 (Mauer et al.) states that miCLIP was performed on HEK293T cells. We therefore describe the data as HEK293/HEK293T-derived throughout, and give both GEO accessions in the Methods and in the corresponding Limitations sentence.

We have also taken the opportunity to add the fuller provenance chain — m6AmPred, then m6Aminer, then DTC-m6Am — so that a reader can trace the benchmark to its primary source without leaving the paper.
