# Supplementary material for: TriTower-m6Am: A triple-tower heterogeneous deep learning architecture integrating semantic, sequential, and structural information for mRNA N6,2′-O-dimethyladenosine site prediction
Source: PLoS Comput Biol. 2026 Nov 5;22(11):e1014939. doi: 10.1371/journal.pcbi.1014939 (PMC13766619; doi:10.1371/journal.pcbi.1014939)
Supplement: Supplementary file 5 [file pcbi.1014939.s007.pdf]

Response to Reviewers — PCOMPBIOL-D-26-02002 (second revision)

Manuscript: TriTower-m6Am: a triple-tower heterogeneous deep learning architecture integrating semantic, sequential, and structural information for mRNA N6,2'-O-dimethyladenosine site prediction **Decision:** Minor Revision

---

Response to the Editor

We thank the editor and the reviewers for the constructive assessment and for the specific, checkable points. All three reviewers' comments are addressed below.

Completing the previous revision. Reviewer #1's second point — that the manuscript still contains values and statements from the original analysis — identifies a real incompleteness. The first revision recomputed every threshold-dependent value and rewrote the Results and the tables accordingly, but the changes were not propagated to every location, and the four figures that draw on recomputed values were not regenerated. Five places were left behind:

1. The neighbour-only sentence in the edge-type ablation, where the AUC and MCC were updated but the accompanying SN/SP were not.
2. Two AUC values in Table 6 and one MCC value in the node-feature text, which were carried over from the previous analysis instead of re-derived.
3. The caption of Fig. 13, which still described the pre-revision behaviour.
4. Figs. 6, 7, 8 and 13, which we did not regenerate after recomputing the values they display.
5. The S1 Table legend in the Supporting information section of the main text, which still carried the original measurement protocol, described the ensemble row as a sum of the towers, and repeated the sentence "The comparison is therefore favourable to TriTower-m6Am" that Reviewer #3 queries below. The S1 Table file itself was rewritten for this revision; the main-text legend was not.

We have audited every threshold-dependent number in the paper against a from-scratch recomputation under the protocol stated in the Methods, completed the propagation, and regenerated the four figures. The individual corrections are documented under R1.1 and R1.2.

Reviewer #3's question about the efficiency table led us to re-measure that table under a stricter protocol and to measure the two preprocessing steps it had previously only declared excluded. That is documented under R3.1.

Reviewer #1

> I appreciate the substantial revisions and additional analyses. My original concerns have been adequately addressed. […] I have only a few remaining consistency issues that should be corrected before publication.

We thank the reviewer for the careful second reading, and for checking the figures against the tables rather than taking the text at face value.

R1.1 — Statements that still reflect the original interpretation

> Several statements in the revised Results still appear to reflect the original interpretation. For example, the manuscript refers to the AUC difference versus DTC-m6Am as a "larger and more robust" difference and later states that a sensitivity advantage is "immediately apparent", although the revised statistical analysis finds that the AUC difference is not significant and Table

4 reports SN = 0.869 for TriTower-m6Am versus 0.878 for DTC-m6Am. Similarly, the multi-seed section refers to a “reproducible” ranking advantage. These statements should be revised to match the more appropriately cautious interpretation given in the response letter.

The reviewer is right on all three. The second was the most consequential, since it asserted a sensitivity advantage while Table 4 reports DTC-m6Am with the higher sensitivity.

What we changed.

Location	Was	Now
Overall performance	“The **larger and more robust** differences are in ranking quality: AUC (0.776 vs. 0.765) and area under the precision-recall curve, and in model size…”	“We therefore report the sensitivity difference as a **small, threshold-independent trend**. TriTower-m6Am achieved a **slightly higher AUC (0.776 vs. 0.765; not statistically significant)** and **comparable sensitivity (0.869 vs. 0.878)**, using 4.4 times fewer parameters than DTC-m6Am, with every prediction decomposable into its three tower contributions. **Its advantage is therefore competitive performance at substantially lower model size.**”
Radar paragraph	“The **sensitivity advantage** of TriTower-m6Am over all competing methods is **immediately apparent** in the radar profile.”	“Among the compared methods, TriTower-m6Am achieved the **highest AUC, MCC, and F1**. Its sensitivity was **comparable to, not higher than**, that of DTC-m6Am (0.869 vs. 0.878), and it achieved this with roughly one-quarter of the parameters. The radar profile reflects this pattern: top ranking performance with sensitivity on par with the strongest baseline.”
Opening of that paragraph	“The **most telling column** is sensitivity.”	The lead-in sentence is removed; the paragraph opens directly with the matched-specificity comparison.
Multi-seed	“the **ranking advantage is reproducible** across initializations”	“the **ranking metrics are stable** across initializations”

We then audited the full text rather than stopping at the three cited sentences. Two further sentences carried the same problem and have been revised: “the ensemble’s **superior** performance” (now “higher scores”), and, in the Discussion, “the tower’s **advantage** over the non-graph representations” (now “the tower’s **margin** over the non-graph representations is **narrow**”), since the single-tower AUCs in Table 5 differ by only 0.006–0.012 and were not tested for significance.

R1.2 — Figures retaining values from the original analysis

> Several figures appear to retain threshold-dependent values from the original analysis despite the corrected threshold-selection procedure. In particular, the MCC values in Figs. 6–8 do not match the corresponding revised text/tables, and Fig. 13 still shows the previous SN/SP/MCC values rather than the fixed-threshold results described in the revised Results. Please regenerate these figures and check the manuscript throughout for any remaining values from the original analysis.

Confirmed. When we recomputed the threshold-dependent values for the first revision, we updated the text and the tables but did not regenerate these four figures. The other three figures that draw on recomputed values (Figs. 4, 5 and 12) were regenerated at the time, which is why only these four fell out of step.

We regenerated all four figures, and separately re-derived every value they display from the saved out-of-fold and test predictions under the protocol stated in the Methods (balanced out-of-fold MCC-optimal threshold, selected once and applied unchanged to the test set). Two further points came out of that re-derivation:

- **Rounding.** The figures were printing three-decimal labels from four-decimal inputs, so the full-ensemble AUC displayed as 0.775 while the text and Table 4 report 0.776. The figures now use the same round-half-up convention as the tables, and every number in Figs. 6, 7, 8 and 13 agrees with its counterpart in Tables 4, 5 and 6 and in the Results text.
- **A label collision in Fig. 13.** Because the threshold is now held fixed, the three sensitivity bars are equal by construction; the previous labelling offsets, tuned when the three values differed, printed all three labels on top of one another. Sensitivity is now labelled once for the group.

Additional package audit. A separate cross-file check before resubmission identified three remaining inconsistencies in material that also depended on the recomputed thresholds. Figure 12 still showed the pre-revision `pos_weight` sweep while the Results text described the rerun values. The two tower-description paragraphs in Results described RNA-FM and One-Hot BiLSTM with their SN/SP patterns reversed. Table 10 reported four of the six target specificities even though the text summarized the mean difference across all six. We regenerated Figure 12 from the rerun summary, corrected both tower-description paragraphs, added the 0.45 and 0.55 columns to Table 10, and synchronized the clean manuscript, marked manuscript and delivered figure file.

Result of the audit. After these corrections, every threshold-dependent value in Table 4, Table 5, Table 6, the node-feature ablation, the wide-baseline comparison, the multi-seed table, the imbalance evaluation and Table 10 matches the recomputation to the last reported digit.

Revised manuscript text (Overall performance):

> *We therefore report the sensitivity difference as a small, threshold-independent trend. TriTower-m6Am achieved a slightly higher AUC (0.776 vs. 0.765; not statistically significant) and comparable sensitivity (0.869 vs. 0.878), using 4.4 times fewer parameters than DTC-m6Am, with every prediction decomposable into its three tower contributions. Its advantage is therefore competitive performance at substantially lower model size.*

Reviewer #2

> Thank you for your prompt response and addressing my comments. I have no additional concerns with this publication.

We thank the reviewer.

Reviewer #3

Decision: Accept with minor revision. The authors applied my comment that asked them to report about the processing time of TriTower-m6Am against the best previous model DTC-m6Am by providing the supplementary table S1 Table which is good. However, I do not understand why the comparison is favourable to TriTower-m6Am if it has lower number of parameters and higher inference time (ms) and Peak GPU memory (MB) in comparison to DTC-m6Am [...] Therefore, I would prefer further explanation and clarification from authors for this point based on the TriTower-m6Am and DTC-m6Am architectures.

R3.1 — Why fewer parameters, yet higher latency and higher peak memory

The reviewer's question is well posed, and answering it changed our numbers as well as our wording. Two separate things needed attention.

(a) The ambiguous sentence. The S1 Table caption said "The comparison is therefore *favourable to TriTower-m6Am*". We meant that the measurement setup is generous to us, because we exclude two per-window preprocessing steps that TriTower-m6Am needs in deployment and DTC-m6Am does not, so that our reported time is a lower bound on our own cost. Read as a claim about the result, the sentence says the opposite of what the numbers show. It has been rewritten to state plainly that the TriTower rows understate its true deployment cost and are not a like-for-like comparison.

(b) The measurement protocol. Re-measuring the table showed that two different things had been conflated in the original numbers. The ensemble row had not been measured at all: it was *defined* as the maximum of the three tower peaks, which is why it exactly equalled the One-Hot row. And all three towers had been timed in a single process, where `torch.cuda.reset_peak_memory_stats()` resets the high-water mark to the currently allocated bytes rather than to zero, so components measured later inherited the peak of components measured earlier. Each component is now benchmarked in its own process, and the ensemble is measured directly.

Corrected measurements (RTX 3060 12 GB, batch size 1, 20 warm-up + 200 timed passes):

Component	Parameters	Inference time (ms)	Peak GPU memory (MB)
RNA-FM tower	140,974	2.24 ± 0.41	9.89
One-Hot tower	143,489	0.94 ± 0.19	**41.52**
RGCN tower	45,203	8.61 ± 0.68	9.33
TriTower ensemble	329,666	12.41 ± 1.26	**42.35**
DTC-m6Am	1,446,256	3.6–8.5	15.63

A note on the baseline's timing. DTC-m6Am's inference time was bimodal across repeated isolated runs under this protocol: it landed at either about 3.6 ms or about 8.5 ms, with each mode internally reproducible, and at about 23 ms when deterministic cuDNN algorithms were forced. The TriTower-m6Am components showed no comparable variation across the same sessions. We therefore report the baseline as a range rather than as a single value, and we do not rest any claim on the exact ratio.

Under the corrected protocol the numbers are close to those the reviewer worked from: TriTower-m6Am uses **4.4 times fewer parameters** and peaks at **2.71 times the memory**. Its latency is also higher, though by an amount we can only bound: because DTC-m6Am's own inference time is bimodal, the ensemble is between **1.5 and 3.4 times slower** depending on which mode the baseline lands in. The three effects below account for each of those three comparisons, and none of them is an effect of model size.

(c) The architectural explanation.

- **Parameter count is not compute.** The 329,666 figure counts only the three downstream encoders. The frozen RNA-FM backbone, whose 41×640 per-position embeddings the semantic tower consumes, is excluded from every parameter count in the table, yet those embeddings must be materialised and processed. One model trains a quarter as many weights; the other does less work per sample.

- **Latency is the sum of three heterogeneous encoders, not the cost of one wider model.** TriTower-m6Am runs its three towers in series. The RGCN tower dominates the total at 8.61 of the 12.41 ms, and it is the *smallest* of the three at 45K parameters: relational message passing over three edge types on 41-node graphs at batch size 1 is a latency-bound scatter/gather workload that leaves the GPU largely idle. DTC-m6Am is a single homogeneous DenseNet-style stack of dense convolutions, which cuDNN executes with high utilisation.

- **Peak memory at batch size 1 reflects library workspaces, not weights.** This is what answers the reviewer's observation directly. The One-Hot tower's 41.52 MB is essentially all cuDNN recurrent workspace — PyTorch's cuDNN LSTM path reserves roughly 32 MB regardless of the model's size, and disabling cuDNN drops the identical measurement from 41.52 MB to **9.70 MB**. The ensemble contains that same LSTM, so its high-water mark sits just above the One-Hot row (42.35 MB) even though the ensemble's own weight storage is under 1 MB. DTC-m6Am's lower figure reflects that a convolutional network of this size needs no comparable workspace. A striking consequence is that the *fastest* component carries the *largest* peak, which is why peak memory at this batch size cannot be read as a proxy for model size.

  One number in the old table did not move: the One-Hot peak was 41.62 MB before and is 41.52 MB now. That value was not a product of the old protocol — it is a genuine property of that component, and identifying its cause is what resolves the reviewer's question.

(d) The two preprocessing steps, measured. S1 Table previously declared only that the TriTower rows "exclude the cost of RNAfold secondary-structure prediction and of extracting the frozen RNA-FM embeddings". Declaring an exclusion is weaker than quantifying it, so we measured both.

- **RNAfold** secondary-structure prediction costs **2.81 ms** per window (median over 50,000 windows). This was measured with 15 parallel workers, so CPU contention inflates each individual call; the minimum single-call time observed was 1.62 ms. We therefore report 2.81 ms as an upper bound on the uncontended latency.
- **RNA-FM embedding extraction** is the larger cost. A single forward pass at batch size 1 — the deployment case, one new window at a time — takes **18.33 ms and peaks at 391.46 MB**, against 12.41 ms and 42.35 MB for the whole three-tower ensemble. The same step amortises to 2.52 ms per window batched at size 8 (20.12 ms per batch of 8), the setting used for the offline extraction in this study; the two figures are reported separately because they answer different questions.

A complete per-window cost for one new prediction is therefore roughly **33.6 ms and 391 MB** against 3.6–8.5 ms and 15.63 MB for DTC-m6Am, most of it in a preprocessing step rather than in the architecture being compared.

What we changed. The main text now reports both sides of the trade: TriTower-m6Am uses a four-fold smaller parameter count and provides three complementary representations, at the cost of a heavier pipeline. The architectural reasons are given in brief, with the per-component breakdown and the workspace measurement in S1 Table.

R3.2 — Abbreviations used without explanation

> I am still seeing some abbreviations used for the first time in the paper body without explanation such as Low-Rank Adaptation (LoRA), Centered Kernel Alignment (CKA), CAPAM, FTO, GELU, RGCN, PCIF1, RNA-FM.

All eight are now expanded at first mention — in the Abstract where the term first appears there, and again at first mention in the body, as the journal's convention requires for abstract abbreviations.

Term	First mention	Now reads
PCIF1	Abstract	"PCIF1 (phosphorylated CTD-interacting factor 1)"

Term	First mention	Now reads
RNA-FM	Abstract	“RNA-FM, a pretrained RNA foundation model”
RGCN	Abstract	“RGCN, a relational graph convolutional network”
BiLSTM	Abstract	“One-Hot BiLSTM, bidirectional long short-term memory”
CAPAM	Introduction	“CAPAM for cap-specific adenosine methyltransferase”
FTO	Introduction	“FTO (fat mass and obesity-associated protein)”
GELU	Methods	“GELU (Gaussian error linear unit)”
CKA	Results	“CKA (centered kernel alignment)”
LoRA	Discussion	“LoRA (low-rank adaptation)”

Because the reviewer’s list was introduced with “such as”, we treated it as non-exhaustive and ran a systematic scan for abbreviations whose first use precedes their explanation. That scan covered the whole manuscript — including section headings, table cells and figure captions, which our first check had not — and found a further fourteen, all now expanded at first mention:

Term	First use	Now reads
AUC, MCC	Abstract	“an area under the ROC curve (AUC) of 0.776 and a Matthews correlation coefficient (MCC) of 0.425”
PR	Results section heading	heading now reads “Overall performance: ROC and precision–recall analysis”
IG	Results section heading	heading now reads “Interpretability: BellPooling, integrated gradients, and ensemble weights”
EMA	Methods hyperparameter table	“EMA (exponential moving average) decay”
miCLIP	Introduction	“miCLIP (methylation individual-nucleotide-resolution cross-linking and immunoprecipitation)”
m6A, m5C	Introduction	“N6-methyladenosine (m6A) or 5-methylcytosine (m5C)”
ncRNA	Methods	“non-coding RNA (ncRNA)”
SHAP	Introduction	“SHAP (SHapley Additive exPlanations)”
SS	Introduction	“Secondary structure (SS)”
CTD	Abstract, Introduction	“phosphorylated C-terminal domain (CTD)-interacting factor 1”
UTR	Discussion	“5’ untranslated region (5’UTR)”
m1A	Discussion	“N1-methyladenosine (m1A)”

Term	First use	Now reads
SHAPE-MaP, DMS-seq	Discussion	"SHAPE-MaP and DMS-seq, chemical-probing assays that measure RNA structure in vitro"
w/o, pp	Table 5 header	both now defined in the table caption

Two terms we had previously recorded as already defined — **EMA** and **IG** — in fact appear first in a hyperparameter table and a section heading respectively, ahead of their prose definitions; both are now handled, and we have corrected that claim. The others in that group (ACC, AP, GAT, OOF, ROC) were re-checked and do hold.

We also brought the end-of-paper **List of abbreviations** back into agreement with the text: it had listed m7G, which does not appear anywhere in the manuscript, and omitted fifteen terms that the text uses, including the ones added above.

Summary of changes

#	Change	Where
1	Wording aligned with the reported statistics (5 sentences)	Abstract, Results, Discussion
2	Figs. 6, 7, 8 and 13 regenerated from the recomputed values	Figures
3	Omissions from the previous revision completed (neighbour-only SN/SP)	Results
4	Values not re-derived in the previous revision now carry the correct rounding (2 AUC, 1 MCC)	Table 6, Results
5	Fig. 13 caption, omitted in the previous revision, now updated (SN is constant at the fixed threshold)	Figure caption
6	Efficiency table re-measured with isolated processes; architectural explanation added; main-text S1 legend corrected to match the S1 file	S1 Table, Results
7	Abbreviations expanded at first mention throughout, including section headings and table cells; end-of-paper list reconciled with the text	Abstract, Introduction, Methods, Results, Discussion
8	The two preprocessing steps quantified (RNAfold 2.81 ms, RNA-FM 18.33 ms / 391 MB per window)	S1 Table, Results

We hope the manuscript is now suitable for publication, and we thank all three reviewers for a thorough and

productive review process.
