# Supplementary material for: TriTower-m6Am: A triple-tower heterogeneous deep learning architecture integrating semantic, sequential, and structural information for mRNA N6,2′-O-dimethyladenosine site prediction
Source: PLoS Comput Biol. 2026 Nov 5;22(11):e1014939. doi: 10.1371/journal.pcbi.1014939 (PMC13766619; doi:10.1371/journal.pcbi.1014939)
Supplement: Supplementary file 6 [file pcbi.1014939.s002.docx]

1. The RGCN uses secondary structures predicted by RNAfold separately for each 41-nt window (Materials and methods, pp. 3–4). Therefore, the predicted structure represents the isolated 41-nt fragment and excludes any interactions with nucleotides outside the ±20-nt window. While the authors acknowledge the general uncertainty of RNAfold predictions in the Limitations (p. 23), they do not address whether folding an isolated 41-nt fragment adequately represents the structural environment of the same region within the full-length mRNA. Please provide a rationale for using the 41-nt window for secondary-structure prediction and discuss how the exclusion of longer-range interactions may affect the structural features used by the RGCN.
2. The RGCN is presented as the structural tower, but the node-feature ablation (Fig. 8, pp. 14–15) shows that nucleotide identity and positional encoding have the largest effects on performance: removing them reduces AUC from 0.764 to 0.698 and 0.724, respectively. In contrast, removing secondary-structure state changes AUC from 0.764 to 0.762, while removing pairing geometry increases AUC slightly to 0.766. In the edge-type ablation (Table 6, pp. 12–13), the backbone-only model reaches AUC = 0.757 compared with 0.768 for the full RGCN; however, “backbone-only” refers only to the retained edge type, while the full node-feature representation, including structure-derived features, remains available to the model. Therefore, the current ablations do not clearly establish how much of the RGCN's predictive performance is specifically attributable to secondary-structure information rather than sequence and positional information. The authors should revise this interpretation or provide an analysis that isolates the contribution of secondary-structure information.
3. The manuscript states that m6Am occurs at the first transcribed nucleotide (Introduction, p. 3), while each sample is represented as a 41-nt window containing 20 nucleotides upstream and downstream of the candidate site (Materials and methods, pp. 3–4). Given that m6Am is described here as occurring at the first transcribed nucleotide, it is unclear what the 20 upstream nucleotides represent in these windows. The authors should clearly state how the upstream sequence was defined in the benchmark and explain its biological relevance to the prediction task.
4. The main performance improvement over DTC-m6Am is reported as AUC = 0.776 versus 0.765, a difference of 0.011, while sensitivity increases from 0.800 to 0.888 with similar specificity (0.530 versus 0.522) (Table 4, pp. 8–9). Although the authors evaluate the stability of TriTower-m6Am across three random seeds (Table 8, pp. 16–18), no confidence intervals or direct statistical comparison with DTC-m6Am are provided. Given the relatively small AUC difference on a 640-sample test set, the authors should provide appropriate uncertainty estimates and a statistical comparison to establish whether the reported improvement over DTC-m6Am is robust.
5. The interpretation of the standalone-model results is inconsistent with Table 5. The manuscript states on p. 10 that “no single encoder in the comparison clears MCC = 0.40” and uses this to support the benefit of combining heterogeneous representations. However, Table 5 reports MCC = 0.439 for the standalone RGCN, compared with 0.440 for the full ensemble (BiLSTM also reaches 0.401). The authors should correct this statement and reconsider the corresponding interpretation of the ensemble improvement.

**Minor comments:** The manuscript contains several additional reporting inconsistencies that should be corrected. On p. 11, the text states that removing BiLSTM produces the smallest AUC loss, whereas Table 5 shows the smallest decrease after removing RNA-FM (0.776 to 0.773). On p. 19, the external evaluation is described as being performed “without any retraining or threshold adjustment,” but the same section reports MCC-optimal thresholds of 0.48, 0.67, and 0.81 for the 1:1, 1:10, and 1:100 settings, respectively; the threshold-selection procedure should be clarified. Figure 2 also includes ACC although the corresponding values are not reported in Table 4.
