# Supplementary material for: TriTower-m6Am: A triple-tower heterogeneous deep learning architecture integrating semantic, sequential, and structural information for mRNA N6,2′-O-dimethyladenosine site prediction
Source: PLoS Comput Biol. 2026 Nov 5;22(11):e1014939. doi: 10.1371/journal.pcbi.1014939 (PMC13766619; doi:10.1371/journal.pcbi.1014939)
Supplement: Peer review history

# Peer review: Decision Letter 0

**DOI:** 10.1371/journal.pcbi.1014939.r001

**Authors:** Ilya Ioshikhes, Sarath Chandra Janga

11 Sep 2026

TriTower-*m6Am* : a triple-tower heterogeneous deep learning architecture integrating semantic, sequential, and structural information for mRNA *N* ^6^,2'-O-dimethyladenosine site prediction

PLOS Computational Biology

Dear Dr. Jia,

Thank you for submitting your manuscript to PLOS Computational Biology. After careful consideration, we feel that it has merit but does not fully meet PLOS Computational Biology's publication criteria as it currently stands. Therefore, we invite you to submit a revised version of the manuscript that addresses the points raised during the review process.

Please submit your revised manuscript by Nov 10 2026 11:59PM. If you will need more time than this to complete your revisions, please reply to this message or contact the journal office at ploscompbiol@plos.org. When you're ready to submit your revision, log on to https://www.editorialmanager.com/pcompbiol/ and select the 'Submissions Needing Revision' folder to locate your manuscript file.

Please include the following items when submitting your revised manuscript:

* A letter that responds to each point raised by the editor and reviewer(s). You should upload this letter as a separate file labeled 'Response to Reviewers'. This file does not need to include responses to formatting updates and technical items listed in the 'Journal Requirements' section below.

* A marked-up copy of your manuscript that highlights changes made to the original version. You should upload this as a separate file labeled 'Revised Manuscript with Track Changes'.

* An unmarked version of your revised paper without tracked changes. You should upload this as a separate file labeled 'Manuscript'.

We look forward to receiving your revised manuscript.

Kind regards,

Sarath Chandra Janga, Ph.D

Academic Editor

PLOS Computational Biology

Ilya Ioshikhes

Section Editor

PLOS Computational Biology

**Additional Editor Comments (if provided):**

Dear Dr. Jia

Thank you for submitting your manuscript to PLoS Computational Biology. While the reviewers generally agree that the study addresses an important scientific problem with a potentially useful and scientifically sound approach, and the reported results are promising, they have raised several concerns which impede the enthusiasm to publish the work in its current form unless it is significantly revised to generate a far stronger manuscript.

In particular,, several substantive and presentation-related issues should be addressed before the manuscript is suitable for publication. Most importantly, the structural tower relies on RNAfold predictions from isolated 41-nt sequence windows, and the current ablation analyses does not clearly establish the extent to which the RGCN's performance is specifically attributable to secondary-structure information. In addition, although TriTower-m6Am outperforms DTC-m6Am, the reported AUC improvement is relatively modest (0.776 vs. 0.765) and is not supported by confidence intervals or direct statistical significance testing. There is also an inconsistency between the manuscript's interpretation of the standalone models and the results presented in Table 5. Beyond these scientific concerns, the manuscript has structural and editorial issues: some Materials and Methods sections appear unnecessary, other important methodological details are insufficiently described or omitted, at least one figure is not adequately referenced or discussed in the main text, and multiple typographical errors require correction.

Overall, the manuscript has the potential to make a meaningful contribution, but additional analyses, clearer methodological reporting, reconciliation of inconsistencies, and careful editorial revision are needed to adequately support the main conclusions. Therefore, I recommend Major Revision. Please note that a revised version will again be sent to reviewers for further review.

We look forward to receiving your revised version

Sarath Janga, PhD

**Journal Requirements:**

If the reviewer comments include a recommendation to cite specific previously published works, please review and evaluate these publications to determine whether they are relevant and should be cited. There is no requirement to cite these works unless the editor has indicated otherwise.

1) We ask that a manuscript source file is provided at Revision. Please upload your manuscript file as a .doc, .docx, .rtf or .tex. If you are providing a .tex file, please upload it under the item type u2018LaTeX Source Fileu2019 and leave your .pdf version as the item type u2018Manuscriptu2019.

2) Please upload all main figures as separate Figure files in .tif or .eps format. For more information about how to convert and format your figure files please see our guidelines:

https://journals.plos.org/ploscompbiol/s/figures

3) Please upload a copy of Figure Fig 1-15 which you refer to in your text on pages 4, 10, 11, 12, 14, 15, 16, 18, and 19. Or, if the figure is no longer to be included as part of the submission please remove all reference to it within the text.

4) We notice that your supplementary Tables are included in the manuscript file. Please remove them and upload them with the file type 'Supporting Information'. Please ensure that each Supporting Information file has a legend listed in the manuscript after the references list.

**Reviewers' comments:**

Reviewer's Responses to Questions

**Comments to the Authors:**

**Please note here if the review is uploaded as an attachment.**

Reviewer #1: This study presents TriTower-m6Am, which integrates RNA-FM, BiLSTM, and RGCN-based representations for m6Am site prediction. The framework is interesting and the manuscript includes extensive ablation analyses; however, several methodological and interpretive issues should be addressed before the main conclusions are fully supported. Please see the attached review for detailed comments.

Reviewer #2: If the modification only occurs on the first transcribed nucleotide, how do you center the 41-nt window at the candidate position? It would seem like you only have the 20-nt downstream to utilize. For clarity I think this needs to be explicitly expanded upon.

It’s unclear to me where the 640 dimension of your matrix comes from. Please elaborate further.

While your model does have higher metrics than competing models, especially as you’ve observed in the SN. I think a more thorough examination of the model predictions would greatly improve the clarity of your argument. The addition of a table or venn-diagram would enhance the voracity of your claims, by clearly illustrating the samples that your model is correctly identifying that the mother models are leaving out.

Missing reference and explanation of Figure 4 in running text.

In the discussion of Figure 5, the affect that removing components has on specificity needs to be explicitly stated. I think the explanation is eluded to in the AUC results, but should be addressed in the prose.

The results presented in Figure 10 increase my concern about the 20-nt upstream described in a previous comment. These are clearly not randomly determined but are heavily GC skewed. I think this needs to be more explitily discussed.

Typo in Figure 1a: RNA-FM Twoer should be Tower

Please explain acronyms before you use them for the first time or provide definition in parentheses, GAT, line 97.

I don’t think Equation 5 is necessary. I think MCC is relatively well known and or can be easily looked up.

The angle labels on Fig 2 clutter the figure, please remove these.

Reviewer #3: This paper presents TriTower-m6Am, a triple-tower architecture model that combines three independent encoders: a pretrained RNA language model for semantic patterns (RNA-FM with BellPooling), a bidirectional LSTM for local nucleotide order (One-Hot BiLSTM), and a relational graph convolutional network for the structural fold (RGCN with three typed edges) for predicting N6,2'-O-dimethyladenosine (m6Am) sites. The model fuses the outputs of the three encoders by AUC-weighted voting, so the contribution of each tower to a given prediction is a readable number rather than a hidden parameter or black box that emits a prediction without explaining which features drove it. The paper is well written with rich presented analysis and the results of the presented paper approach are promising. Therefore, I recommend publishing this paper in PLOS Computational Biology Journal after addressing the following question:

-I see the authors have compared the performance of TriTower-m6Am to the best previous model (DTC-m6Am) in terms of AUC, MCC, SN, SP, and F1 as reported in Table 4. However, I do not see a comparison between the processing time of TriTower-m6Am and the DTC-m6Am. I think providing such comparison would be also worthy.

Minors:

-The authors provide a list of abbreviations in the end of the paper which is nice. However, I prefer writing the full expression that the abbreviation refers to in the paper body instead of relying only on the list of abbreviations as this will save the time for the reader.

-Figure 14 is missing legend. What do dark orang and blue dots represent for positive and negative predictions respectively? Also, what black and blue lines refer to? More clarification should be provided by the authors.

- In page 23, the authors mentioned that the model is trained and evaluated exclusively on human m6Am data. Which human cell lines data?

**********

**Have the authors made all data and (if applicable) computational code underlying the findings in their manuscript fully available?**

The PLOS Data policy requires authors to make all data and code underlying the findings described in their manuscript fully available without restriction, with rare exception (please refer to the Data Availability Statement in the manuscript PDF file). The data and code should be provided as part of the manuscript or its supporting information, or deposited to a public repository. For example, in addition to summary statistics, the data points behind means, medians and variance measures should be available. If there are restrictions on publicly sharing data or code —e.g. participant privacy or use of data from a third party—those must be specified.

Reviewer #1: Yes

Reviewer #2: Yes

Reviewer #3: Yes

**********

PLOS authors have the option to publish the peer review history of their article (what does this mean?). If published, this will include your full peer review and any attached files.

If you choose “no”, your identity will remain anonymous but your review may still be made public.

**Do you want your identity to be public for this peer review?**  For information about this choice, including consent withdrawal, please see our Privacy Policy.

Reviewer #1: No

Reviewer #2: **Yes:**  Alexander Krohannon

Reviewer #3: **Yes:**  Doaa Hassan Salem

[NOTE: If reviewer comments were submitted as an attachment file, they will be attached to this email and accessible via the submission site. Please log into your account, locate the manuscript record, and check for the action link "View Attachments". If this link does not appear, there are no attachment files.]

**Figure resubmission:**

**Reproducibility:**

?>

Attachment Submitted filename: Review_Comments_TriTower-m6Am.docx

Attachment Submitted filename: review1.pdf

# Author comment: Author response to Decision Letter 1

**DOI:** 10.1371/journal.pcbi.1014939.r002

19 Sep 2026

Attachment Submitted filename: Response_to_Reviewers.pdf

# Peer review: Decision Letter 1

**DOI:** 10.1371/journal.pcbi.1014939.r003

**Authors:** Ilya Ioshikhes, Sarath Chandra Janga

21 Oct 2026

PCOMPBIOL-D-26-02002R1

TriTower-*m6Am* : a triple-tower heterogeneous deep learning architecture integrating semantic, sequential, and structural information for mRNA *N* ^6^,2'-O-dimethyladenosine site prediction

PLOS Computational Biology

Dear Dr. Jia,

Thank you for submitting your manuscript to PLOS Computational Biology. After careful consideration, we feel that it has merit but does not fully meet PLOS Computational Biology's publication criteria as it currently stands. Therefore, we invite you to submit a revised version of the manuscript that addresses the points raised during the review process.

Please submit your revised manuscript by Nov 20 2026 11:59PM. If you will need more time than this to complete your revisions, please reply to this message or contact the journal office at ploscompbiol@plos.org. When you're ready to submit your revision, log on to https://www.editorialmanager.com/pcompbiol/ and select the 'Submissions Needing Revision' folder to locate your manuscript file.

Please include the following items when submitting your revised manuscript:

* A letter that responds to each point raised by the editor and reviewer(s). You should upload this letter as a separate file labeled 'Response to Reviewers'. This file does not need to include responses to formatting updates and technical items listed in the 'Journal Requirements' section below.

* A marked-up copy of your manuscript that highlights changes made to the original version. You should upload this as a separate file labeled 'Revised Manuscript with Track Changes'.

* An unmarked version of your revised paper without tracked changes. You should upload this as a separate file labeled 'Manuscript'.

If you would like to make changes to your financial disclosure, competing interests statement, or data availability statement, please make these updates within the submission form at the time of resubmission. Guidelines for resubmitting your figure files are available below the reviewer comments at the end of this letter.

As the corresponding author, your ORCID iD is verified in the submission system and will appear in the published article. PLOS supports the use of ORCID, and we encourage all coauthors to register for an ORCID iD and use it as well. Please encourage your coauthors to verify their ORCID iD within the submission system before final acceptance, as unverified ORCID iDs will not appear in the published article. Only the individual author can complete the verification step; PLOS staff cannot verify ORCID iDs on behalf of authors.

We look forward to receiving your revised manuscript.

Kind regards,

Sarath Chandra Janga, Ph.D

Academic Editor

PLOS Computational Biology

Ilya Ioshikhes

Section Editor

PLOS Computational Biology

**Additional Editor Comments (if provided):**

Dear Dr. Jia

Reviewers have appreciated the revisions made in the previous round but have indicated that some of the language and discussion in the manuscript doesn't reflect the new results, in addition to few other minor concerns such as missing abbreviations noted by them. In lieu of these identified issues, I would like to invite you to make changes and submit a revision version of the manuscript addressing all the raised concerns in two weeks.

Sincerely

Sarath

**Reviewers' comments:**

Reviewer's Responses to Questions

**Comments to the Authors:**

**Please note here if the review is uploaded as an attachment.**

Reviewer #1: I appreciate the substantial revisions and additional analyses. My original concerns have been adequately addressed. In particular, the new structure-permutation analysis, clarification of the 41-nt benchmark context, and direct statistical comparison with DTC-m6Am substantially improve the manuscript. I have only a few remaining consistency issues that should be corrected before publication.

1. Several statements in the revised Results still appear to reflect the original interpretation. For example, the manuscript refers to the AUC difference versus DTC-m6Am as a “larger and more robust” difference and later states that a sensitivity advantage is “immediately apparent,” although the revised statistical analysis finds that the AUC difference is not significant and Table 4 reports SN = 0.869 for TriTower-m6Am versus 0.878 for DTC-m6Am. Similarly, the multi-seed section refers to a “reproducible” ranking advantage. These statements should be revised to match the more appropriately cautious interpretation given in the response letter.

2. Several figures appear to retain threshold-dependent values from the original analysis despite the corrected threshold-selection procedure. In particular, the MCC values in Figs. 6–8 do not match the corresponding revised text/tables, and Fig. 13 still shows the previous SN/SP/MCC values rather than the fixed-threshold results described in the revised Results. Please regenerate these figures and check the manuscript throughout for any remaining values from the original analysis.

Reviewer #2: Thank you for your prompt response and addressing my comments. I have no additional concerns with this publication.

Reviewer #3: Paper title: TriTower-m6Am: a triple-tower heterogeneous deep learning architecture integrating semantic, sequential, and structural information for N6,2'-O-dimethyladenosine site prediction

Reviewer decision: Accept with minor revision

The authors applied my comment that asked them to report about the processing time of TriTower-m6Am against the best previous model DTC-m6Am by providing the supplementary table S1 Table which is good. However, I do not understand why the comparison is favorable to TriTower-m6Am if it has lower number of parameters and higher inference time (ms) and Peak GPU memory (MB) in comparison to DTC-m6Am (The inference time and Peak GPU memory of TriTower-m6Am are 10.38 ms and 41.62 MB respectively, while the inference time and Peak GPU memory of DTC-m6Am are 8.34 ms and 21.29 MB respectively)? Therefore, I would prefer further explanation and clarification from authors for this point based on the TriTower-m6Am and DTC-m6Am architectures.

Minors:

I am still seeing some abbreviations used for the first time in the paper body without explanation such as Low-Rank Adaptation (LoRA), Centered Kernel Alignment (CKA), CAPAM, FTO, GELU, RGCN, PCIF1, RNA-FM.

**********

**Have the authors made all data and (if applicable) computational code underlying the findings in their manuscript fully available?**

The PLOS Data policy requires authors to make all data and code underlying the findings described in their manuscript fully available without restriction, with rare exception (please refer to the Data Availability Statement in the manuscript PDF file). The data and code should be provided as part of the manuscript or its supporting information, or deposited to a public repository. For example, in addition to summary statistics, the data points behind means, medians and variance measures should be available. If there are restrictions on publicly sharing data or code —e.g. participant privacy or use of data from a third party—those must be specified.

Reviewer #1: Yes

Reviewer #2: Yes

Reviewer #3: Yes

**********

PLOS authors have the option to publish the peer review history of their article (what does this mean?). If published, this will include your full peer review and any attached files.

If you choose “no”, your identity will remain anonymous but your review may still be made public.

**Do you want your identity to be public for this peer review?**  For information about this choice, including consent withdrawal, please see our Privacy Policy.

Reviewer #1: No

Reviewer #2: No

Reviewer #3: **Yes:**  Doaa Hassan Salem

[NOTE: If reviewer comments were submitted as an attachment file, they will be attached to this email and accessible via the submission site. Please log into your account, locate the manuscript record, and check for the action link "View Attachments". If this link does not appear, there are no attachment files.]

**Figure resubmission:**

**Reproducibility:**

To enhance the reproducibility of your results, we recommend that authors of applicable studies deposit laboratory protocols in protocols.io, where a protocol can be assigned its own identifier (DOI) such that it can be cited independently in the future. Additionally, PLOS ONE offers an option to publish peer-reviewed clinical study protocols. Read more information on sharing protocols at https://plos.org/protocols?utm_medium=editorial-email&utm_source=authorletters&utm_campaign=protocols

Attachment Submitted filename: review2.pdf

# Author comment: Author response to Decision Letter 2

**DOI:** 10.1371/journal.pcbi.1014939.r004

22 Oct 2026

Attachment Submitted filename: RESPONSE_TO_REVIEWERS_R2.pdf

# Editor report: Decision Letter 2

**DOI:** 10.1371/journal.pcbi.1014939.r005

**Authors:** Ilya Ioshikhes, Sarath Chandra Janga

26 Oct 2026

Dear Dr Jia,

We are pleased to inform you that your manuscript 'TriTower-*m6Am* : a triple-tower heterogeneous deep learning architecture integrating semantic, sequential, and structural information for mRNA *N* ^6^,2'-O-dimethyladenosine site prediction' has been provisionally accepted for publication in PLOS Computational Biology.

Before your manuscript can be formally accepted you will need to complete some formatting changes, which you will receive in a follow up email. A member of our team will be in touch with a set of requests.

Please note that your manuscript will not be scheduled for publication until you have made the required changes, so a swift response is appreciated.

IMPORTANT: The editorial review process is now complete. PLOS will only permit corrections to spelling, formatting or significant scientific errors from this point onwards. Requests for major changes, or any which affect the scientific understanding of your work, will cause delays to the publication date of your manuscript.

Should you, your institution's press office or the journal office choose to press release your paper, you will automatically be opted out of early publication. We ask that you notify us now if you or your institution is planning to press release the article. All press must be co-ordinated with PLOS.

Thank you again for supporting Open Access publishing; we are looking forward to publishing your work in PLOS Computational Biology.

Best regards,

Sarath Chandra Janga, Ph.D

Academic Editor

PLOS Computational Biology

Ilya Ioshikhes

Section Editor

PLOS Computational Biology

***********************************************************

# Editor report: Acceptance letter

**DOI:** 10.1371/journal.pcbi.1014939.r006

**Authors:** Ilya Ioshikhes, Sarath Chandra Janga

PCOMPBIOL-D-26-02002R2

TriTower-*m6Am* : a triple-tower heterogeneous deep learning architecture integrating semantic, sequential, and structural information for mRNA *N* ^6^,2'-O-dimethyladenosine site prediction

Dear Dr Jia,

I am pleased to inform you that your manuscript has been formally accepted for publication in PLOS Computational Biology. Your manuscript is now with our production department and you will be notified of the publication date in due course.

The corresponding author will soon be receiving a typeset proof for review, to ensure errors have not been introduced during production. Please review the PDF proof of your manuscript carefully, as this is the last chance to correct any errors. Please note that major changes, or those which affect the scientific understanding of the work, will likely cause delays to the publication date of your manuscript.

Soon after your final files are uploaded, unless you have opted out, the early version of your manuscript will be published online. The date of the early version will be your article's publication date. The final article will be published to the same URL, and all versions of the paper will be accessible to readers.

For Research, Software, and Methods articles, you will receive an invoice from PLOS for your publication fee after your manuscript has reached the completed accept phase. If you receive an email requesting payment before acceptance or for any other service, this may be a phishing scheme. Learn how to identify phishing emails and protect your accounts at https://explore.plos.org/phishing.

Thank you again for supporting PLOS Computational Biology and open-access publishing. We are looking forward to publishing your work!

With kind regards,

Janani Seenivasan

PLOS Computational Biology | Carlyle House, Carlyle Road, Cambridge CB4 3DN | United Kingdom ploscompbiol@plos.org | Phone +44 (0) 1223-442824 | ploscompbiol.org | @PLOSCompBiol
